# Deciphering the Selectivity of the Electrochemical CO_2_ Reduction to CO by a Cobalt Porphyrin Catalyst in Neutral Aqueous Solution: Insights from DFT Calculations

**DOI:** 10.1002/open.202200254

**Published:** 2023-02-06

**Authors:** Yu‐Chen Cao, Le‐Le Shi, Man Li, Bo You, Rong‐Zhen Liao

**Affiliations:** ^1^ Key Laboratory of Material Chemistry for Energy Conversion and Storage Ministry of Education Hubei Key Laboratory of Bioinorganic Chemistry and Materia Medica Hubei Key Laboratory of Materials Chemistry and Service Failure School of Chemistry and Chemical Engineering Huazhong University of Science and Technology Wuhan 430074 P. R. China

**Keywords:** CO_2_ reduction, cobalt-porphyrin, density functional calculations, reaction mechanisms, selectivity

## Abstract

Density functional theory (DFT) calculations were conducted to investigate the cobalt porphyrin‐catalyzed electro‐reduction of CO_2_ to CO in an aqueous solution. The results suggest that Co^II^−porphyrin (Co^II^−L) undertakes a ligand‐based reduction to generate the active species Co^II^−L⋅^−^, where the Co^II^ center antiferromagnetically interacts with the ligand radical anion. Co^II^−L⋅^−^ then performs a nucleophilic attack on CO_2_, followed by protonation and a reduction to give Co^II^−L−COOH. An intermolecular proton transfer leads to the heterolytic cleavage of the C−O bond, producing intermediate Co^II^−L−CO. Subsequently, CO is released from Co^II^−L−CO, and Co^II^−L is regenerated to catalyze the next cycle. The rate‐determining step of this CO_2_RR is the nucleophilic attack on CO_2_ by Co^II^−L⋅^−^, with a total barrier of 20.7 kcal mol^−1^. The competing hydrogen evolution reaction is associated with a higher total barrier. A computational investigation regarding the substituent effects of the catalyst indicates that the CoPor−R3 complex is likely to display the highest activity and selectivity as a molecular catalyst.

## Graphical abstract

**The cobalt(II) porphyrin‐catalyzed electro‐reduction of CO_2_ to CO** was investigated by DFT calculations regarding the mechanism of the in an aqueous solution. The rate‐determining step of this CO_2_RR is the nucleophilic attack on CO_2_ by Co^II^−L⋅^−^, with a total barrier lower than that of the competing hydrogen evolution reaction. Substituent effects of the catalyst indicate that the CoPor−R3 complex is likely to display the highest activity and CO selectivity as a molecular catalyst.

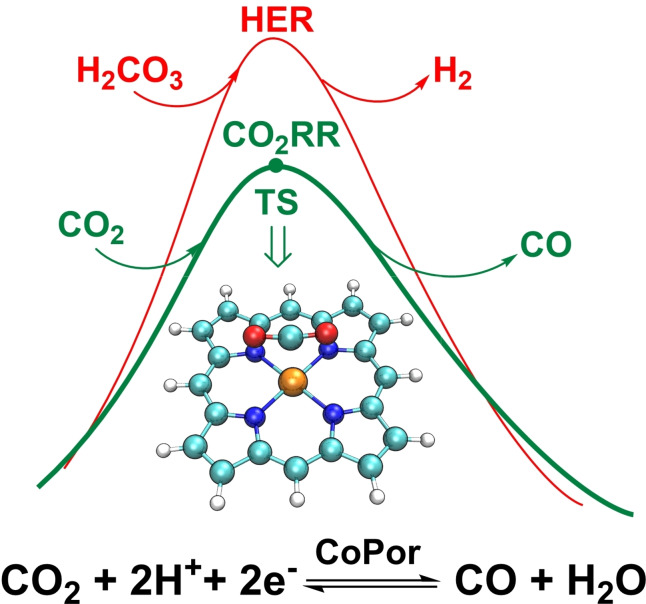

## Introduction

**Scheme 1:**
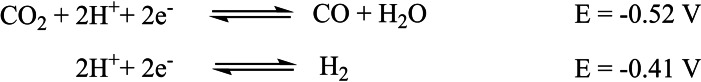
Experimental reduction potentials (vs SHE) at pH=7 in aqueous solutions.

Electrochemical carbon dioxide reduction^[1]^ (CO_2_RR) has been widely acknowledged as a promising and environmentally benign strategy to transform CO_2_ into value‐added C1 products^[2]^ such as carbon monoxide (CO). Due to the electrochemical inertness of CO_2_, proton‐coupled reduction approaches could contribute to decreasing the required potentials (Scheme 1). However, an obvious concern comes from the unavoidable competitive hydrogen evolution reaction (HER),^[3]^ which is thermodynamically favored over CO_2_RR since the redox potential of HER is −0.41 V, more positive than that of CO_2_RR to CO (−0.52 V).^[4]^ Consequently, considerable efforts have been devoted to developing effective catalysts to improve the efficiency and product selectivity of CO_2_RR in the past decades.

So far, various types of transition metal catalysts (both molecular and heterogeneous),^[5]^ such as Fe,[^5h^, ^6^] Co,[^5g^, ^7^] Ni,[^6d^, ^8^] Zn,^[9]^ and Cu‐related^[10]^ structures, have been reported to catalyze the electro‐reduction of CO_2_ to CO. One advantage of homogeneous molecular catalysts is that it is easy to modify their structures to tune the catalytic activity and selectivity.[^3^, ^11^] Nevertheless, an inevitable drawback is that organic solvents are required in the CO_2_RR to diminish the competitive HER and also the degradation of the catalyst. In contrast, heterogeneous catalysts involving transition metal macrocycle units tend to be applied in an aqueous solution. Moreover, strategies such as changing the substituents of the molecular building blocks[^5e^, ^7b^, ^12^] and introducing support material[^7a^, ^7b^, ^13^] are widely employed in the structural modification of the heterogeneous catalysts to achieve high catalytic performance.

**Scheme 2:**
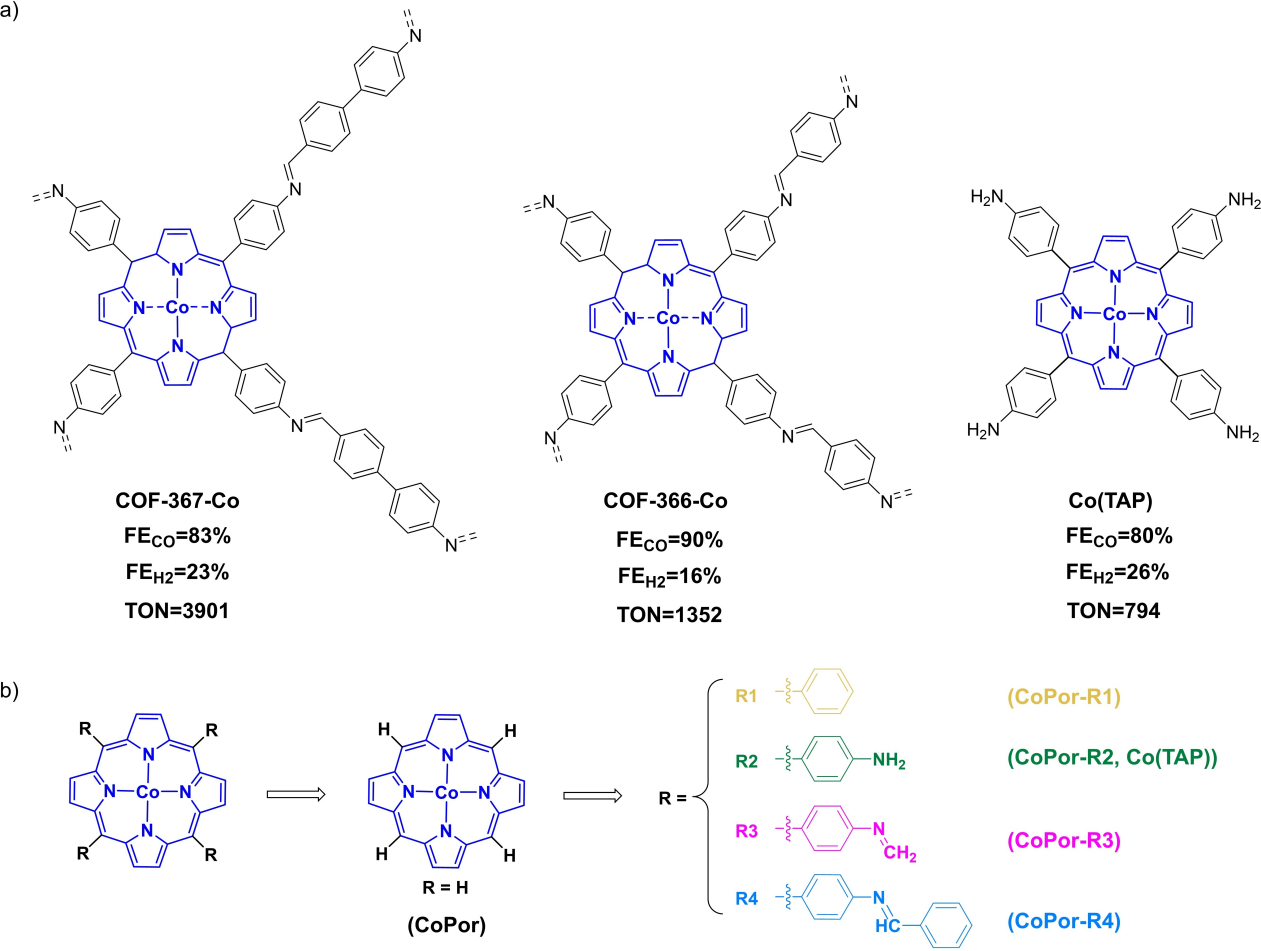
a) Schematic structures of the cobalt(II)‐porphyrin building units in COFs. Here, only one cobalt core for each catalyst is presented. b) Simplified cobalt(II) porphyrin bearing different substituents, with a total charge of 0.

In 2015, Chang and coworkers reported an interesting work that used covalent organic frameworks (COFs) involving modified cobalt porphyrin building units as the electrochemical CO_2_RR catalyst.^[14]^ As shown in Scheme 2a, **COF‐367‐Co**, in which the iminophenyl‐substituted cobalt‐porphyrin building units are linked through imine bonds with biphenyl groups, displayed a fantastic performance in the electrochemical CO_2_ reduction to CO in neutral aqueous solution. Experiments (pH=7.3) indicate a long‐term Faradaic efficiency for CO (FE_CO_ for a 24‐hour period) of 83 % with the TON up to 3901. Besides, **COF‐366‐Co**, where the cobalt‐porphyrin units are linked through imine bonds with phenyl groups, shows slightly higher 24 h period Faradaic efficiency for CO (FE_CO_=90 %) yet lower electroactivity (TON=1352). Notably, both COFs illustrate much better catalytic capability in catalyzing CO_2_RR in an aqueous solution than the molecular catalyst **Co(TAP)** (TON=794) that bears four aniline substitutes. Clearly, the precise construction of the active sites in the frameworks through modulation of the building units could contribute to the well‐stabilized catalysts that enable prolonged effective CO_2_ conversion and minimize the proton‐induced HER.[^14^, ^15^]

Inspired by this experimental work, herein, density functional theory (DFT) calculations have been conducted to shed light on the mechanism of the COFs catalyzed electrochemical CO_2_RR. To simplify the calculation, only the cobalt(II) porphyrin core unit was included in the study. Firstly, we concentrate on the mechanism of CO_2_RR catalyzed with the model catalyst **CoPor** (Scheme 2b). Different reaction pathways have been evaluated to reveal the most feasible one. The undesirable hydrogen evolution reaction mechanism was also explored. Furthermore, substituent effects on the catalytic ability have been investigated by incorporating four different groups (R1, R2, R3, and R4, as shown in Scheme 2b) onto the **CoPor** model. Noteworthy, catalyst **CoPor−R2** is the molecular **Co(TAP)**, and catalysts **CoPor−R3** and **CoPor−R4** originate from the core building unit of **COF‐366‐Co** and **COF‐367‐Co**. Overall, we expect that the herein‐presented computational study provides a deeper insight into the mechanism of the electrochemical transformation of CO_2_ to CO, and also aids in the design of cobalt porphyrin‐based catalysts for CO_2_RR.

## Results and Discussion

**Figure 1:**
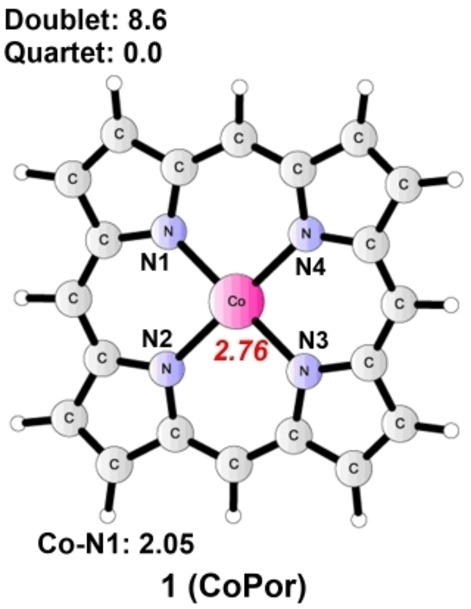
Optimized structure of **1** (total charge of 0). Energies relative to the ground state are given in kcal mol^−1^. Bond distance is given in Ångström. The spin population on Co is shown in red italics.

**Figure 2:**
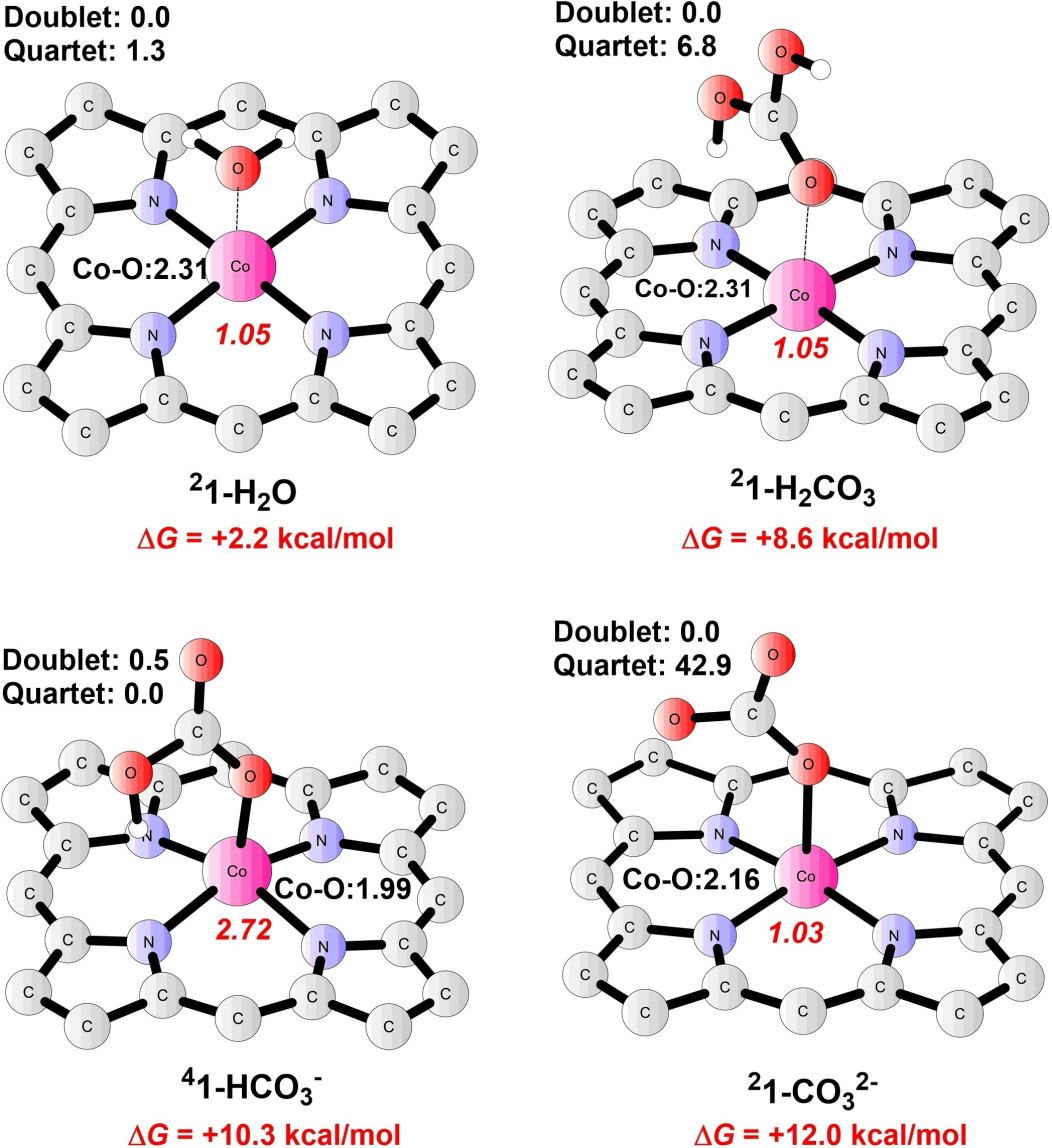
Optimized structures of **1‐H_2_O** (total charge of 0), **1‐H_2_CO_3_** (total charge of 0), **1‐HCO_3_** ^**−**^ (total charge of −1), and **1‐CO_3_** ^**2−**^ (total charge of −2). Optimizations were implemented in the gas phase, and the final energies were calculated in the aqueous solution. Energies relative to the ground states and the reaction Gibbs free energy change (Δ*G*) for the generation of each adduct are given in kcal mol^−1^. Bond distances are given in Ångström. The spin populations on Co are given in red italics. Unimportant H atoms are omitted.

As shown in Figure 1, the model catalyst **CoPor** is labeled as **1**, which has a ground state of a quartet, and the doublet state is calculated to be 8.6 kcal mol^−1^ higher in energy. Mulliken spin population analysis of ^4^ **1** (Co^II^−L) revealed a spin population of 2.76 on Co. The distances between Co and the four coordinated N atoms are 2.05 Å on average. To determine the lowest energy starting point of the energy profile, the formation of various adducts, including **1‐H_2_O**, **1‐H_2_CO_3,_ 1‐HCO_3_** ^**−**^, and **1‐CO_3_** ^**2−**^, have been considered. The corresponding calculated free energies relative to **1** plus each species (**H_2_O**, **H_2_CO_3_**, **HCO_3_** ^**−**^, and **CO_3_** ^**2−**^, respectively) are displayed in Figure 2. Obviously, the axial coordination to catalyst **1** is all endergonic albeit to a different extent (**1‐H_2_O**: 2.2 kcal mol^−1^; **1‐H_2_CO_3_**: 8.6 kcal mol^−1^_,_ **1‐HCO_3_** ^**−**^: 10.3 kcal mol^−1^; **1‐CO_3_** ^**2−**^: 12.0 kcal mol^−1^). Therefore, catalyst **1** was chosen as the starting point in the energy profiles displayed in Figures 4–5.

**Reduction of CO_2_ to CO**. To reach the active state towards attacking CO_2_, complex **1** is firstly reduced via a one‐electron‐reduction step to form intermediate **2**, with a calculated potential of −1.26 V; hence, this step is endergonic by 3.8 kcal mol^−1^ given an applied potential of −1.10 V. The ground state of **2** (Co^II^−L⋅^−^) is a triplet, whereas the quintet, the broken‐symmetry singlet, and the closed‐shell singlet are 2.5, 5.5, and 11.1 kcal mol^−1^ higher in energy, respectively. At ^**3**^ **2**, the Mulliken spin population is 2.68 on Co (*S*_Co_=3/2) and −0.68 on the ligand (S_L_=1/2), suggesting an antiferromagnetically interacting manner between the Co center and the ligand radical anion. Besides, at pH=7.3, the direct protonation of the metal coordinated N atom of **1** to form **1 pt**, the p*K*_a_ of which is calculated to be −3.7 (Figure S1), is found to be endergonic by 15.0 kcal mol^−1^.

**Figure 3:**
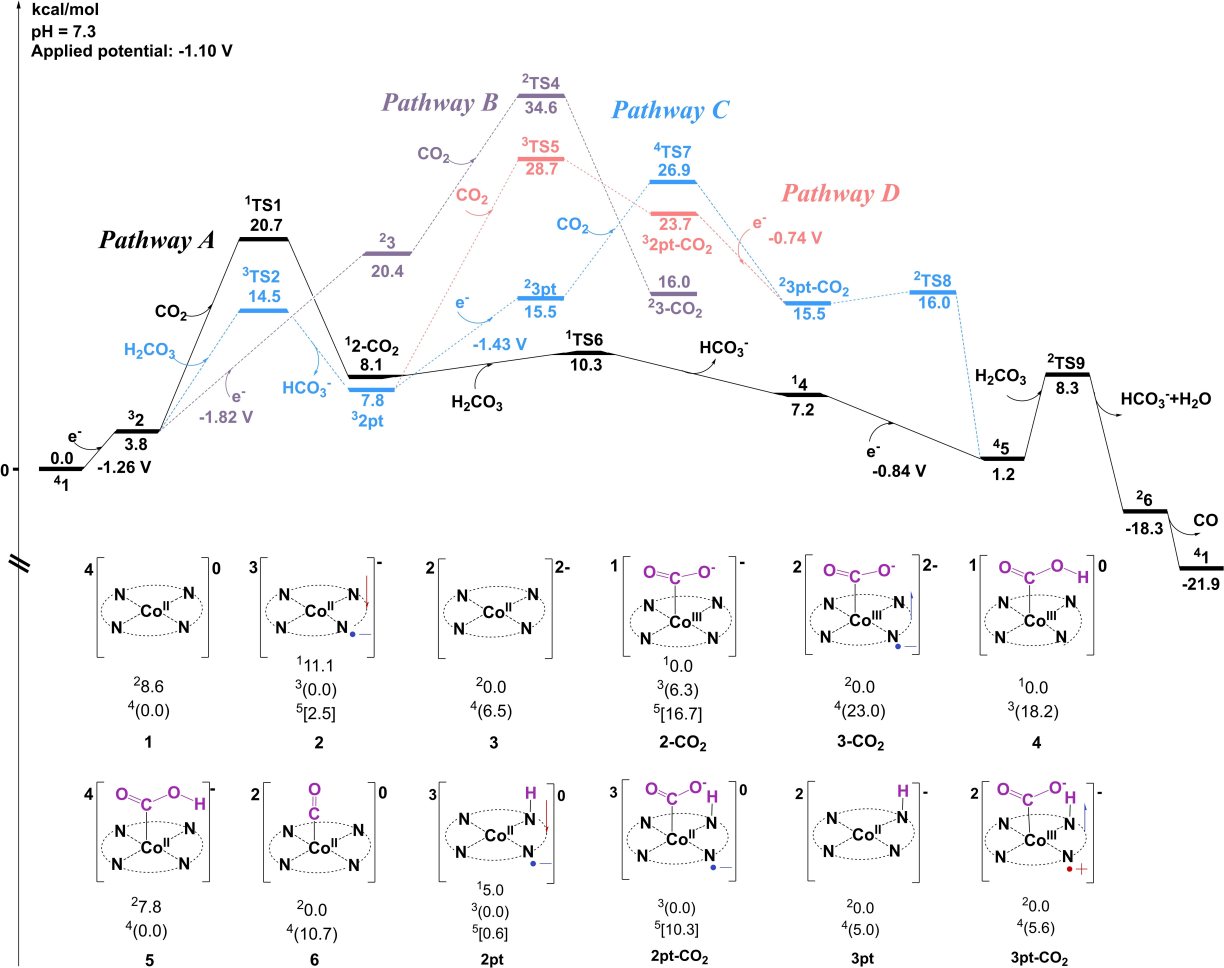
Gibbs energy diagram for the reduction of CO_2_ to CO. The energies are given in kcal mol^−1^. An applied potential of −1.10 V and pH of 7.3 are used as the reference. Core structures of intermediates are given; the ground spin state and total charge are shown within. Relative energies are shown for low‐, intermediate‐, and high‐spin states (in parentheses).

**Figure 4:**
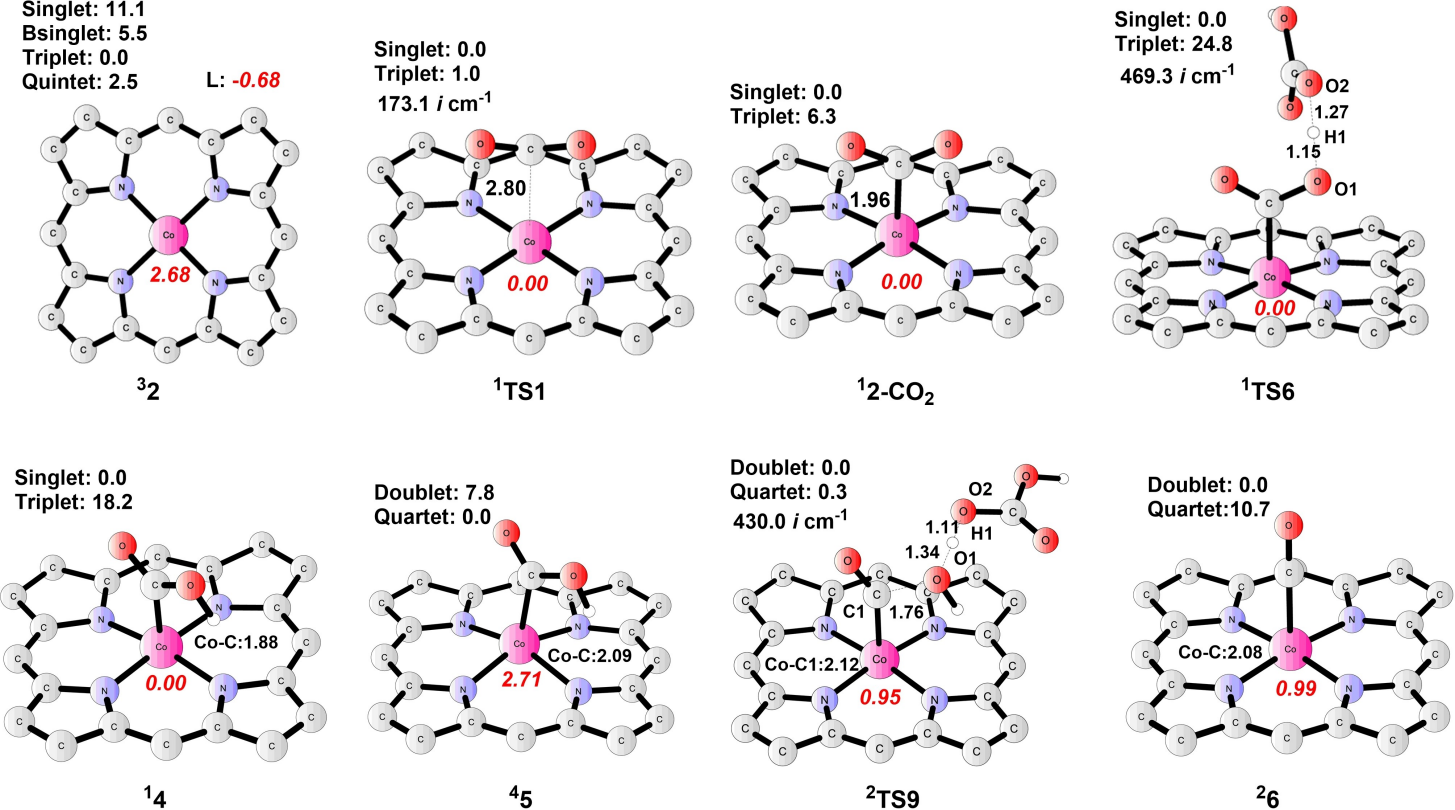
Optimized structures of important intermediates and transition states in CO_2_RR catalysis. Energies relative to the ground states are given in kcal mol^−1^, and imaginary frequencies (*i* cm^−1^) for transition states are shown. Bond distances are given in Ångström. The spin populations on Co and ligand are given in red italics. Unimportant H atoms are omitted.

Starting from **2**, various pathways have been considered for the CO_2_RR to produce CO. As shown in pathway A (Figure 3), **2** (Co^II^−L⋅^−^) could perform a nucleophilic attack of **CO_2_** via **TS1** to generate **2‐CO_2_**, endergonic by 4.3 kcal mol^−1^. The ground state of **TS1** (Figure 4) is a mixture of the closed‐shell singlet and the triplet since the latter is only 1.0 kcal mol^−1^ higher in energy than the former. The imaginary frequency of **TS1** in the singlet state is 173.1*i* cm^−1^, where the vibration mode is related to the Co−C bond formation (the distance of Co−C is 2.80 Å). The calculated total barrier of ^**1**^ **TS1** is 20.7 kcal mol^−1^ relative to **1**. Complex **2‐CO_2_** has a ground state of the closed‐shell singlet; the triplet is 6.3 kcal mol^−1^ higher in energy. Therefore, a spin crossing must occur in the transformation of **2** to **2‐CO_2_**.

Afterward, **2‐CO_2_** could abstract a proton from H_2_CO_3_ to generate complex **4** (Co^III^−L−COOH), which lies at −0.9 kcal mol^−1^ relative to **2‐CO_2_**. The corresponding protonation transition state **TS6** (Figure 4) has a ground state of a singlet, and the triplet is 24.8 kcal mol^−1^ higher in energy. **TS6** has been identified to be associated with a single imaginary frequency of 469.3*i* cm^−1^, the vibration mode of which is related to O1−H1 bond formation (the distances of O1−H1 and O2−H1 are 1.15 Å and 1.27 Å, respectively). The total barrier of **TS6** is only 10.3 kcal mol^−1^, indicating a facile process. Next, a one‐electron reduction on the Co center of **4** (Co^III^−L−COOH) leads to the generation of **5** (Co^II^−L−COOH). Complex **5** prefers to be a quartet, with a Mulliken spin population of 2.71 on Co, while the doublet is 7.8 kcal mol^−1^ higher. In this reduction step, the Co−C bond distance elongates from 1.88 Å in **4** to 2.09 Å in **5**.

Noteworthy, the nucleophilic attack of **CO_2_** by **3**, which is formed by the reduction of **2**, was also calculated, and the results are displayed as pathway B (Figure 3). Calculations revealed that the ground state of **3** is a doublet (with the Mulliken spin population of 0.89 on Co), and the quartet is 6.5 kcal mol^−1^ higher. The **2**/**3** transition has a potential of −1.82 V, thereby rendering this reduction process endergonic by 16.6 kcal mol^−1^. Followingly, the cobalt center of **3** performs a nucleophilic attack on CO_2_ to generate **3‐CO_2_** via **TS4**. **3‐CO_2_** (Figure S2) has a doublet ground spin state. The total barrier of **TS4** (ground state of doublet, see Figure S2), however, is calculated to be as high as 34.6 kcal mol^−1^, thus, such a reaction manner is ruled out.

**Figure 5:**
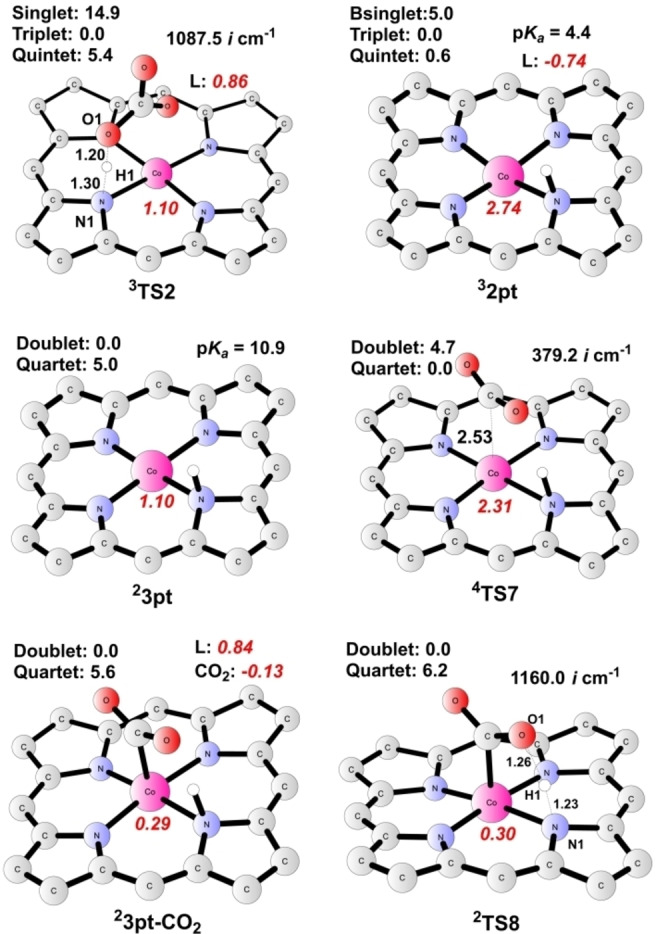
Optimized structures of important intermediates and transition states in CO_2_RR catalysis. Energies relative to the ground states are given in kcal mol^−1^, and imaginary frequencies (*i* cm^−1^) for transition states are shown. Bond distances are given in Ångström. The spin populations on Co and ligand are given in red italics. Unimportant H atoms are omitted.

Additionally, the protonation of **2** prior to the upcoming reaction to produce complex **5** has also been investigated,^[16]^ as seen for pathway C (Figure 3). The protonation of **2** via **TS2** results in the generation of **2 pt** (Co^II^−L⋅^−^−H, Figure 5). **TS2** has a triplet ground state, where the spin populations on Co and the ligand are 1.10 and 0.86, respectively. The closed‐shell singlet and the open‐shell quintet are 14.9 kcal mol^−1^ and 5.4 kcal mol^−1^ higher than the triplet. Frequency analysis of **TS2** indicates a sole imaginary frequency of 1087.5*i* cm^−11^, the vibration mode of which is related to N−H formation. As for **2 pt**, calculations suggest a ground state of a mixture of triplet and quintet since the energy difference between them is only 0.6 kcal mol^−1^. This protonation step (^**3**^ **2**→^**3**^ **2 pt**) is thus calculated to be endergonic by 4.0 kcal mol^−1^.

A further reduction of **2 pt** affords intermediate **3 pt** (Figure 5), with a redox potential of −1.43 V; the ground state of **3 pt** is a doublet (spin population of 1.10 on Co), whereas the quartet lies at +5.0 kcal mol^−1^ higher. The following attack on CO_2_ by **3 pt** produces **3 pt‐CO_2_** (Co^III^−L⋅^+^−CO_2_). The associated transition state **TS7** (Figure 5) is a quartet (Mulliken spin population is 2.31 on Co), and the doublet is 4.7 kcal mol^−1^ higher. ^**4**^ **TS7** has a single imaginary frequency of 379.2*i* cm^−1^, and its vibration mode is related to the Co−C bond formation. The resulting species **3 pt‐CO_2_** prefers to be a doublet, with a spin population of 0.29 on Co and 0.84 on the ligand, indicating that the metal center ferromagnetically interacts with the porphyrin moiety. Calculations suggest a total energy barrier of 26.9 kcal mol^−1^ for **TS7**. Subsequently, a nearly barrierless intramolecular proton transfer occurs in **3 pt‐CO_2_** via **TS8** (Figure 5), yielding intermediate **5** (Co^II^−L−COOH). During this process, the proton on the metal‐coordinated N atom transfers to the CO_2_ moiety, generating the carboxyl group. Alternatively, the direct nucleophilic attack on CO_2_ by **2 pt** via **TS5** to give complex **2 pt‐CO_2_** (pathway D) has a total barrier of 28.7 kcal mol^−1^, even higher than that of **TS7** in pathway C.

Taken together, for the transformation of **2** to **5**, the total barrier for pathway C (**2→2 pt→3 pt→3 pt‐CO_2_→5**) is more than 6 kcal mol^−1^ higher than that of pathway A (**2→2‐CO_2_→4→5**), thus excluding the possibility of CO_2_RR taking place at the protonated state of **2**, namely, **2 pt**. In addition, the critical CO_2_ addition step preferably takes place at a formal Co(I) center as seen for complex **2** in pathway A.

Thereafter, protonation of **5** on its COOH group by an H_2_CO_3_ molecule results in the heterolytic cleavage of the C−O bond to produce intermediate **6** (Co^II^−L−CO), accompanied by the release of H_2_O and HCO_3_^−^. The related transition state **TS9** has a ground state featuring a mixture of the doublet and the quartet state, where the energy difference is only 0.3 kcal mol^−1^ between both. **TS9** has a single imaginary frequency of 430.0*i* cm^−1^, the vibration mode of which is consistent with the C1−O1 bond cleavage as well as the H1−O1 bond formation. The distances of C1−O1, O1−H1, and H1−O2 are 1.76 Å, 1.34 Å, and 1.11 Å, respectively. The formation of complex **6** (doublet ground state) from **5** is calculated to be exergonic by 19.5 kcal mol^−1^. Eventually, the catalytic cycle closes as CO dissociates from the cobalt center of **6** (Co^II^−L−CO) to regenerate **1**, and this step is calculated to be exergonic by 3.6 kcal mol^−1^.

Above all, the rate determining step (RDS) for CO_2_RR via pathway A is the nucleophilic attack of **2** on CO_2_, with a total energy barrier of 20.7 kcal mol^−1^ (see **TS1** in Figure 4).

**Figure 6:**
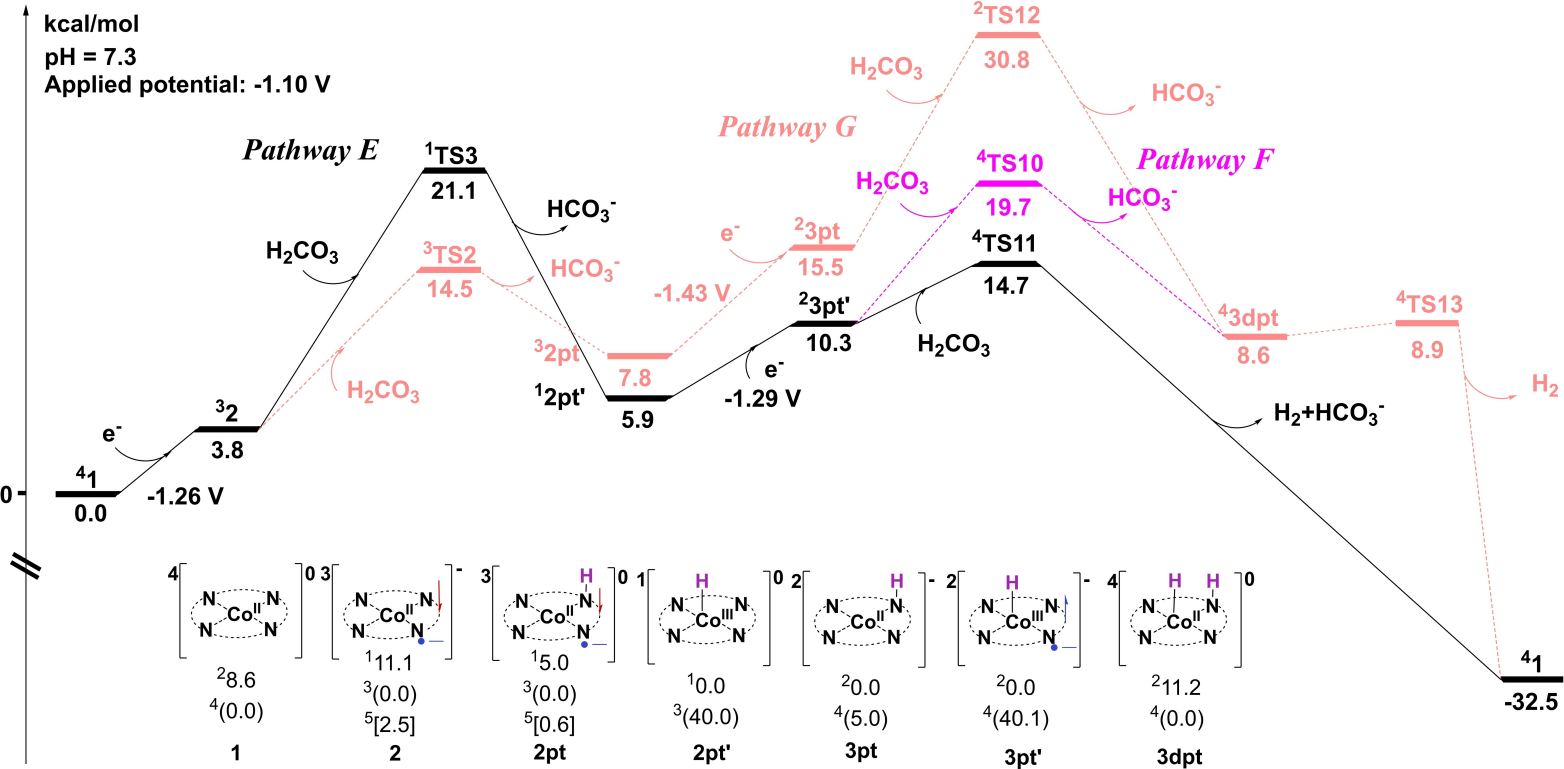
Gibbs energy diagram for the release of H_2_. The energies are given in kcal mol^−1^. An applied potential of −1.10 V and pH of 7.3 are used as the reference. Core structures of intermediates are given; the ground spin state and total charge are shown within. Relative energies are shown for low‐, intermediate‐, and high‐spin states (in parentheses).

**Figure 7:**
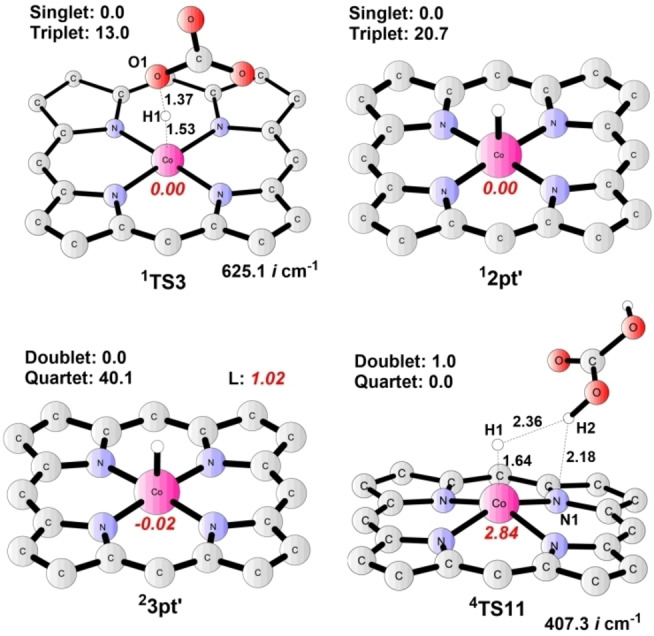
Optimized structures of important intermediates and transition states for HER. Energies relative to the ground states are given in kcal mol^−1^, and imaginary frequencies (*i* cm^−1^) for transition states are shown. Bond distances are given in Ångström. The spin populations on Co and ligand are given in red italics. Unimportant H atoms are omitted.

**Reduction of H^+^ to H_2_**. As the main competing reaction to the CO_2_RR, possible pathways for the hydrogen evolution reaction have also been calculated. The results are provided in Figure 6 (selected optimized structures are shown in Figure 7). In pathway E (Figure 6), the Co‐hydride species **2 pt’** is formed by the protonation of **2** via **TS3**, with a barrier of 17.3 kcal mol^−1^ relative to **2** plus H_2_CO_3_. **TS3** has a singlet ground state (the triplet lies at +13.0 kcal mol^−1^ relative to the singlet); frequency analysis of ^**1**^ **TS3** (Figure 7) suggests a single imaginary frequency of 625.1*i* cm^−1^, associated with a vibration mode of the Co−H bond formation. The resulting intermediate **2 pt’** (Co^III^−H−L) is also a singlet, lying at +2.1 kcal mol^−1^ relative to **2**. Spin population analysis revealed that the formation of **2 pt’** is accompanied by two electrons, one from the metal center and the other from the porphyrin ligand, transferred from the catalyst to the hydrogen.

A further one‐electron reduction of the porphyrin ligand in **2 pt’** gives complex **3 pt’** (Figure 7), with a calculated potential of −1.29 V. **3 pt’** (Co^III^−H−L⋅^−^), with a doublet ground state, lies at +4.4 kcal mol^−1^ above **2 pt’**. Next, the Co center of **3 pt’** accepts a proton from H_2_CO_3_ via **TS11** (Figure 7) to produce H_2_, and regenerates species **1** to participate in the next cycle. Calculations indicate that **TS11** is a mixture of the quartet and the doublet due to the small energy difference (1.0 kcal mol^−1^) between the two states. The energy barrier of **TS11** is 8.8 kcal mol^−1^ relative to **2 pt’**, and the total reaction free energy for the HER is −32.5 kcal mol^−1^. Besides, the metal‐coordinated N atom in **3 pt’** could be protonated by another H_2_CO_3_ molecule to yield species **3 dpt** via **TS10** (pathway F in Figure 6), associated with an energy barrier of 5.0 kcal mol^−1^ higher than that of **TS11**. Next, **3 dpt** undergoes an H−H coupling step (via **TS13**) to release H_2_ and regenerate species **1**. Apparently, the reaction manner in pathway F is less favored compared to pathway E.

In parallel (pathway G in Figure 6), intermediate **2** could first get protonated on its N site via **TS2**, with a total barrier of 14.5 kcal mol^−1^, to form complex **2 pt**. **2 pt** is only 1.9 kcal mol^−1^ higher in energy than **2 pt’**. Nevertheless, the reduction of **2 pt** to **3 pt** (redox potential of −1.43 V), and the following protonation step (via **TS12**, total barrier of 30.8 kcal mol^−1^) are disfavored compared to that in pathway E; thus, pathway G is ruled out in this HER. Additionally, the transformation from **2 pt** to **2 pt’** was found to be kinetically disfavored as well (for details, see Figure S4).

Based on the above analysis, the RDS for this HER is the transformation of **2** to **2 pt’**, with a total energy barrier of 21.1 kcal mol^−1^ (see **TS3**), slightly higher than that of CO_2_RR (**TS1**: 20.7 kcal mol^−1^).

**The substitution effects**. To explore the effects of substituents on the reactivity and the selectivity of the CO_2_RR, catalysts equipped with different substituents (as shown in Scheme 2b) have been computationally studied. The corresponding energy profiles for the reduction of CO_2_RR to CO via pathway A (the preferred reaction pathway based on the calculated results of the model reaction) are displayed in Figure S10. The metal‐hydride formation step was also calculated as it is the RDS for the HER. For convenience, the calculated results of critical steps are summarized in Table 1.

**Table 1:** Comparison of the redox potentials, reaction free energies (Δ*G*), and the energy barriers between CO_2_RR and HER. Δ*G* ^ǂ^_CO2RR_ and Δ*G* ^ǂ^_HER_ represents the total energy barriers of CO_2_RR and HER, ΔΔ*G* ^**ǂ**^ **=**Δ*G* ^ǂ^_CO2RR_−Δ*G* ^ǂ^_HER_.

Catalyst	**1→2** [V]	**2→2‐CO_2_** [kcal mol^−1^]			**2→2 pt’** [kcal mol^−1^]			ΔΔ*G* ^≠^ [kcal mol^−1^] (selectivity)
		Δ*G*	Δ*G* ^≠^	Δ*G* ^≠^_CO2RR_	Δ*G*	Δ*G* ^≠^	Δ*G* ^≠^_HER_	
**CoPor**	−1.26	+4.3	+16.9	+20.7	+2.1	+17.3	+21.1	−0.4 (66 %)
**CoPor−R1**	−1.26	+4.5	+16.9	+20.5	+4.9	+17.1	+20.7	−0.2 (58 %)
**CoPor−R2**	−1.30	+3.0	+17.0	+21.7	+3.6	+18.3	+23.0	−1.3 (90 %)
**CoPor−R3**	−1.23	+3.3	+16.3	+19.3	+5.1	+19.0	+22.0	−2.7 (98 %)
**CoPor−R4**	−1.28	+4.2	+17.7	+21.9	+3.8	+18.8	+23.0	−1.1 (87 %)

As shown in Table 1, the redox potentials for the reduction of **1** to **2** barely change when modifying the catalyst with different substituents. The catalytic performances of different catalysts are also quite similar, favoring the production of CO over H_2_. Meanwhile, the energy difference (ΔΔ*G* ^≠^) between CO_2_RR and HER by using catalysts **CoPor−R2** (**Co(TAP)**, see above), **CoPor−R3**, and **CoPor−R4** could lead to more than 85 % of selectivity toward CO over H_2_ production; the trend of these findings is in line with the experimental observations that CO is the major product when using **Co(TAP)** and COFs. Especially, catalyst **CoPor−R3** shows the highest selectivity toward CO_2_RR over HER, since the total energy barrier of CO_2_RR is 2.7 kcal mol^−1^ lower than that of HER, corresponding to around 98 % of CO selectivity. The total barrier for the CO_2_RR to CO (**CoPor−R3**: 19.3 kcal mol^−1^) is also the lowest one among them. Therefore, **CoPor−R3** is likely to show a high performance both in the activity and the CO selectivity when applied as a molecular cobalt porphyrin catalyst in a homogeneous aqueous solution.

## Conclusion

**Scheme 3:**
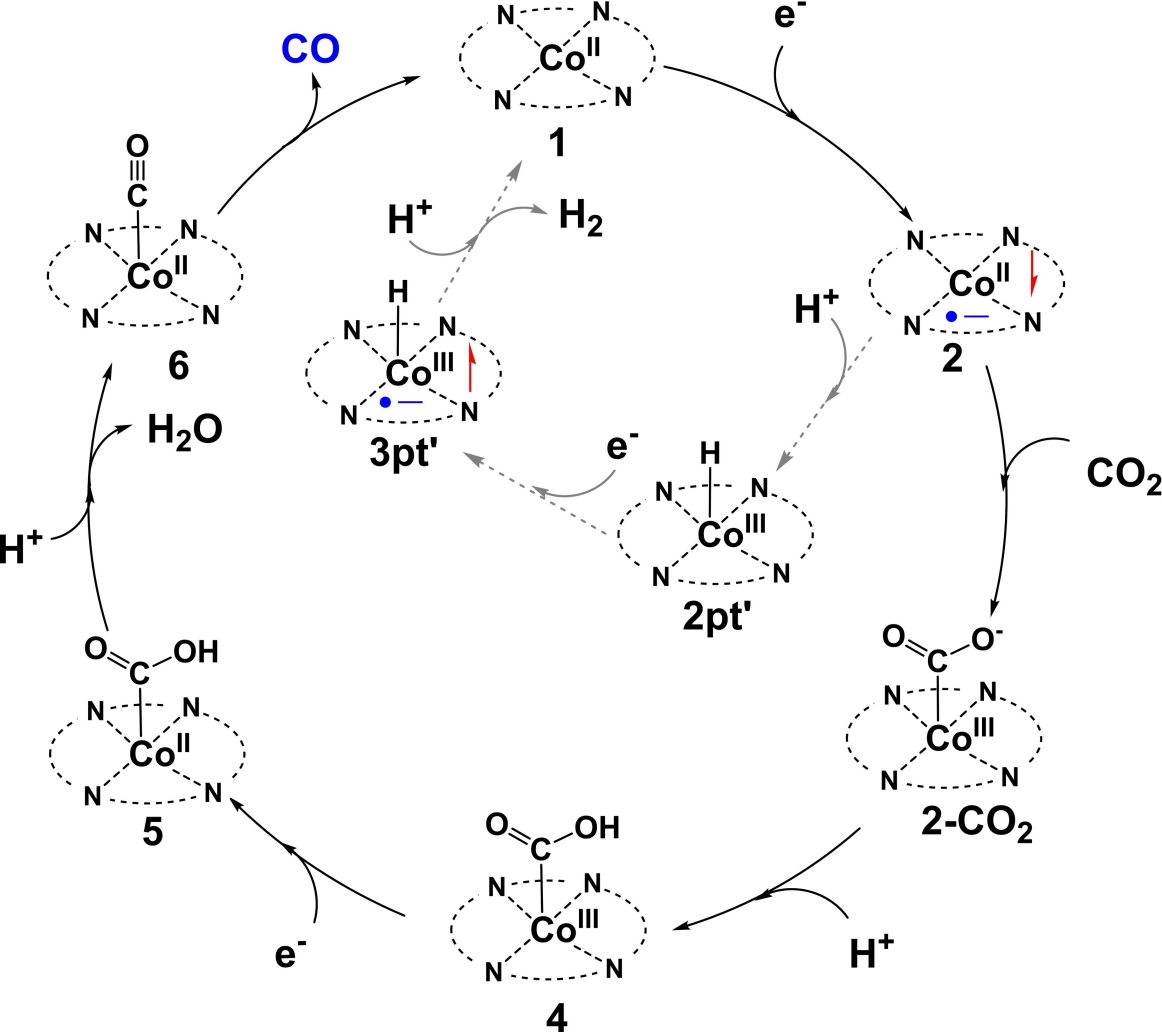
Mechanism of CO_2_RR to CO catalyzed by cobalt‐porphyrin catalyst.

In this work, detailed mechanistic investigations on the CO_2_RR to CO by **CoPor** (**1**) in neutral aqueous solutions were conducted using DFT calculations. Possible reaction pathways involving critical intermediates and transition state structures were considered. On the basis of the calculated results, a catalytic cycle has been established, as shown in Scheme 3. Initially, the Co^II^‐porphyrin catalyst **1** undergoes a ligand‐based one‐electron‐reduction step to form intermediate **2** (Co^II^−L⋅^−^), which then performs a nucleophilic attack on CO_2_ to generate the critical **2‐CO_2_** species. Protonation of **2‐CO_2_** by H_2_CO_3_ affords complex **4** (Co^III^−L−COOH), followed by a reduction step to give **5** (Co^II^−L−COOH). A proton transfer from H_2_CO_3_ to the COOH group of **5** results in the heterolytic cleavage of the C−O bond to produce intermediate **6** (Co^II^−L−CO), accompanied by the release of an H_2_O molecule. Finally, CO dissociates from the cobalt center of **6** (Co^II^−L−CO) to regenerate **1** to catalyze the next cycle. Calculations indicate that the RDS of this CO_2_RR is the nucleophilic attack on CO_2_ by intermediate **2** (Co^II^−L⋅^−^), with a total energy barrier of 20.7 kcal mol^−1^. Meanwhile, the mechanism of the competing HER was also studied and the RDS of which is the formation of the metal hydride, as seen **TS3** in Figure 7, with a total barrier of 21.1 kcal mol^−1^.

Furthermore, a comparison of the total energy barriers of the CO_2_RR and HER catalyzed with a series of modified Co^II^‐porphyrin catalysts was conducted (Figure S10 and Table 1). The redox potentials for the reduction of **1** to **2** remain almost unchanged when tuning the substituents. The catalytic performances of different catalysts are in the same trend that favors the production of CO over H_2_. Hereto, the introduction of the redox non‐innocent porphyrin ligand could possibly help to circumvent the formation of a low‐valent Co center that leads to the formation of the metal‐hydride, thus contributing to the CO_2_RR to produce CO. Importantly, among all the catalysts considered, **CoPor−R3** is likely to show a higher catalytic capability, both the activity and the CO selectivity, than the rest ones when applied as a molecular cobalt porphyrin catalyst in homogeneous aqueous solution.

## Computational Methods

All DFT calculations presented herein were conducted using the B3LYP‐D3 functional^[17]^ (the default Grimme‐D3 dispersion correction) together with the SMD continuum solvation model,^[18]^ as implemented in the Gaussian 16 program package.^[19]^ As for the geometry optimization, the 6‐31G(*d*,*p*) basis sets were used for C, N, O, and H elements, and the SDD pseudopotential was used for Co.^[20]^ To verify the features of intermediates and transition states and to obtain the Gibbs free energy corrections at 298.15 K, frequency analysis was implemented at the same level as the geometry optimization. On the basis of optimized geometries, more accurate electronic energies were obtained by single‐point calculations employing the larger basis set 6‐311+G(*2df*,*2p*) for C, N, O, and H (Co SDD). Spin population analysis was conducted using Multiwfn.^[21]^

The redox potentials were calculated using the standard hydrogen electrode (SHE, experimental value of 4.28 V) as the reference,^[22]^ corresponding to an electron affinity of 98.72 kcal mol^−1^. The applied potential in the titled reaction is −1.10 V vs. SHE. For a proton, the Gibbs free energy in the gas phase is −6.3 kcal mol^−1^, and the experimental absolute solvation energy for a proton is −264.0 kcal mol^−1^ in water.^[22]^ Considering the experimental pH of 7.3 in the buffer, the total Gibbs free energy of a proton is −280.3 kcal mol^−1^.

Additionally, a concentration correction of 1.9 kcal mol^−1^ at 298.15 K, derived from the Gibbs free energy change from 1 atm (24.5 L mol^−1^ for an ideal gas) to 1 m (1 mol L^−1^ in solution), was also added to all species except for water and carbonic acid. For water as solvent (standard concentration of 55.56 mol L^−1^), the correction is 4.3 kcal mol^−1^; for carbonic acid, the concentration correction is −3.9 kcal mol^−1^.^[23]^

## Conflict of interest

There are no conflicts to declare.

1

## Supporting information

As a service to our authors and readers, this journal provides supporting information supplied by the authors. Such materials are peer reviewed and may be re‐organized for online delivery, but are not copy‐edited or typeset. Technical support issues arising from supporting information (other than missing files) should be addressed to the authors.

Supporting Information

## Data Availability

The data that support the findings of this study are available in the supplementary material of this article.
