# Supplementary material for: Deciphering the Selectivity of the Electrochemical CO2 Reduction to CO by a Cobalt Porphyrin Catalyst in Neutral Aqueous Solution: Insights from DFT Calculations
Source: ChemistryOpen. 2023 Feb 6;12(2):e202200254. doi: 10.1002/open.202200254 (PMC9900731; doi:10.1002/open.202200254)
Supplement: Supplementary file 1 — Supporting Information [file OPEN-12-e202200254-s001.pdf]

ChemistryOpen

Supporting Information

# Deciphering the Selectivity of the Electrochemical $CO_2$ Reduction to CO by a Cobalt Porphyrin Catalyst in Neutral Aqueous Solution: Insights from DFT Calculations

Yu-Chen Cao, Le-Le Shi, Man Li,* Bo You, and Rong-Zhen Liao*

## Contents

Section 1. Tables, Figures, and Schemes S2

Section 2. Energies for all stationary points S13

Section 3. Cartesian Coordinates of Optimized Structures S15

## Section 1. Tables, Figures, and Schemes

**Table S1.** Calculated Mulliken spin populations on critical atoms or fragments of various stationary points.

| Complex | Multiplicity | Spin population | | |
|---|---|---|---|---|
| | | Co | Por | $CO_2$(CO) |
| **1** | 2 | 1.05 | -0.05 | / |
| | 4 | 2.76 | 0.24 | / |
| **1-$H_2O$** | 2 | 1.05 | -0.08 | / |
| | 4 | 2.76 | 0.21 | / |
| **1-$H_2CO_3$** | 2 | 1.05 | -0.07 | 0.02 |
| | 4 | 2.73 | 0.23 | 0.04 |
| **1-$HCO_3^-$** | 2 | 1.00 | -0.07 | 0.07 |
| | 4 | 2.72 | 0.18 | 0.10 |
| **1-$CO_3^{2-}$** | 2 | 1.03 | 0.81 | -0.84 |
| | 4 | 1.03 | 0.93 | 1.04 |
| **2** | 1 | 0.00 | 0.00 | / |
| | 3 | 2.66 | -0.66 | / |
| | 5 | 2.75 | 1.25 | / |
| **2pt** | 1 | 1.07 | -1.07 | / |
| | 3 | 2.74 | -0.74 | / |
| | 5 | 2.77 | 1.23 | / |
| **2pt'** | 1 | 0.00 | 0.00 | / |
| | 3 | 2.29 | 0.02 | / |
| **2-$CO_2$** | 1 | 0.00 | 0.00 | 0.00 |
| | 3 | 2.37 | 0.15 | -0.52 |
| | 5 | 2.98 | 0.30 | 0.72 |
| **2pt-$CO_2$** | 3 | 2.38 | 0.14 | -0.52 |
| | 5 | 2.93 | 0.24 | 0.83 |
| **3** | 2 | 0.89 | 0.11 | / |
| | 4 | 1.08 | 1.92 | / |
| **3pt** | 2 | 1.10 | -0.10 | / |
| | 4 | 1.16 | 1.84 | / |
| **3pt'** | 2 | -0.02 | 1.02 | / |
| | 4 | 0.05 | 2.98 | / |
| **3dpt** | 2 | 0.19 | 0.84 | / |
| | 4 | 2.80 | 0.10 | / |
| **3-$CO_2$** | 2 | 0.00 | 1.00 | 0.00 |
| | 4 | 1.05 | 1.95 | 0.00 |
| **3pt-$CO_2$** | 2 | 0.29 | 0.84 | -0.13 |
| | 4 | 2.36 | 1.15 | -0.51 |

| Complex | Multiplicity | Spin population | | |
|---|---|---|---|---|
| | | Co | Por | $CO_2$(CO) |
| **4** | 1 | 0.00 | 0.00 | 0.00 |
| | 3 | 2.37 | 0.06 | -0.43 |
| **5** | 2 | -0.03 | 1.02 | 0.01 |
| | 4 | 2.71 | 0.21 | 0.08 |
| **6** | 2 | 0.99 | -0.06 | 0.07 |
| | 4 | 2.71 | 0.26 | 0.03 |
| **TS1** | 1 | 0.00 | 0.00 | 0.00 |
| | 3 | 2.38 | 0.14 | -0.52 |
| **TS2** | 1 | 0.00 | 0.00 | / |
| | 3 | 1.10 | 0.86 | / |
| | 5 | 2.74 | 1.21 | / |
| **TS3** | 1 | 0.00 | 0.00 | / |
| | 3 | 2.21 | 0.11 | / |
| **TS4** | 2 | 0.13 | 0.88 | -0.01 |
| | 4 | 1.27 | 0.97 | 0.76 |
| **TS5** | 1 | 0.00 | 0.00 | 0.00 |
| | 3 | 2.35 | -0.12 | -0.23 |
| **TS6** | 1 | 0.00 | 0.00 | 0.00 |
| | 3 | 2.19 | 0.12 | -0.31 |
| **TS7** | 2 | 0.14 | 0.86 | 0.00 |
| | 4 | 2.31 | 0.91 | -0.22 |
| **TS8** | 2 | 0.30 | 0.71 | -0.01 |
| | 4 | 2.66 | 0.20 | 0.14 |
| **TS9** | 2 | 0.95 | -0.05 | 0.10 |
| | 4 | 2.71 | 0.22 | 0.07 |
| **TS10** | 2 | 0.10 | 0.91 | / |
| | 4 | 2.81 | 0.18 | / |
| **TS11** | 2 | -0.01 | 1.01 | / |
| | 4 | 2.72 | 0.16 | / |
| **TS12** | 2 | 0.06 | 0.94 | / |
| | 4 | 2.20 | 1.10 | / |
| **TS13** | 2 | 0.94 | 0.03 | / |
| | 4 | 2.66 | 0.23 | / |
| **TS14** | 1 | 0.00 | 0.00 | / |
| | 3 | 2.20 | 0.02 | / |

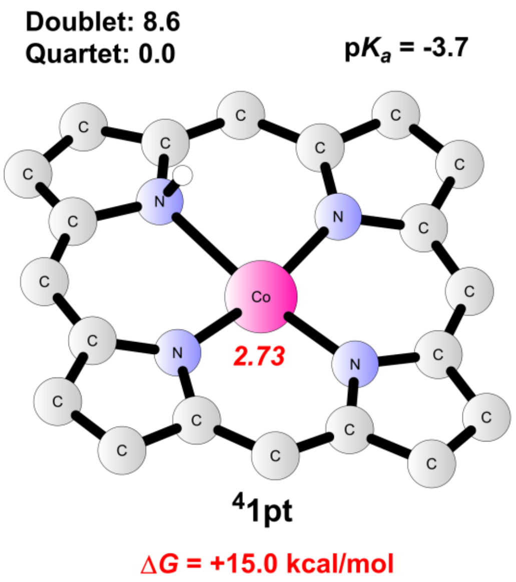


**Figure S1.** Optimized structure of intermediate **1pt** (total charge of 1). Optimization was implemented in the gas phase, and the final energies were calculated in the aqueous solution. Energies relative to the ground state and Gibbs free energy change ($\Delta G$) for the generation of **1pt** from **1** are given in kcal mol$^{-1}$. The spin populations on Co are given in red italics. Unimportant H atoms are omitted.

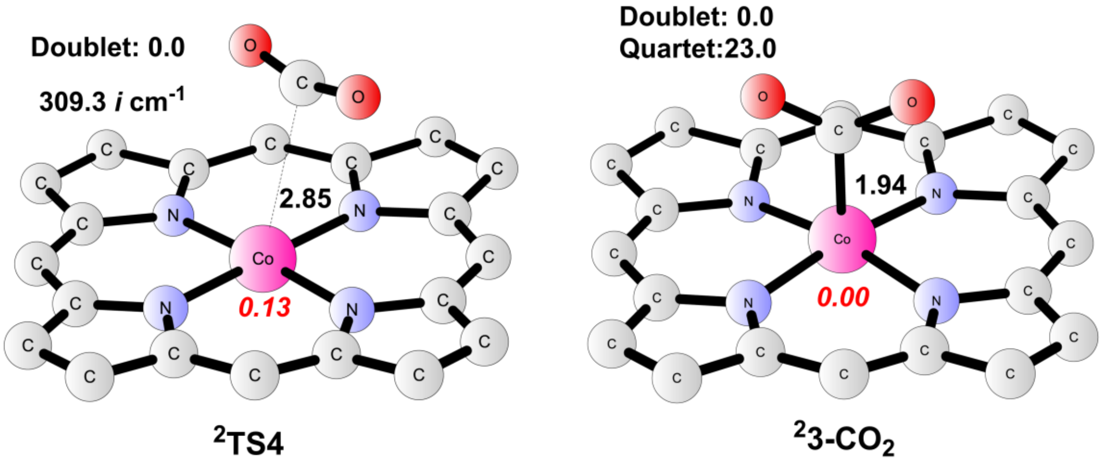


**Figure S2.** Optimized structures of transition state **TS4** (total charge of -2) and intermediate **3-CO$_2$** (total charge of -2). Energies relative to the ground states are given in kcal mol$^{-1}$, and imaginary frequency ($i$ cm$^{-1}$) for **TS4** is shown. Bond distances are given in Ångström. The spin populations on Co are given in red italics. Unimportant H atoms are omitted.

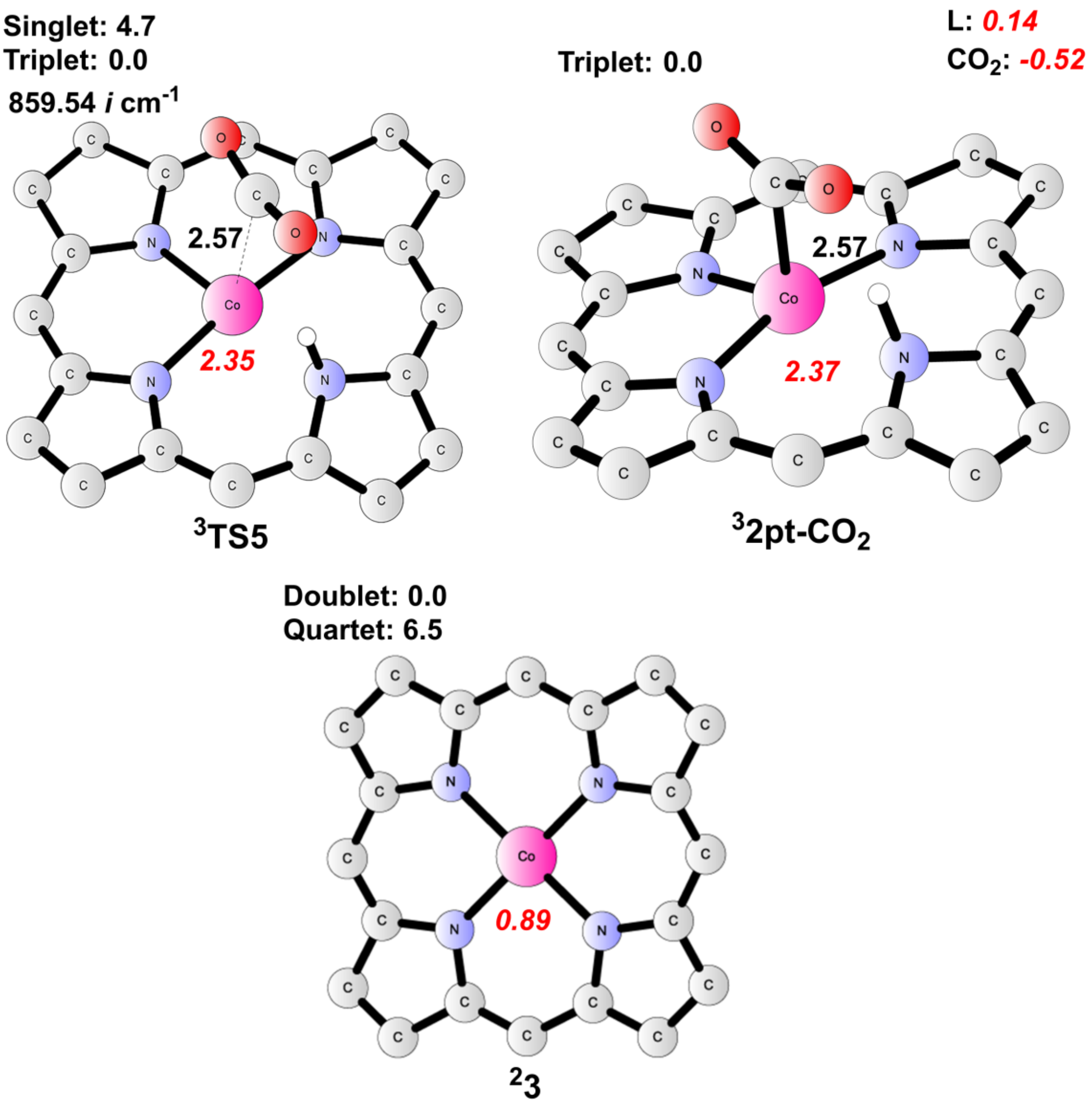


**Figure S3.** Optimized structures of transition state **TS5** (total charge of 0) and intermediates **2pt-CO$_2$** (total charge of 0), $^2$**3** (total charge of -2). Energies relative to the ground states are given in kcal mol$^{-1}$, and imaginary frequency ($i$ cm$^{-1}$) for **TS5** is shown. Bond distances are given in Ångström. The spin populations on Co, ligands, and CO$_2$ are in red italics. Unimportant H atoms are omitted.

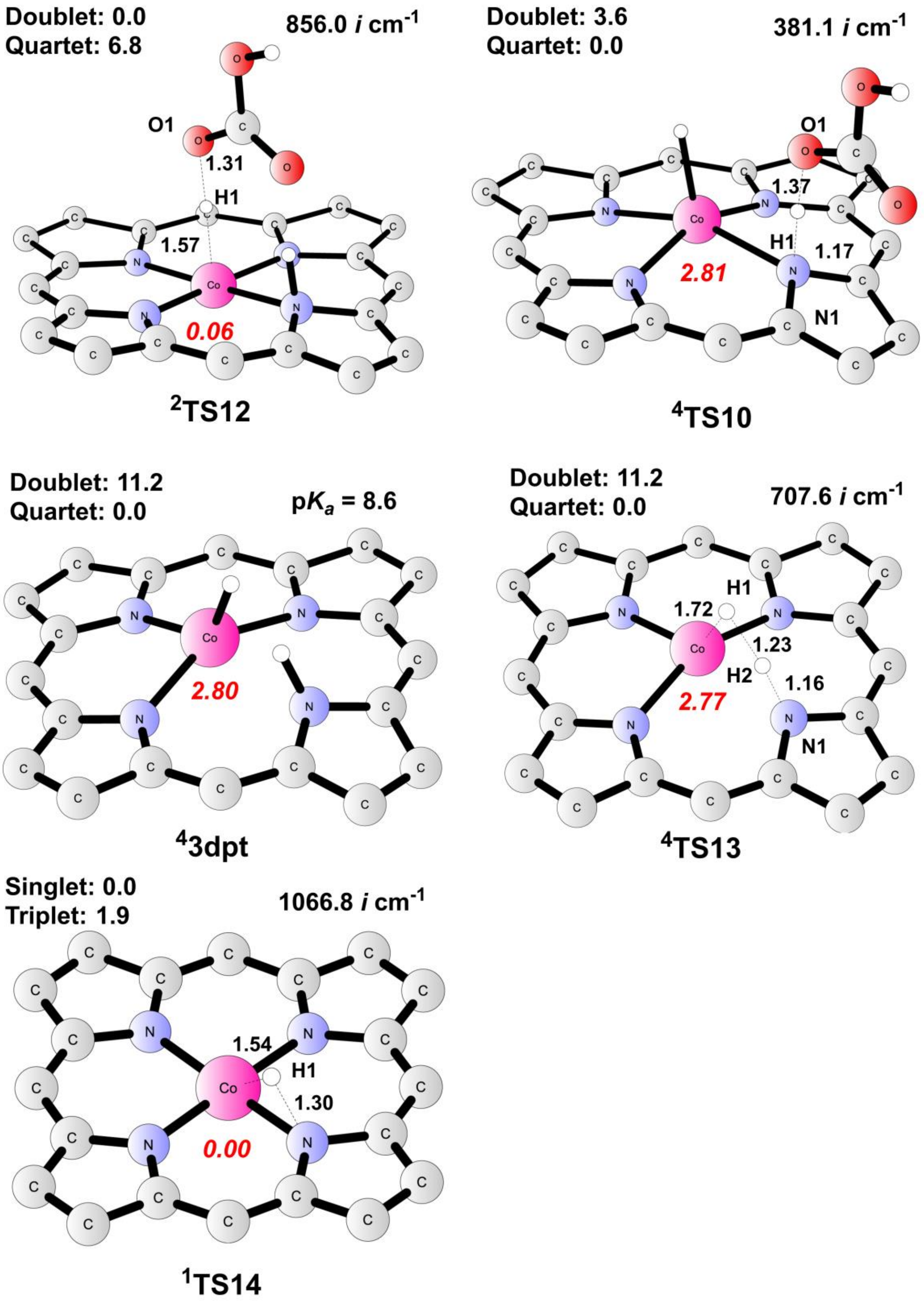


**Figure S4.** Optimized structures of transition states **TS12** (total charge of -1), **TS10** (total charge of -1), **TS13** (total charge of 0), **TS14** (total charge of 0), and intermediate **3dpt** (total charge of 0). Energies relative to the ground states are given in kcal $mol^{-1}$, and imaginary frequencies (*i* $cm^{-1}$) for all transition states are shown. Bond distances are given in Ångström. The spin populations on Co are given in red italics. Unimportant H atoms are omitted.

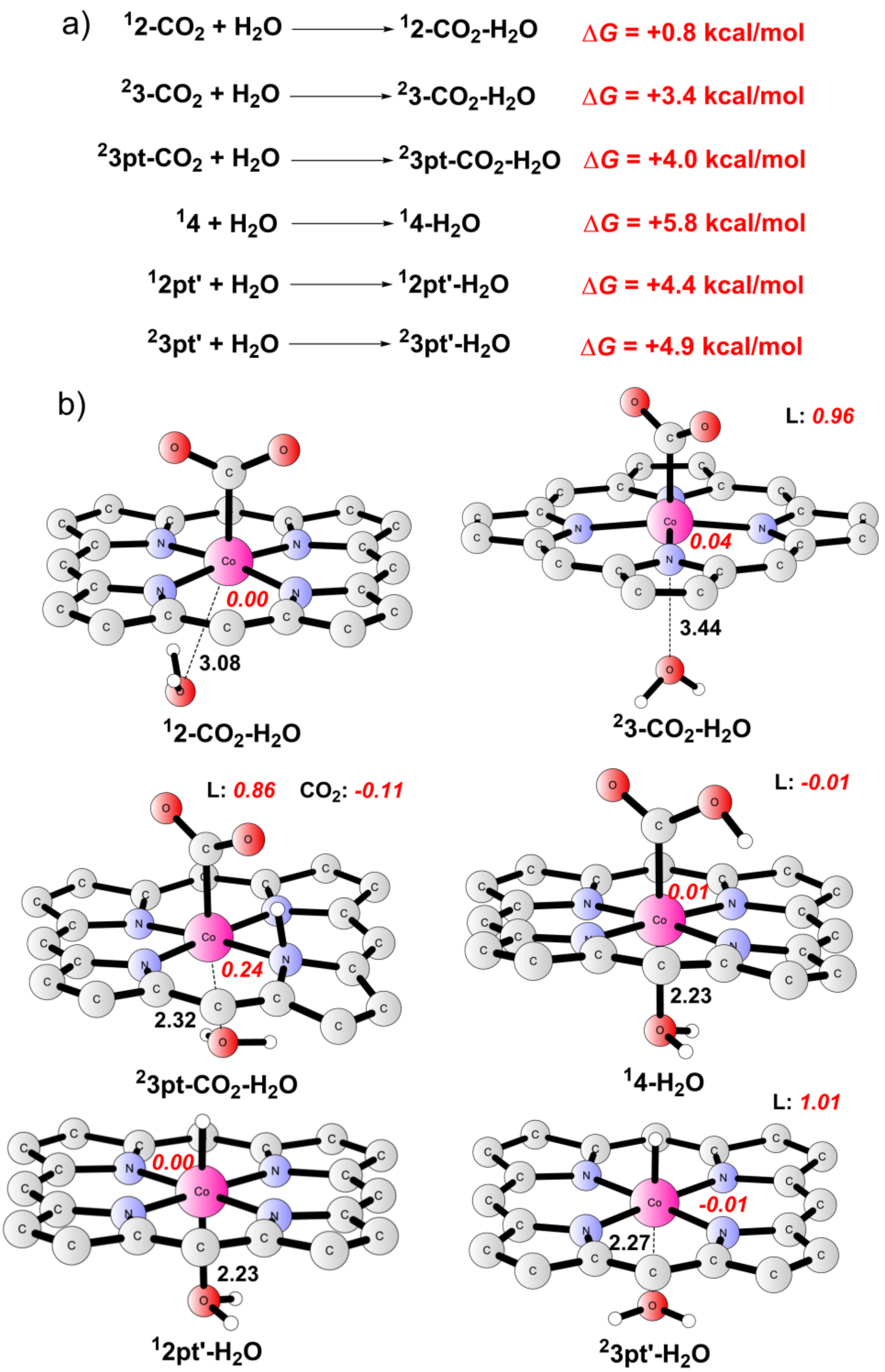


**Figure S5.** a) Gibbs free energy change (Δ$G$) for the generation of hexa-coordinated $Co^{III}$ complexes **2-$CO_2$-$H_2O$** (total charge of -1), **3-$CO_2$-$H_2O$** (total charge of -2), **3pt-$CO_2$-$H_2O$** (total charge of -1), **4-$H_2O$** (total charge of 0), **2pt'-$H_2O$** (total charge of 0), and **3pt'-$H_2O$** (total charge of -1) from the penta-coordinated intermediates are given in kcal $mol^{-1}$. b) Optimized structures of the hexa-coordinated $Co^{III}$ complexes mentioned above. Bond distances are given in Ångström. The spin populations on Co, ligands and $CO_2$ are given in red italics. Unimportant H atoms are omitted.

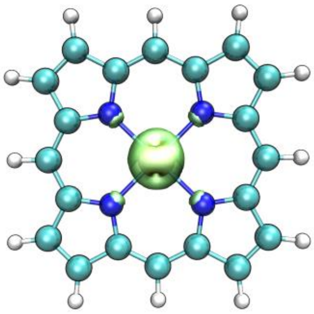

$^{4}$**1**

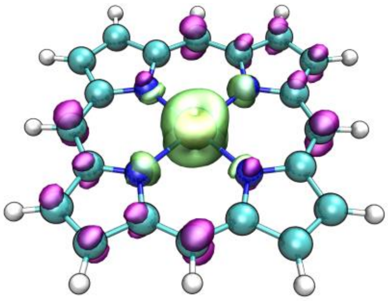

$^{3}$**2**

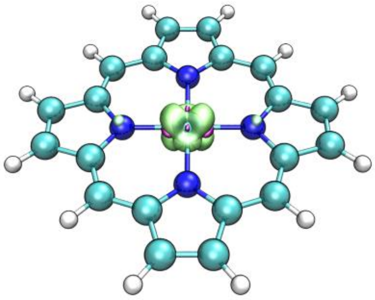

$^{2}$**3**

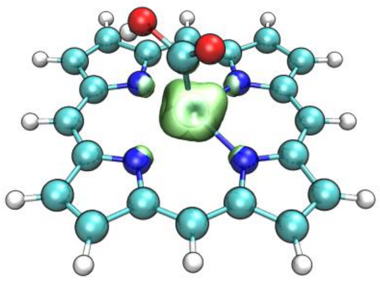

$^{4}$**5**

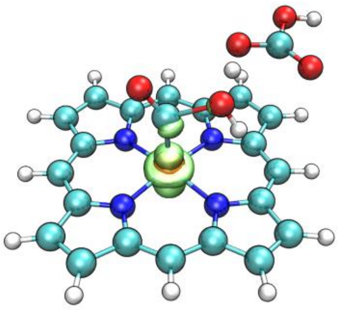

$^{2}$**TS9**

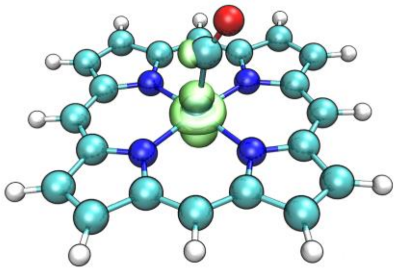

$^{2}$**6**

**Figure S6.** Spin populations of **1** (quartet), **2** (triplet), **3** (doublet), **5** (quartet), **TS9** (doublet), and **6** (doublet).

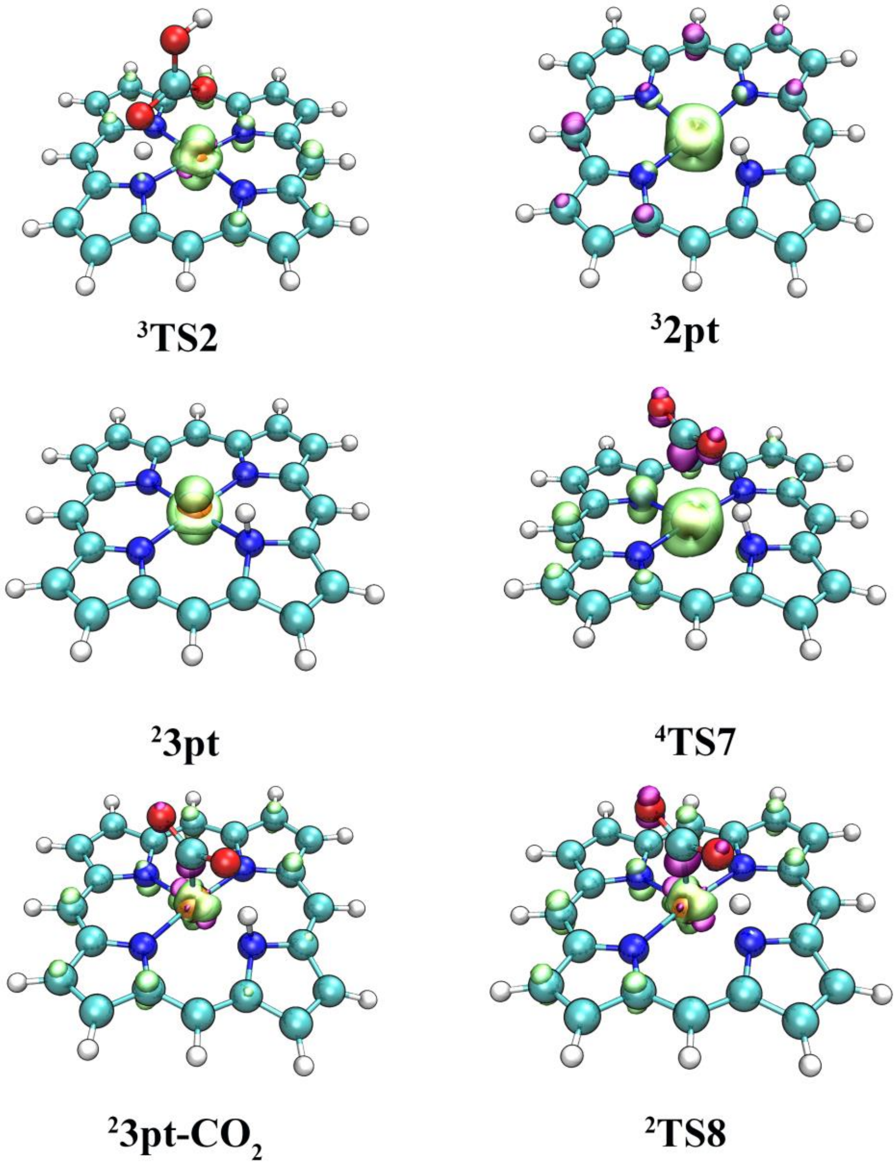


**Figure S7.** Spin populations of **TS2** (triplet), **2pt** (triplet), **3pt** (doublet), **TS7** (quartet), **3pt-$CO_2$** (doublet), and **TS8** (doublet).

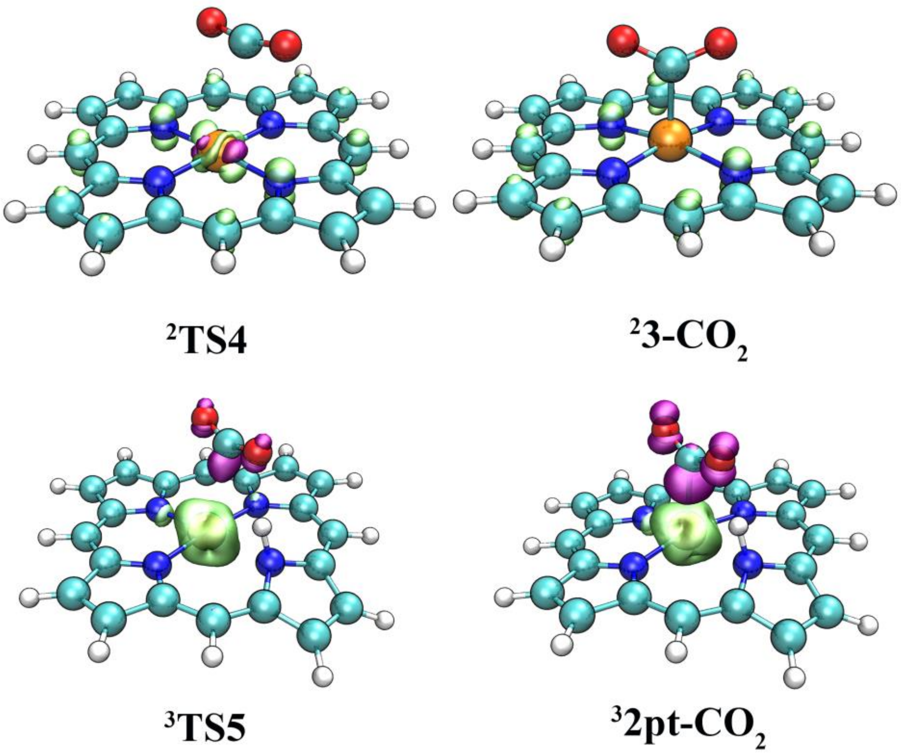


**Figure S8.** Spin populations of **TS4** (doublet), **3-$CO_2$** (doublet), **TS5** (triplet), and **2pt-$CO_2$** (triplet).

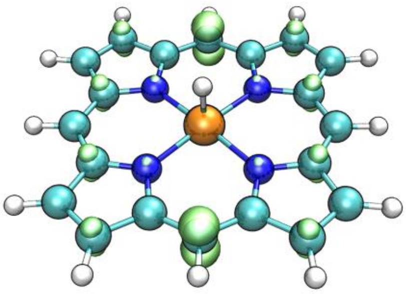

$^{2}$**3pt’**

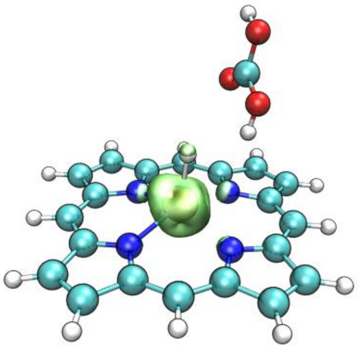

$^{4}$**TS11**

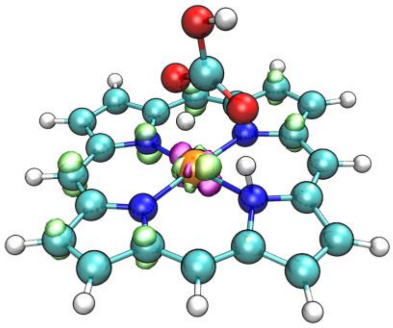

$^{2}$**TS12**

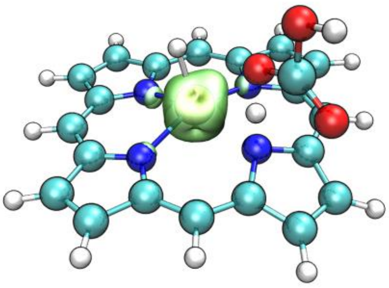

$^{4}$**TS10**

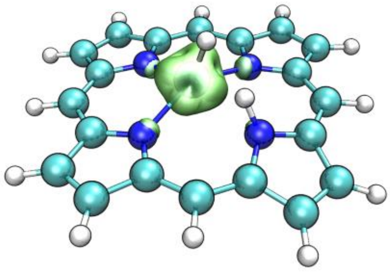

$^{4}$**3dpt**

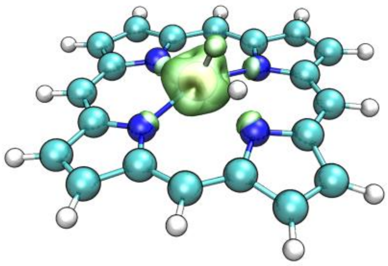

$^{4}$**TS13**

**Figure S9.** Spin populations of **3pt’** (doublet), **TS11** (quartet), **TS12** (doublet), **TS10** (quartet), **3dpt** (quartet), and **TS13** (quartet).

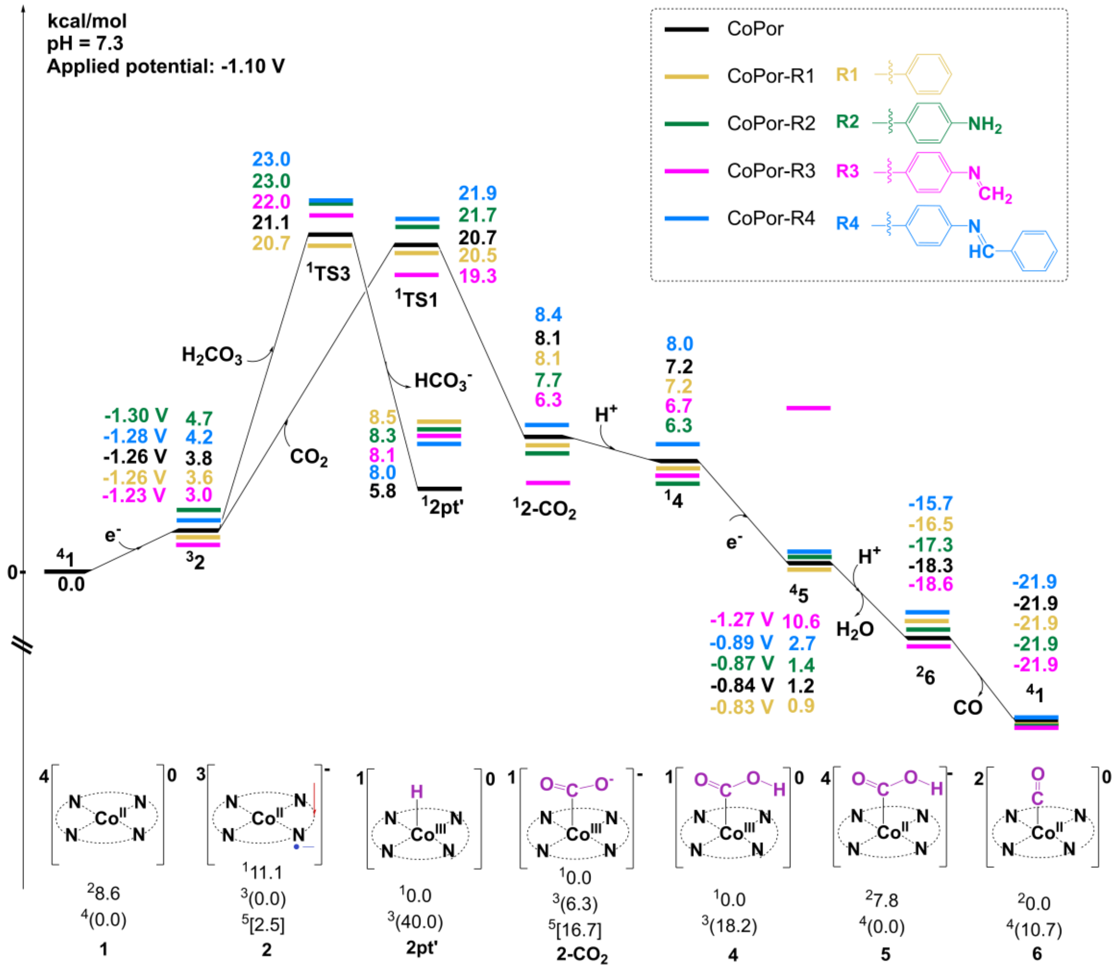


**Figure S10**. Gibbs energy diagram for critical steps of $CO_2$RR and HER. The energies based on the ground spin states are given in kcal mol$^{-1}$. An applied potential of -1.10 V and pH of 7.3 are used as the reference. Core structures for intermediates are given.

## Section 2. Energies for all stationary points

**Table S2.** Calculated energies (in Hartree) for all stationary points.

| | optimization | large basis sets | Gibbs correction |
|---|---|---|---|
| **$^{4}$1** | -1134.37325 | -1134.67307 | 0.22694 |
| **$^{2}$1-$H_2O$** | -1210.80318 | -1211.15736 | 0.25190 |
| **$^{2}$1-$H_2CO_3$** | -1399.39057 | -1399.81597 | 0.26475 |
| **$^{4}$1-$HCO_3^-$** | -1398.84652 | -1399.35838 | 0.24747 |
| **$^{2}$1-$CO_3^{2-}$** | -1398.10451 | -1398.86743 | 0.22967 |
| **$^{3}$2** | -1134.47320 | -1134.78265 | 0.22564 |
| **$^{3}$2pt** | -1134.93259 | -1135.23575 | 0.23850 |
| **$^{1}$2pt’** | -1134.93928 | -1135.24032 | 0.23996 |
| **$^{1}$2-$CO_2$** | -1323.06858 | -1323.45215 | 0.24015 |
| **$^{3}$2pt-$CO_2$** | -1323.50705 | -1323.87987 | 0.24620 |
| **$^{2}$3** | -1134.54587 | -1134.86801 | 0.22063 |
| **$^{2}$3pt** | -1135.02428 | -1135.33909 | 0.23716 |
| **$^{2}$3pt’** | -1135.03184 | -1135.34204 | 0.23182 |
| **$^{4}$3dpt** | -1135.50635 | -1135.80499 | 0.24545 |
| **$^{2}$3-$CO_2$** | -1323.15556 | -1323.55095 | 0.23459 |
| **$^{2}$3pt-$CO_2$** | -1323.62706 | -1324.01107 | 0.24734 |
| **$^{1}$4** | -1323.53808 | -1323.91175 | 0.25163 |
| **$^{4}$5** | -1323.64945 | -1324.03142 | 0.24497 |
| **$^{2}$6** | -1247.68903 | -1248.03180 | 0.23168 |
| **$^{1}$TS1** | -1323.04555 | -1323.42735 | 0.23543 |
| **$^{3}$TS2** | -1399.50132 | -1399.91448 | 0.25439 |
| **$^{1}$TS3** | -1399.49248 | -1399.90639 | 0.25678 |
| **$^{2}$TS4** | -1323.12234 | -1323.51730 | 0.23076 |
| **$^{3}$TS5** | -1323.49754 | -1323.87115 | 0.24531 |
| **$^{1}$TS6** | -1588.10885 | -1588.59670 | 0.26807 |
| **$^{4}$TS7** | -1323.52867 | -1323.98623 | 0.24064 |
| **$^{2}$TS8** | -1323.53824 | -1324.00587 | 0.24426 |
| **$^{2}$TS9** | -1588.68282 | -1589.17080 | 0.27438 |
| **$^{4}$TS10** | -1400.06385 | -1400.47581 | 0.26034 |
| **$^{4}$TS11** | -1399.97385 | -1400.48505 | 0.26116 |
| **$^{2}$TS12** | -1400.04783 | -1400.46265 | 0.26505 |
| **$^{4}$TS13** | -1135.48308 | -1135.80111 | 0.24177 |
| **$^{1}$TS14** | -1134.89042 | -1135.20520 | 0.23830 |
| **$^{4}$1pt** | -1134.72750 | -1135.10877 | 0.24116 |
| **$^{1}$2-$CO_2$-$H_2O$** | -1399.51698 | -1399.93505 | 0.26018 |
| **$^{2}$3-$CO_2$-$H_2O$** | -1399.59435 | -1400.02713 | 0.25213 |
| **$^{2}$3pt-$CO_2$-$H_2O$** | -1400.07404 | -1400.49066 | 0.26921 |
| **$^{1}$4-$H_2O$** | -1399.98832 | -1400.39365 | 0.27516 |

| | optimization | large basis sets | Gibbs correction |
|---|---|---|---|
| **$^{1}$2pt'-$H_2O$** | -1211.38812 | -1211.72046 | 0.26304 |
| **$^{2}$3pt'-$H_2O$** | -1211.47962 | -1211.82226 | 0.25570 |

# Section 3. Cartesian Coordinates of Optimized Structures

**$^{4}$1**

| | | | |
|---|---|---|---|
| C | -1.24858200 | 2.81474600 | -0.00002500 |
| C | -2.52362000 | 3.49450100 | -0.00008600 |
| C | -3.49142100 | 2.52870000 | -0.00010000 |
| C | -2.81426000 | 1.25229800 | -0.00007800 |
| N | -1.45321700 | 1.45407000 | -0.00004400 |
| C | -3.44240300 | 0.00240100 | -0.00003400 |
| C | -2.81602000 | -1.24837300 | 0.00005100 |
| C | -3.49495000 | -2.52383300 | 0.00004400 |
| C | -2.52848800 | -3.49097500 | 0.00013500 |
| C | -1.25250700 | -2.81298800 | 0.00010500 |
| N | -1.45526500 | -1.45202300 | 0.00009500 |
| C | -0.00240000 | -3.44069600 | 0.00005400 |
| C | 1.24858200 | -2.81474500 | -0.00003600 |
| C | 2.52362000 | -3.49450100 | -0.00009000 |
| C | 3.49142200 | -2.52870000 | -0.00010100 |
| C | 2.81426100 | -1.25229800 | -0.00011000 |
| N | 1.45321800 | -1.45407000 | -0.00008000 |
| C | 3.44240300 | -0.00240100 | -0.00006900 |
| C | 2.81602000 | 1.24837300 | 0.00001200 |
| C | 3.49495000 | 2.52383300 | 0.00006800 |
| C | 2.52848800 | 3.49097500 | 0.00013100 |
| C | 1.25250800 | 2.81298800 | 0.00008300 |
| N | 1.45526500 | 1.45202300 | 0.00003000 |
| C | 0.00240100 | 3.44069600 | 0.00005000 |
| H | -2.64990400 | 4.56965100 | -0.00009900 |
| H | -4.56631800 | 2.65715300 | -0.00012700 |
| H | -4.57002300 | -2.65080000 | -0.00000300 |
| H | -2.65625900 | -4.56595100 | 0.00017100 |
| H | 2.64990400 | -4.56965100 | -0.00008400 |
| H | 4.56631900 | -2.65715400 | -0.00011600 |
| H | 4.57002300 | 2.65080000 | 0.00006500 |
| H | 2.65625900 | 4.56595100 | 0.00019200 |
| H | -4.52838000 | 0.00315700 | -0.00007500 |
| H | -0.00315400 | -4.52668800 | 0.00008000 |
| H | 4.52838000 | -0.00315700 | -0.00008700 |
| H | 0.00315500 | 4.52668800 | 0.00008200 |
| Co | -0.00000100 | 0.00000000 | -0.00000100 |

**$^{2}$1-$H_2O$**

| | | | |
|---|---|---|---|
| C | 2.77611000 | -1.22843500 | -0.14360600 |
| C | 3.45158700 | -2.50200400 | -0.16634100 |
| C | 2.48427700 | -3.45988600 | -0.15749400 |
| C | 1.21743700 | -2.77158200 | -0.13162500 |
| N | 1.40958500 | -1.40630300 | -0.11929300 |
| C | -0.01576100 | -3.40747800 | -0.12673900 |
| C | -1.24303100 | -2.76022200 | -0.13180500 |
| C | -2.51618000 | -3.43675300 | -0.15797700 |
| C | -3.47458300 | -2.46995300 | -0.16683000 |
| C | -2.78735500 | -1.20270100 | -0.14377200 |
| N | -1.42253600 | -1.39320900 | -0.11928100 |
| C | -3.42205400 | 0.03092500 | -0.14404900 |
| C | -2.77160100 | 1.25585400 | -0.13204900 |
| C | -3.44969000 | 2.53048400 | -0.12879800 |
| C | -2.48315100 | 3.48661400 | -0.10790600 |
| C | -1.21586000 | 2.79524900 | -0.10033000 |
| N | -1.40731800 | 1.43364800 | -0.11585300 |
| C | 0.01589200 | 3.43191100 | -0.08656100 |
| C | 1.24170000 | 2.78387700 | -0.10046100 |
| C | 2.51533600 | 3.46349500 | -0.10828200 |
| C | 3.47298000 | 2.49846000 | -0.12920200 |
| C | 2.78312100 | 1.23016000 | -0.13222100 |
| N | 1.42055600 | 1.42056500 | -0.11585800 |
| C | 3.42220400 | -0.00074000 | -0.14407400 |
| H | 4.52626700 | -2.62667000 | -0.18967500 |
| H | 2.59817900 | -4.53592400 | -0.17323500 |
| H | -2.64004300 | -4.51168700 | -0.17389700 |
| H | -4.55036600 | -2.58467600 | -0.19035200 |
| H | -4.52463300 | 2.65405600 | -0.14196400 |
| H | -2.59526500 | 4.56284600 | -0.10054200 |
| H | 2.63740000 | 4.53864500 | -0.10102700 |
| H | 4.54901900 | 2.61208000 | -0.14249800 |
| H | -0.02076600 | -4.49276400 | -0.13698000 |
| H | -4.50721500 | 0.03778000 | -0.15886300 |
| H | 0.02091200 | 4.51707200 | -0.07374400 |
| H | 4.50738200 | -0.00393800 | -0.15890300 |
| Co | 0.00012200 | 0.02090400 | -0.06159000 |
| O | -0.00071700 | -0.15392800 | 2.24323300 |
| H | -0.77059000 | -0.73848500 | 2.29536400 |
| H | 0.76188900 | -0.74785900 | 2.29609400 |

**$^{2}$1-$H_2CO_3$**

| | | | |
|---|---|---|---|
| C | 1.22827500 | -2.82621700 | -0.35861200 |
| C | 0.81686800 | -4.20988000 | -0.36627500 |
| C | -0.53941800 | -4.21376900 | -0.46174000 |
| C | -0.95567200 | -2.83201800 | -0.51209400 |
| N | 0.13488700 | -1.99577800 | -0.44071600 |
| C | -2.27686000 | -2.42890600 | -0.64834500 |
| C | -2.70071700 | -1.11110100 | -0.71627100 |
| C | -4.05763100 | -0.69384300 | -0.93573600 |
| C | -4.06100200 | 0.67053700 | -0.93789400 |
| C | -2.70618500 | 1.09517000 | -0.71958000 |
| N | -1.87556200 | -0.00568000 | -0.56332900 |
| C | -2.28907200 | 2.41527000 | -0.65455400 |
| C | -0.97015500 | 2.82539100 | -0.51728500 |
| C | -0.56114100 | 4.20930200 | -0.46767500 |
| C | 0.79494800 | 4.21252200 | -0.36930400 |
| C | 1.21352600 | 2.83105200 | -0.35970600 |
| N | 0.12460500 | 1.99485200 | -0.44307800 |
| C | 2.53706300 | 2.42187200 | -0.29838400 |
| C | 2.95890400 | 1.10060800 | -0.30963300 |
| C | 4.34217700 | 0.69046100 | -0.24383100 |
| C | 4.34576900 | -0.66932200 | -0.24450200 |
| C | 2.96466900 | -1.08670400 | -0.31047700 |
| N | 2.13274800 | 0.00478700 | -0.36176600 |
| C | 2.54974700 | -2.41015500 | -0.29922500 |
| H | 1.49418100 | -5.05207700 | -0.31280700 |
| H | -1.21324800 | -5.05940000 | -0.50336300 |
| H | -4.89166500 | -1.36908500 | -1.07403000 |
| H | -4.89837100 | 1.34121000 | -1.07825000 |
| H | -1.23924400 | 5.05139800 | -0.51151000 |
| H | 1.46778400 | 5.05827000 | -0.31534000 |
| H | 5.18311300 | 1.37012100 | -0.20123600 |
| H | 5.19028100 | -1.34457600 | -0.20258400 |
| H | -3.03219100 | -3.20352700 | -0.73184200 |
| H | -3.04828800 | 3.18582400 | -0.74038900 |
| H | 3.29906600 | 3.19274500 | -0.24384200 |
| H | 3.31581100 | -3.17704900 | -0.24549200 |
| Co | 0.14166500 | -0.00035000 | -0.37696300 |
| O | 0.25551900 | 0.00184700 | 1.93158100 |
| C | -0.72180800 | 0.00260700 | 2.67103700 |
| O | -1.98446700 | 0.00012000 | 2.29261900 |
| O | -0.62996400 | 0.00621600 | 4.00659700 |
| H | -1.98872200 | -0.00229300 | 1.30409500 |
| H | 0.31739400 | 0.00789400 | 4.21094900 |

**$^{4}$1-HCO$_3^-$**

| | | | |
|---|---|---|---|
| C | -2.61110600 | 1.87195300 | -0.24608900 |
| C | -2.99762000 | 3.26993600 | -0.20576700 |
| C | -1.84405900 | 3.99740600 | -0.24023900 |
| C | -0.75087400 | 3.04476100 | -0.30234100 |
| N | -1.24893300 | 1.77236800 | -0.29931700 |
| C | 0.60373000 | 3.38327900 | -0.40005800 |
| C | 1.68437700 | 2.50606200 | -0.54636300 |
| C | 3.05375300 | 2.90215100 | -0.80124100 |
| C | 3.77749500 | 1.75359000 | -0.95559100 |
| C | 2.85457900 | 0.65101100 | -0.79132000 |
| N | 1.59864600 | 1.13770300 | -0.52650500 |
| C | 3.18226400 | -0.70513200 | -0.88302800 |
| C | 2.30089600 | -1.78718600 | -0.78953300 |
| C | 2.67677600 | -3.18199600 | -0.91877000 |
| C | 1.52935400 | -3.91016700 | -0.80137500 |
| C | 0.44919800 | -2.96164500 | -0.60570600 |
| N | 0.95109400 | -1.69002200 | -0.59278400 |
| C | -0.90505700 | -3.29929500 | -0.50011200 |
| C | -1.98978900 | -2.42027900 | -0.40673500 |
| C | -3.38637900 | -2.81426200 | -0.36724800 |
| C | -4.11736200 | -1.66416900 | -0.30395800 |
| C | -3.16793100 | -0.56631700 | -0.30212500 |
| N | -1.89614600 | -1.05880100 | -0.36701900 |
| C | -3.49990600 | 0.79132700 | -0.24320400 |
| H | -4.01758900 | 3.63243800 | -0.16354800 |
| H | -1.73052500 | 5.07473000 | -0.22982900 |
| H | 3.40270900 | 3.92565700 | -0.86543100 |
| H | 4.83486000 | 1.65241300 | -1.16737700 |
| H | 3.68577100 | -3.54140800 | -1.07969100 |
| H | 1.41065000 | -4.98580300 | -0.84900400 |
| H | -3.74546400 | -3.83610900 | -0.38656600 |
| H | -5.19441800 | -1.55610400 | -0.26237700 |
| H | 0.83798700 | 4.44485000 | -0.41225800 |
| H | 4.22813400 | -0.94236900 | -1.05908500 |
| H | -1.14152100 | -4.36013300 | -0.52262900 |
| H | -4.55868300 | 1.03248900 | -0.19130100 |
| Co | -0.13142100 | 0.01272600 | -0.03048700 |
| O | -0.12984000 | -0.07616000 | 1.95337300 |
| C | 0.83784400 | -0.30281500 | 2.79211200 |
| O | 2.07113900 | 0.21820900 | 2.42020200 |
| O | 0.76934100 | -0.89497700 | 3.85833700 |
| H | 1.93761300 | 0.66117100 | 1.56845700 |

**$^{2}$1-$CO_3^{2-}$**

| | | | |
|---|---|---|---|
| C | -1.12132400 | -2.91018800 | -0.40627300 |
| C | -2.29966100 | -3.71448300 | -0.32943500 |
| C | -3.36251400 | -2.84231900 | -0.18517300 |
| C | -2.81074000 | -1.52568200 | -0.17817600 |
| N | -1.44073100 | -1.57429300 | -0.31672600 |
| C | -3.56287300 | -0.34190500 | -0.06670600 |
| C | -3.04960000 | 0.93760700 | -0.11428800 |
| C | -3.86130500 | 2.13849600 | -0.06455100 |
| C | -3.01261900 | 3.19474400 | -0.17996800 |
| C | -1.67279900 | 2.64900200 | -0.29694500 |
| N | -1.71872200 | 1.27341800 | -0.24848900 |
| C | -0.53684500 | 3.41875400 | -0.45058400 |
| C | 0.77084700 | 2.91563400 | -0.58662200 |
| C | 1.94178400 | 3.71959000 | -0.74789300 |
| C | 3.01037500 | 2.84725000 | -0.82417400 |
| C | 2.46936000 | 1.53114700 | -0.71293800 |
| N | 1.10025200 | 1.58052300 | -0.57604400 |
| C | 3.22416100 | 0.34816000 | -0.73803900 |
| C | 2.71117500 | -0.93094000 | -0.70159700 |
| C | 3.52341500 | -2.13028800 | -0.76354500 |
| C | 2.67101200 | -3.18758400 | -0.72773700 |
| C | 1.32818200 | -2.64211200 | -0.63323800 |
| N | 1.37716200 | -1.26661600 | -0.63709400 |
| C | 0.18592200 | -3.41240300 | -0.54707800 |
| H | -2.31424400 | -4.79808200 | -0.37656100 |
| H | -4.41843100 | -3.07188200 | -0.08920200 |
| H | -4.94117500 | 2.14501000 | 0.03986300 |
| H | -3.24615900 | 4.25414400 | -0.18881400 |
| H | 1.94845800 | 4.80372900 | -0.78320600 |
| H | 4.06446100 | 3.07576800 | -0.93753200 |
| H | 4.60653900 | -2.13398500 | -0.81900200 |
| H | 2.90257300 | -4.24717700 | -0.74513500 |
| H | -4.64044500 | -0.44715300 | 0.04112400 |
| H | -0.65914300 | 4.49953900 | -0.46887700 |
| H | 4.30504600 | 0.45189400 | -0.79851400 |
| H | 0.30396300 | -4.49355800 | -0.57039100 |
| Co | -0.14146400 | 0.00010900 | -0.27873100 |
| O | 0.05212000 | -0.03881800 | 1.87140000 |
| C | 1.03911100 | -0.01660100 | 2.68571600 |
| O | 0.83247200 | 0.04818100 | 3.94876500 |
| O | 2.26190400 | -0.06032500 | 2.31557200 |

**$^{3}$2**

| | | | |
|---|---|---|---|
| C | 2.88506300 | 1.10407500 | 0.00000300 |
| C | 4.26933900 | 0.66493800 | 0.00000600 |
| C | 4.26420900 | -0.69785000 | 0.00000600 |
| C | 2.87657800 | -1.12645200 | 0.00000400 |
| N | 2.05955500 | -0.00813700 | 0.00000200 |
| C | 2.44537400 | -2.44367000 | 0.00000500 |
| C | 1.09982200 | -2.90044700 | 0.00000300 |
| C | 0.67879300 | -4.26078500 | 0.00000400 |
| C | -0.71167900 | -4.25560500 | 0.00000200 |
| C | -1.12241500 | -2.89207900 | 0.00000000 |
| N | -0.00823300 | -2.07292900 | 0.00000100 |
| C | -2.46427500 | -2.42466200 | -0.00000200 |
| C | -2.88506300 | -1.10407500 | -0.00000300 |
| C | -4.26933900 | -0.66493800 | -0.00000600 |
| C | -4.26420900 | 0.69785000 | -0.00000600 |
| C | -2.87657800 | 1.12645200 | -0.00000400 |
| N | -2.05955500 | 0.00813700 | -0.00000200 |
| C | -2.44537400 | 2.44367000 | -0.00000500 |
| C | -1.09982200 | 2.90044700 | -0.00000300 |
| C | -0.67879300 | 4.26078500 | -0.00000400 |
| C | 0.71167900 | 4.25560500 | -0.00000200 |
| C | 1.12241500 | 2.89207900 | 0.00000000 |
| N | 0.00823300 | 2.07292900 | -0.00000100 |
| C | 2.46427500 | 2.42466200 | 0.00000200 |
| H | 5.12553600 | 1.32879400 | 0.00000700 |
| H | 5.11537700 | -1.36811900 | 0.00000900 |
| H | 1.34058500 | -5.11854000 | 0.00000600 |
| H | -1.37982600 | -5.10840400 | 0.00000200 |
| H | -5.12553600 | -1.32879400 | -0.00000700 |
| H | -5.11537700 | 1.36811900 | -0.00000900 |
| H | -1.34058500 | 5.11854000 | -0.00000600 |
| H | 1.37982600 | 5.10840400 | -0.00000200 |
| H | 3.21496700 | -3.21085800 | 0.00000700 |
| H | -3.23984100 | -3.18581800 | -0.00000200 |
| H | -3.21496700 | 3.21085800 | -0.00000700 |
| H | 3.23984100 | 3.18581800 | 0.00000200 |
| Co | 0.00000000 | 0.00000000 | 0.00000000 |

**[3]2pt**

| | | | |
|---|---|---|---|
| C | -2.94824300 | -1.15745900 | 0.06822100 |
| C | -4.27490600 | -0.68458100 | -0.20306500 |
| C | -4.27491000 | 0.68456000 | -0.20306600 |
| C | -2.94825100 | 1.15745100 | 0.06822100 |
| N | -2.12119500 | 0.00000000 | 0.29518100 |
| C | -2.46771000 | 2.43769200 | 0.04991400 |
| C | -1.10726200 | 2.86180400 | 0.09517300 |
| C | -0.69044400 | 4.21979500 | 0.14223400 |
| C | 0.69472300 | 4.21964100 | 0.11337500 |
| C | 1.10998800 | 2.85755600 | 0.04816300 |
| N | -0.00048600 | 2.03151200 | 0.03755600 |
| C | 2.45921000 | 2.42316600 | 0.00500500 |
| C | 2.90869300 | 1.11258300 | -0.02920600 |
| C | 4.29442200 | 0.68167700 | -0.02980500 |
| C | 4.29442300 | -0.68167300 | -0.02980300 |
| C | 2.90869500 | -1.11257700 | -0.02919600 |
| N | 2.09149200 | 0.00000200 | -0.04010600 |
| C | 2.45921200 | -2.42315800 | 0.00502100 |
| C | 1.10999000 | -2.85755200 | 0.04817000 |
| C | 0.69473100 | -4.21964000 | 0.11336700 |
| C | -0.69043400 | -4.21979800 | 0.14222500 |
| C | -1.10724900 | -2.86180500 | 0.09516800 |
| N | -0.00048000 | -2.03151000 | 0.03756500 |
| C | -2.46769600 | -2.43769400 | 0.04991400 |
| H | -5.11004300 | -1.33518600 | -0.42912600 |
| H | -5.11005300 | 1.33515800 | -0.42912800 |
| H | -1.35673900 | 5.07199700 | 0.18938600 |
| H | 1.36273800 | 5.07142200 | 0.13711300 |
| H | 5.14687100 | 1.34945200 | -0.02210500 |
| H | 5.14687200 | -1.34944900 | -0.02210100 |
| H | 1.36274800 | -5.07142000 | 0.13710200 |
| H | -1.35672700 | -5.07200100 | 0.18937500 |
| H | -3.20715700 | 3.22744400 | -0.05052900 |
| H | 3.21450700 | 3.20353200 | 0.01527800 |
| H | 3.21451000 | -3.20352300 | 0.01529700 |
| H | -3.20714000 | -3.22744900 | -0.05052700 |
| Co | 0.07934700 | 0.00000200 | -0.23590900 |
| H | -1.76995300 | 0.00000000 | 1.25795400 |

**[1]2pt'**

| | | | |
|---|---|---|---|
| C | 2.98633600 | 0.52480400 | -0.01674900 |
| C | 4.25792800 | -0.15064800 | -0.01047600 |
| C | 3.99329900 | -1.48598500 | -0.01036000 |
| C | 2.56006200 | -1.62546000 | -0.01663000 |
| N | 1.94824800 | -0.38698200 | -0.03083300 |
| C | 1.90206600 | -2.84355000 | -0.01062200 |
| C | 0.52480500 | -2.98633700 | -0.01671400 |
| C | -0.15064800 | -4.25792800 | -0.01047900 |
| C | -1.48598500 | -3.99329900 | -0.01041800 |
| C | -1.62545900 | -2.56006200 | -0.01669300 |
| N | -0.38697900 | -1.94824800 | -0.03084000 |
| C | -2.84355000 | -1.90206600 | -0.01070900 |
| C | -2.98633700 | -0.52480500 | -0.01675600 |
| C | -4.25792900 | 0.15064800 | -0.01049000 |
| C | -3.99330000 | 1.48598500 | -0.01037200 |
| C | -2.56006200 | 1.62545900 | -0.01664100 |
| N | -1.94824900 | 0.38698000 | -0.03083900 |
| C | -1.90206500 | 2.84354900 | -0.01063400 |
| C | -0.52480400 | 2.98633600 | -0.01672300 |
| C | 0.15064800 | 4.25792800 | -0.01048200 |
| C | 1.48598500 | 3.99330000 | -0.01042800 |
| C | 1.62546000 | 2.56006200 | -0.01669400 |
| N | 0.38698100 | 1.94824900 | -0.03084200 |
| C | 2.84355000 | 1.90206600 | -0.01070500 |
| H | 5.21532500 | 0.35370400 | -0.00810300 |
| H | 4.68628500 | -2.31705100 | -0.00795100 |
| H | 0.35370300 | -5.21532600 | -0.00808500 |
| H | -2.31705100 | -4.68628400 | -0.00804500 |
| H | -5.21532700 | -0.35370300 | -0.00811900 |
| H | -4.68628500 | 2.31705100 | -0.00796500 |
| H | -0.35370300 | 5.21532600 | -0.00808900 |
| H | 2.31705100 | 4.68628600 | -0.00805800 |
| H | 2.50542500 | -3.74499400 | 0.00060300 |
| H | -3.74499300 | -2.50542600 | 0.00048000 |
| H | -2.50542400 | 3.74499300 | 0.00058900 |
| H | 3.74499400 | 2.50542400 | 0.00048400 |
| Co | -0.00000100 | 0.00000100 | 0.03820400 |
| H | -0.00000100 | 0.00000000 | 1.45286800 |

**[1]2-$CO_2$**

| | | | |
|---|---|---|---|
| C | -1.00059900 | 2.87143700 | -0.26677500 |
| C | -2.21522700 | 3.64919200 | -0.26010900 |
| C | -3.24755200 | 2.76140600 | -0.26321000 |
| C | -2.66124000 | 1.44337900 | -0.27168600 |
| N | -1.28467800 | 1.52182400 | -0.27974000 |
| C | -3.39976900 | 0.27006000 | -0.27806800 |
| C | -2.85692800 | -1.00541600 | -0.29786200 |
| C | -3.64402800 | -2.21428700 | -0.30788700 |
| C | -2.76476300 | -3.25388700 | -0.30377200 |
| C | -1.44266500 | -2.67713600 | -0.29197900 |
| N | -1.51017100 | -1.29968500 | -0.30098900 |
| C | -0.27241700 | -3.42014000 | -0.26923500 |
| C | 1.00058700 | -2.87143400 | -0.26675900 |
| C | 2.21521600 | -3.64918900 | -0.26012300 |
| C | 3.24754100 | -2.76140500 | -0.26325500 |
| C | 2.66122800 | -1.44337800 | -0.27167000 |
| N | 1.28466700 | -1.52181900 | -0.27967500 |
| C | 3.39976300 | -0.27006200 | -0.27809200 |
| C | 2.85692300 | 1.00541400 | -0.29790200 |
| C | 3.64402100 | 2.21428600 | -0.30783800 |
| C | 2.76475300 | 3.25388400 | -0.30376300 |
| C | 1.44265600 | 2.67713400 | -0.29199500 |
| N | 1.51016400 | 1.29968100 | -0.30106400 |
| C | 0.27240900 | 3.42013800 | -0.26923800 |
| H | -2.24832600 | 4.73104300 | -0.25858200 |
| H | -4.31219100 | 2.95660800 | -0.26597600 |
| H | -4.72608900 | -2.23910800 | -0.31925100 |
| H | -2.96787700 | -4.31699500 | -0.31005800 |
| H | 2.24831300 | -4.73104100 | -0.25862200 |
| H | 4.31218000 | -2.95660600 | -0.26606100 |
| H | 4.72608200 | 2.23911000 | -0.31915300 |
| H | 2.96786500 | 4.31699400 | -0.31002300 |
| H | -4.48153700 | 0.35668100 | -0.27166300 |
| H | -0.35854600 | -4.50175000 | -0.25837800 |
| H | 4.48153000 | -0.35668800 | -0.27168800 |
| H | 0.35854100 | 4.50174800 | -0.25833000 |
| Co | 0.00000200 | -0.00000600 | -0.12865500 |
| C | 0.00003500 | 0.00000800 | 1.82945600 |
| O | 1.13025200 | -0.04002700 | 2.35765400 |
| O | -1.13019300 | 0.04004300 | 2.35763400 |

**$^{3}$2pt-$CO_2$**

| | | | |
|---|---|---|---|
| C | -2.92362400 | -1.24859900 | -0.32811100 |
| C | -4.21043200 | -0.85665700 | -0.78898000 |
| C | -4.27646900 | 0.52482500 | -0.77066300 |
| C | -3.03273000 | 1.02502800 | -0.29699100 |
| N | -2.21389300 | -0.07957200 | -0.00637300 |
| C | -2.58974400 | 2.34060900 | -0.20857500 |
| C | -1.26218700 | 2.78971900 | -0.12167800 |
| C | -0.89807300 | 4.18806900 | -0.13542000 |
| C | 0.46414300 | 4.24926400 | -0.18191200 |
| C | 0.95128200 | 2.88887700 | -0.20942800 |
| N | -0.11772400 | 2.01766900 | -0.14829700 |
| C | 2.30432800 | 2.54536400 | -0.33977100 |
| C | 2.84663100 | 1.25969400 | -0.44281400 |
| C | 4.25028400 | 0.90829200 | -0.55104100 |
| C | 4.31766600 | -0.45640400 | -0.57103500 |
| C | 2.95581500 | -0.94540100 | -0.47501400 |
| N | 2.09933100 | 0.11711900 | -0.42184800 |
| C | 2.53623500 | -2.27760500 | -0.41084100 |
| C | 1.21911100 | -2.74322100 | -0.29765800 |
| C | 0.85757500 | -4.14242200 | -0.27948600 |
| C | -0.50423000 | -4.20416400 | -0.23023100 |
| C | -0.99277000 | -2.84344400 | -0.20270400 |
| N | 0.07618200 | -1.97188800 | -0.23249600 |
| C | -2.35726300 | -2.51797800 | -0.27282400 |
| H | -4.96575900 | -1.54167500 | -1.15064200 |
| H | -5.09414600 | 1.14396100 | -1.11506000 |
| H | -1.60734200 | 5.00539900 | -0.12724900 |
| H | 1.09522400 | 5.12756300 | -0.22202900 |
| H | 5.06517900 | 1.61897200 | -0.59926200 |
| H | 5.19804900 | -1.08207500 | -0.63944800 |
| H | 1.56554600 | -4.95969300 | -0.32595900 |
| H | -1.13728400 | -5.08190400 | -0.22552300 |
| H | -3.34541000 | 3.11213000 | -0.32024800 |
| H | 3.00411700 | 3.37489900 | -0.37262400 |
| H | 3.30924300 | -3.03869900 | -0.45636100 |
| H | -3.03846900 | -3.35453100 | -0.39483500 |
| Co | 0.14782200 | 0.03639000 | -0.00628400 |
| H | -1.87442000 | -0.08494200 | 0.98049300 |
| C | 0.06528600 | -0.18634100 | 2.10291800 |
| O | -1.10919600 | -0.20342400 | 2.51113800 |
| O | 1.18444700 | -0.24535600 | 2.59824700 |

**$^{2}$3**

| | | | |
|---|---|---|---|
| C | 1.03722000 | 2.86379300 | 0.00000000 |
| C | 0.55802100 | 4.23677300 | -0.00000400 |
| C | -0.79461300 | 4.19935500 | -0.00001200 |
| C | -1.19645000 | 2.80174200 | -0.00000800 |
| N | -0.05585500 | 1.97770500 | 0.00000000 |
| C | -2.49480400 | 2.38560700 | -0.00001500 |
| C | -2.91382900 | 1.02390500 | -0.00001400 |
| C | -4.25079000 | 0.58270300 | -0.00001400 |
| C | -4.21142800 | -0.81849600 | -0.00000900 |
| C | -2.85182600 | -1.18433200 | -0.00000800 |
| N | -2.03820700 | -0.05650800 | -0.00001300 |
| C | -2.35703400 | -2.52041600 | -0.00000200 |
| C | -1.03722000 | -2.86379300 | 0.00000400 |
| C | -0.55802100 | -4.23677300 | 0.00001500 |
| C | 0.79461300 | -4.19935500 | 0.00002100 |
| C | 1.19645000 | -2.80174200 | 0.00001300 |
| N | 0.05585500 | -1.97770500 | 0.00000100 |
| C | 2.49480400 | -2.38560700 | 0.00001600 |
| C | 2.91382900 | -1.02390500 | 0.00001100 |
| C | 4.25079000 | -0.58270300 | 0.00000900 |
| C | 4.21142800 | 0.81849600 | 0.00000300 |
| C | 2.85182600 | 1.18433200 | 0.00000600 |
| N | 2.03820700 | 0.05650800 | 0.00000800 |
| C | 2.35703400 | 2.52041600 | 0.00000400 |
| H | 1.20722200 | 5.10481400 | -0.00000300 |
| H | -1.49091400 | 5.03009600 | -0.00001900 |
| H | -5.11936700 | 1.23128200 | -0.00001400 |
| H | -5.04242400 | -1.51461400 | -0.00000700 |
| H | -1.20722200 | -5.10481400 | 0.00001900 |
| H | 1.49091400 | -5.03009600 | 0.00003200 |
| H | 5.11936700 | -1.23128200 | 0.00000900 |
| H | 5.04242400 | 1.51461400 | 0.00000000 |
| H | -3.27274000 | 3.14456900 | -0.00002000 |
| H | -3.09140700 | -3.32164100 | 0.00000200 |
| H | 3.27274000 | -3.14456900 | 0.00002400 |
| H | 3.09140700 | 3.32164100 | 0.00000300 |
| Co | 0.00000000 | 0.00000000 | -0.00000400 |

**[2]3pt**

| | | | |
|---|---|---|---|
| C | -2.90534400 | -1.15858100 | 0.00868600 |
| C | -4.24460100 | -0.67822800 | -0.23232200 |
| C | -4.24460200 | 0.67821300 | -0.23233200 |
| C | -2.90534600 | 1.15857200 | 0.00867500 |
| N | -2.04340400 | -0.00000300 | 0.28947900 |
| C | -2.45081400 | 2.42928300 | -0.02275900 |
| C | -1.09418300 | 2.85951000 | 0.06275200 |
| C | -0.69340200 | 4.20604000 | 0.08450900 |
| C | 0.70405600 | 4.21421300 | 0.07393800 |
| C | 1.11627000 | 2.86507600 | 0.03674300 |
| N | 0.01940500 | 2.01878100 | 0.04070100 |
| C | 2.47050100 | 2.42206500 | -0.01848000 |
| C | 2.86212200 | 1.11554500 | -0.08864700 |
| C | 4.24985200 | 0.67688800 | -0.08519200 |
| C | 4.24985500 | -0.67687500 | -0.08520000 |
| C | 2.86212600 | -1.11553700 | -0.08865800 |
| N | 2.02225000 | 0.00000200 | -0.13787100 |
| C | 2.47050900 | -2.42205900 | -0.01850000 |
| C | 1.11627900 | -2.86507300 | 0.03673300 |
| C | 0.70406800 | -4.21421000 | 0.07392300 |
| C | -0.69339000 | -4.20604000 | 0.08452600 |
| C | -1.09417400 | -2.85951200 | 0.06276100 |
| N | 0.01941200 | -2.01877900 | 0.04070100 |
| C | -2.45080700 | -2.42929100 | -0.02274200 |
| H | -5.08101000 | -1.33604500 | -0.43385000 |
| H | -5.08101200 | 1.33602600 | -0.43386800 |
| H | -1.36903600 | 5.05307400 | 0.10361300 |
| H | 1.37059600 | 5.06812200 | 0.07493800 |
| H | 5.09812600 | 1.35086000 | -0.06823600 |
| H | 5.09813100 | -1.35084400 | -0.06825100 |
| H | 1.37061100 | -5.06811800 | 0.07491000 |
| H | -1.36902100 | -5.05307600 | 0.10364400 |
| H | -3.19444400 | 3.21045000 | -0.16188500 |
| H | 3.24271200 | 3.18541000 | 0.00481300 |
| H | 3.24272200 | -3.18540300 | 0.00478300 |
| H | -3.19443500 | -3.21046200 | -0.16185700 |
| Co | 0.05428900 | 0.00000000 | 0.00711300 |
| H | -1.89722100 | 0.00000300 | 1.30763900 |

**[2]3pt’**

| | | | |
|---|---|---|---|
| C | -2.79489600 | 1.22845700 | -0.01162400 |
| C | -3.46785900 | 2.48829500 | -0.00684900 |
| C | -2.50102600 | 3.45913300 | -0.01765600 |
| C | -1.23046900 | 2.78492300 | -0.02613600 |
| N | -1.40827000 | 1.41667500 | -0.03412900 |
| C | -0.00022100 | 3.44059800 | -0.02440300 |
| C | 1.23011500 | 2.78508100 | -0.02612800 |
| C | 2.50058300 | 3.45945200 | -0.01764200 |
| C | 3.46754200 | 2.48873500 | -0.00682700 |
| C | 2.79473900 | 1.22881400 | -0.01161800 |
| N | 1.40809000 | 1.41685600 | -0.03412700 |
| C | 3.43233000 | 0.00021700 | -0.00127300 |
| C | 2.79489400 | -1.22845700 | -0.01163400 |
| C | 3.46785800 | -2.48829400 | -0.00686800 |
| C | 2.50102600 | -3.45913200 | -0.01766700 |
| C | 1.23046800 | -2.78492400 | -0.02614900 |
| N | 1.40826800 | -1.41667500 | -0.03413900 |
| C | 0.00022100 | -3.44059900 | -0.02441600 |
| C | -1.23011400 | -2.78508200 | -0.02613900 |
| C | -2.50058300 | -3.45945200 | -0.01764000 |
| C | -3.46754100 | -2.48873500 | -0.00684500 |
| C | -2.79473800 | -1.22881400 | -0.01161900 |
| N | -1.40808800 | -1.41685600 | -0.03413300 |
| C | -3.43233000 | -0.00021700 | -0.00126700 |
| H | -4.54441300 | 2.60589300 | -0.00007800 |
| H | -2.62115600 | 4.53507000 | -0.02001000 |
| H | 2.62057600 | 4.53540400 | -0.01999300 |
| H | 4.54408100 | 2.60647100 | -0.00004600 |
| H | 4.54441200 | -2.60589200 | -0.00010000 |
| H | 2.62115600 | -4.53507000 | -0.02001900 |
| H | -2.62057600 | -4.53540400 | -0.01998400 |
| H | -4.54408000 | -2.60647000 | -0.00007300 |
| H | -0.00029100 | 4.52530100 | -0.01856300 |
| H | 4.51832800 | 0.00028500 | 0.01620100 |
| H | 0.00029100 | -4.52530200 | -0.01857400 |
| H | -4.51832800 | -0.00028700 | 0.01620600 |
| Co | 0.00000000 | 0.00000000 | 0.05078100 |
| H | 0.00000100 | -0.00000400 | 1.47203000 |

**[4]3dpt**

| | | | |
|---|---|---|---|
| C | -2.99967400 | -1.13744300 | -0.04454600 |
| C | -4.27743000 | -0.69064900 | -0.49719100 |
| C | -4.27743800 | 0.69064300 | -0.49716900 |
| C | -2.99967500 | 1.13743600 | -0.04454200 |
| N | -2.25516300 | -0.00000300 | 0.24671600 |
| C | -2.47818800 | 2.43003800 | -0.02590000 |
| C | -1.12728300 | 2.81626700 | 0.00904800 |
| C | -0.70518000 | 4.19803100 | -0.09160900 |
| C | 0.65763600 | 4.19793600 | -0.13243500 |
| C | 1.08301400 | 2.81597300 | -0.05988300 |
| N | -0.02109200 | 1.99619600 | 0.03395200 |
| C | 2.42712400 | 2.41400700 | -0.12534500 |
| C | 2.92367000 | 1.10576600 | -0.12043200 |
| C | 4.28917300 | 0.68381000 | -0.36487900 |
| C | 4.28918000 | -0.68380400 | -0.36484100 |
| C | 2.92367200 | -1.10576000 | -0.12043000 |
| N | 2.13991900 | 0.00000300 | 0.05199700 |
| C | 2.42712800 | -2.41400200 | -0.12534100 |
| C | 1.08301900 | -2.81597000 | -0.05988200 |
| C | 0.65764500 | -4.19793600 | -0.13241200 |
| C | -0.70517300 | -4.19803100 | -0.09163400 |
| C | -1.12727900 | -2.81626900 | 0.00904600 |
| N | -0.02109000 | -1.99619600 | 0.03395300 |
| C | -2.47818400 | -2.43004300 | -0.02590500 |
| H | -5.06352300 | -1.34498700 | -0.84967400 |
| H | -5.06353700 | 1.34498200 | -0.84963500 |
| H | -1.37913800 | 5.04361300 | -0.14037400 |
| H | 1.32730900 | 5.04382000 | -0.21985500 |
| H | 5.12293400 | 1.35149700 | -0.53945300 |
| H | 5.12294800 | -1.35149100 | -0.53938600 |
| H | 1.32732100 | -5.04381900 | -0.21981400 |
| H | -1.37912900 | -5.04361300 | -0.14041800 |
| H | -3.19568700 | 3.23359400 | -0.16147400 |
| H | 3.15535900 | 3.21112300 | -0.24163700 |
| H | 3.15536600 | -3.21111700 | -0.24161800 |
| H | -3.19568100 | -3.23360000 | -0.16149100 |
| Co | 0.21128900 | 0.00000000 | 0.58743100 |
| H | -0.34860200 | -0.00000200 | 2.18905700 |
| H | -1.70328000 | -0.00000500 | 1.12650100 |

**$^{2}$3-$CO_2$**

| | | | |
|---|---|---|---|
| C | 1.95699100 | -2.34262900 | -0.30617100 |
| C | 3.37747300 | -2.63212100 | -0.31888500 |
| C | 4.02602000 | -1.43961600 | -0.32582000 |
| C | 3.01197600 | -0.40374500 | -0.31633800 |
| N | 1.74583800 | -0.97155000 | -0.32919800 |
| C | 3.29311700 | 0.94160100 | -0.28427700 |
| C | 2.33025200 | 1.96807000 | -0.26817000 |
| C | 2.63315500 | 3.35711400 | -0.25155000 |
| C | 1.42250500 | 4.02873800 | -0.24577100 |
| C | 0.40479600 | 3.03596500 | -0.25943000 |
| N | 0.95926600 | 1.76563100 | -0.27902200 |
| C | -0.97658800 | 3.30530300 | -0.26716200 |
| C | -1.95716200 | 2.34240000 | -0.30591800 |
| C | -3.37765700 | 2.63186000 | -0.31877700 |
| C | -4.02614700 | 1.43931000 | -0.32598400 |
| C | -3.01205100 | 0.40347000 | -0.31634400 |
| N | -1.74599000 | 0.97142200 | -0.32880400 |
| C | -3.29306000 | -0.94194600 | -0.28430800 |
| C | -2.33015900 | -1.96841200 | -0.26769500 |
| C | -2.63318600 | -3.35745800 | -0.25158200 |
| C | -1.42260500 | -4.02921700 | -0.24580500 |
| C | -0.40481800 | -3.03652000 | -0.25898600 |
| N | -0.95912500 | -1.76618100 | -0.27808900 |
| C | 0.97659500 | -3.30568100 | -0.26709600 |
| H | 3.79572800 | -3.63123000 | -0.32133000 |
| H | 5.09211900 | -1.24825100 | -0.33605600 |
| H | 3.63440500 | 3.77016800 | -0.25343000 |
| H | 1.24218500 | 5.09669800 | -0.24048900 |
| H | -3.79597900 | 3.63094100 | -0.32114900 |
| H | -5.09224200 | 1.24793200 | -0.33645500 |
| H | -3.63446800 | -3.77043600 | -0.25383400 |
| H | -1.24239800 | -5.09719600 | -0.24087400 |
| H | 4.33744600 | 1.23932400 | -0.27438700 |
| H | -1.28518500 | 4.34616100 | -0.24994800 |
| H | -4.33736800 | -1.23976100 | -0.27486600 |
| H | 1.28540400 | -4.34647800 | -0.25006300 |
| Co | 0.00005200 | -0.00010600 | -0.11509000 |
| C | 0.00022300 | 0.00120400 | 1.82817900 |
| O | -1.11051600 | -0.18743600 | 2.38180300 |
| O | 1.11064000 | 0.19038400 | 2.38237900 |

**$^{2}$3pt-$CO_2$**

| | | | |
|---|---|---|---|
| C | 2.92977000 | 1.14160500 | -0.29361700 |
| C | 4.24489800 | 0.68337900 | -0.62916500 |
| C | 4.24491400 | -0.68337000 | -0.62916000 |
| C | 2.92979800 | -1.14162400 | -0.29360500 |
| N | 2.10111400 | -0.00002200 | -0.03313600 |
| C | 2.46804900 | -2.42286500 | -0.22612400 |
| C | 1.11309500 | -2.81597600 | -0.12188500 |
| C | 0.70344800 | -4.17842600 | -0.05619400 |
| C | -0.67381800 | -4.18912100 | -0.09206700 |
| C | -1.09008600 | -2.82818100 | -0.18924100 |
| N | 0.00575900 | -1.98320400 | -0.19144800 |
| C | -2.42273900 | -2.41159200 | -0.32390700 |
| C | -2.83243300 | -1.10270700 | -0.46203000 |
| C | -4.21716300 | -0.67920200 | -0.50777700 |
| C | -4.21717900 | 0.67903000 | -0.50780100 |
| C | -2.83245900 | 1.10257100 | -0.46206900 |
| N | -1.99669600 | -0.00005900 | -0.49857700 |
| C | -2.42279700 | 2.41147100 | -0.32399400 |
| C | -1.09015200 | 2.82809500 | -0.18934700 |
| C | -0.67391000 | 4.18904700 | -0.09222800 |
| C | 0.70335600 | 4.17838000 | -0.05634500 |
| C | 1.11303000 | 2.81593400 | -0.12197700 |
| N | 0.00571100 | 1.98314100 | -0.19152800 |
| C | 2.46799300 | 2.42284000 | -0.22617300 |
| H | 5.06624600 | 1.34231300 | -0.88007400 |
| H | 5.06627900 | -1.34228700 | -0.88006200 |
| H | 1.38251000 | -5.02008200 | -0.00423600 |
| H | -1.34374700 | -5.03921900 | -0.07982100 |
| H | -5.06168000 | -1.35660500 | -0.51698600 |
| H | -5.06171300 | 1.35641300 | -0.51703300 |
| H | -1.34385600 | 5.03913200 | -0.08001500 |
| H | 1.38240100 | 5.02005000 | -0.00441100 |
| H | 3.19727500 | -3.22139200 | -0.32595800 |
| H | -3.18831500 | -3.18088300 | -0.30506800 |
| H | -3.18838900 | 3.18074500 | -0.30518100 |
| H | 3.19720500 | 3.22137800 | -0.32602600 |
| Co | -0.07066100 | -0.00002900 | -0.14554100 |
| H | 1.83357700 | -0.00005900 | 0.99910200 |
| C | -0.17613000 | 0.00000800 | 1.79997100 |
| O | 0.97631000 | 0.00051700 | 2.34096000 |
| O | -1.28357000 | 0.00029800 | 2.35736600 |

**[1]4**

| | | | |
|---|---|---|---|
| C | 2.82576800 | -1.10105500 | -0.38407700 |
| C | 4.19703600 | -0.68357700 | -0.48569800 |
| C | 4.19779800 | 0.67968200 | -0.48427700 |
| C | 2.82697800 | 1.09847300 | -0.38203800 |
| N | 1.98371000 | -0.00086200 | -0.34745500 |
| C | 2.41606300 | 2.41634500 | -0.27662400 |
| C | 1.09371700 | 2.81659300 | -0.18632100 |
| C | 0.67749500 | 4.19204500 | -0.10285800 |
| C | -0.68392000 | 4.19319100 | -0.10227300 |
| C | -1.10165100 | 2.81846200 | -0.18643500 |
| N | -0.00555300 | 1.97844800 | -0.22713900 |
| C | -2.42305000 | 2.41508800 | -0.27445900 |
| C | -2.82941600 | 1.09646400 | -0.38735100 |
| C | -4.20815500 | 0.68267700 | -0.44544100 |
| C | -4.20889700 | -0.67834400 | -0.44408000 |
| C | -2.83060600 | -1.09352400 | -0.38543600 |
| N | -1.99139900 | 0.00100700 | -0.39612400 |
| C | -2.42557200 | -2.41244500 | -0.27123000 |
| C | -1.10448800 | -2.81717300 | -0.18469900 |
| C | -0.68804700 | -4.19232400 | -0.10108600 |
| C | 0.67336800 | -4.19262200 | -0.10456300 |
| C | 1.09088600 | -2.81759800 | -0.18858700 |
| N | -0.00757800 | -1.97831700 | -0.22745500 |
| C | 2.41353300 | -2.41863500 | -0.28020100 |
| H | 5.03742700 | -1.36289600 | -0.54052000 |
| H | 5.03894400 | 1.35817200 | -0.53774600 |
| H | 1.35866900 | 5.03204600 | -0.06793300 |
| H | -1.36360400 | 5.03437400 | -0.06705500 |
| H | -5.04809800 | 1.36432100 | -0.47232500 |
| H | -5.04958400 | -1.35911800 | -0.46967800 |
| H | -1.36854600 | -5.03278800 | -0.06447700 |
| H | 1.35371900 | -5.03334400 | -0.07098500 |
| H | 3.17978800 | 3.18655300 | -0.27106500 |
| H | -3.18944700 | 3.18239400 | -0.25274400 |
| H | -3.19276600 | -3.17890600 | -0.24794000 |
| H | 3.17646000 | -3.18964400 | -0.27623100 |
| Co | -0.01836300 | 0.00005900 | -0.16071000 |
| C | -0.08900200 | -0.00045700 | 1.72207000 |
| O | -1.10895400 | -0.00041000 | 2.37959300 |
| O | 1.10603600 | -0.00099200 | 2.36809900 |
| H | 1.81286800 | -0.00107800 | 1.69753100 |

**$^4$5**

| | | | |
|---|---|---|---|
| C | -2.66680200 | 1.48108500 | -0.42585500 |
| C | -4.09090100 | 1.23749500 | -0.52352300 |
| C | -4.26154100 | -0.11869700 | -0.54275900 |
| C | -2.94195300 | -0.70983600 | -0.45846500 |
| N | -1.99507800 | 0.28367900 | -0.38468900 |
| C | -2.66527800 | -2.08267900 | -0.46237100 |
| C | -1.40181600 | -2.68684500 | -0.44459400 |
| C | -1.16203500 | -4.11612700 | -0.50318500 |
| C | 0.19232000 | -4.28997100 | -0.48484300 |
| C | 0.78299400 | -2.96729000 | -0.41480700 |
| N | -0.20616400 | -2.01860600 | -0.39161800 |
| C | 2.15788600 | -2.69963600 | -0.38346100 |
| C | 2.76430900 | -1.43927600 | -0.33985300 |
| C | 4.19080800 | -1.19131100 | -0.37129400 |
| C | 4.35798900 | 0.16440700 | -0.33587700 |
| C | 3.03426800 | 0.75155700 | -0.28340100 |
| N | 2.09073200 | -0.24472600 | -0.27919500 |
| C | 2.75979300 | 2.12566100 | -0.26973800 |
| C | 1.49844100 | 2.73516600 | -0.29081800 |
| C | 1.25979600 | 4.16337100 | -0.35333200 |
| C | -0.09470800 | 4.33846200 | -0.39142900 |
| C | -0.69045300 | 3.01820700 | -0.35202200 |
| N | 0.29903500 | 2.06943200 | -0.28475100 |
| C | -2.06256500 | 2.74510800 | -0.39686900 |
| H | -4.84890000 | 2.00876800 | -0.58003900 |
| H | -5.18676000 | -0.67640400 | -0.61653200 |
| H | -1.93491600 | -4.87253000 | -0.56060700 |
| H | 0.75059400 | -5.21700200 | -0.52362600 |
| H | 4.95220000 | -1.95934300 | -0.42561200 |
| H | 5.28377700 | 0.72575600 | -0.35362900 |
| H | 2.03407500 | 4.92013100 | -0.37572100 |
| H | -0.64903400 | 5.26663100 | -0.45202000 |
| H | -3.52150800 | -2.74947100 | -0.51110100 |
| H | 2.82206200 | -3.55859200 | -0.41644300 |
| H | 3.61878100 | 2.79059500 | -0.27709200 |
| H | -2.72820800 | 3.60246500 | -0.44039500 |
| Co | 0.07091000 | 0.03949700 | 0.09662500 |
| C | -0.17873000 | -0.15843600 | 2.16213000 |
| O | 0.68492400 | -0.24596300 | 3.04414500 |
| O | -1.49213000 | -0.21231600 | 2.65921500 |
| H | -2.05969000 | -0.11210700 | 1.87700700 |

**$^{2}$6**

| | | | |
|---|---|---|---|
| C | -1.28337500 | 2.77928200 | -0.15638400 |
| C | -2.55484200 | 3.46055000 | -0.14240000 |
| C | -3.51693800 | 2.49639500 | -0.13307000 |
| C | -2.83310100 | 1.22628600 | -0.13765200 |
| N | -1.46841700 | 1.41427400 | -0.15588200 |
| C | -3.47477200 | -0.00455700 | -0.12349400 |
| C | -2.82982700 | -1.23369100 | -0.13767400 |
| C | -3.51027600 | -2.50561900 | -0.13309300 |
| C | -2.54562300 | -3.46721500 | -0.14252600 |
| C | -1.27597600 | -2.78256400 | -0.15648100 |
| N | -1.46464600 | -1.41805400 | -0.15592600 |
| C | -0.04505500 | -3.42441800 | -0.17685300 |
| C | 1.18335300 | -2.78080100 | -0.23013400 |
| C | 2.45433400 | -3.46079700 | -0.26901900 |
| C | 3.41595100 | -2.49651600 | -0.31085400 |
| C | 2.73352400 | -1.22637300 | -0.29616400 |
| N | 1.36869000 | -1.41518700 | -0.25814200 |
| C | 3.37473800 | 0.00455100 | -0.31055400 |
| C | 2.73024300 | 1.23376400 | -0.29599300 |
| C | 3.40929200 | 2.50572000 | -0.31052800 |
| C | 2.44512000 | 3.46744300 | -0.26866500 |
| C | 1.17595200 | 2.78406500 | -0.22988000 |
| N | 1.36491200 | 1.41895200 | -0.25796600 |
| C | -0.05416200 | 3.42441300 | -0.17665800 |
| H | -2.67188200 | 4.53645000 | -0.14629900 |
| H | -4.59307600 | 2.61114100 | -0.12683000 |
| H | -4.58610600 | -2.62323100 | -0.12682500 |
| H | -2.65979900 | -4.54342100 | -0.14648700 |
| H | 2.57186700 | -4.53664400 | -0.26722800 |
| H | 4.49132500 | -2.61185200 | -0.35003700 |
| H | 4.48435900 | 2.62391600 | -0.34965500 |
| H | 2.55979100 | 4.54359800 | -0.26679200 |
| H | -4.55973600 | -0.00599900 | -0.10788100 |
| H | -0.04350400 | -4.50941000 | -0.16431200 |
| H | 4.45943500 | 0.00599800 | -0.33686000 |
| H | -0.05549300 | 4.50940500 | -0.16407400 |
| Co | -0.04606800 | -0.00001200 | -0.07761400 |
| C | 0.30624400 | 0.00008300 | 1.97224200 |
| O | 0.92675600 | 0.00005800 | 2.92991000 |

**[1]TS1**

| | | | |
|---|---|---|---|
| C | -1.22641800 | 2.77881300 | -0.34506400 |
| C | -2.49781300 | 3.46139300 | -0.31718800 |
| C | -3.45797700 | 2.49723300 | -0.26961200 |
| C | -2.77146200 | 1.22765900 | -0.27698200 |
| N | -1.40030500 | 1.40599900 | -0.32663300 |
| C | -3.41482000 | 0.00000800 | -0.24674200 |
| C | -2.77145900 | -1.22764100 | -0.27699100 |
| C | -3.45797000 | -2.49721600 | -0.26961000 |
| C | -2.49780400 | -3.46137500 | -0.31718400 |
| C | -1.22641100 | -2.77879100 | -0.34507300 |
| N | -1.40030200 | -1.40597700 | -0.32665300 |
| C | -0.00006000 | -3.42496400 | -0.36337500 |
| C | 1.22628500 | -2.77878700 | -0.34487100 |
| C | 2.49767700 | -3.46136600 | -0.31690600 |
| C | 3.45783800 | -2.49720600 | -0.26925500 |
| C | 2.77132600 | -1.22763200 | -0.27665100 |
| N | 1.40016800 | -1.40596800 | -0.32638700 |
| C | 3.41468500 | 0.00001600 | -0.24634500 |
| C | 2.77132400 | 1.22766300 | -0.27664200 |
| C | 3.45783300 | 2.49723900 | -0.26925700 |
| C | 2.49767000 | 3.46139600 | -0.31691000 |
| C | 1.22627900 | 2.77881500 | -0.34486300 |
| N | 1.40016500 | 1.40599600 | -0.32636700 |
| C | -0.00006800 | 3.42498900 | -0.36337100 |
| H | -2.61534400 | 4.53770000 | -0.32734500 |
| H | -4.53420800 | 2.61120600 | -0.23588400 |
| H | -4.53420100 | -2.61119300 | -0.23587500 |
| H | -2.61533200 | -4.53768200 | -0.32733100 |
| H | 2.61521000 | -4.53767200 | -0.32707700 |
| H | 4.53406700 | -2.61118000 | -0.23545700 |
| H | 4.53406200 | 2.61121600 | -0.23546800 |
| H | 2.61520000 | 4.53770300 | -0.32709200 |
| H | -4.49987800 | 0.00000600 | -0.20601800 |
| H | -0.00005700 | -4.51068400 | -0.37430200 |
| H | 4.49974100 | 0.00001700 | -0.20556500 |
| H | -0.00006700 | 4.51070900 | -0.37431000 |
| Co | -0.00008100 | 0.00000900 | -0.31005200 |
| C | 0.00060700 | -0.00009800 | 2.48960800 |
| O | 1.17017700 | -0.00010300 | 2.64436800 |
| O | -1.16901100 | -0.00010200 | 2.64376900 |

**$^{3}$TS2**

| | | | |
|---|---|---|---|
| C | -2.73855900 | -1.12816500 | -0.67869600 |
| C | -4.06259200 | -0.67854700 | -1.01283600 |
| C | -4.06170100 | 0.68455700 | -1.01227400 |
| C | -2.73705400 | 1.13217900 | -0.67787000 |
| N | -1.91903200 | 0.00136100 | -0.38721500 |
| C | -2.30699800 | 2.42880600 | -0.63135400 |
| C | -0.96612600 | 2.85134400 | -0.47811900 |
| C | -0.56030600 | 4.21422200 | -0.41469300 |
| C | 0.81862300 | 4.21631100 | -0.32658300 |
| C | 1.23244900 | 2.85228100 | -0.34675000 |
| N | 0.13571800 | 2.01594100 | -0.43281500 |
| C | 2.57154400 | 2.41885500 | -0.30559600 |
| C | 2.98338700 | 1.10290700 | -0.33698900 |
| C | 4.36573400 | 0.67631900 | -0.23756400 |
| C | 4.36479300 | -0.68216200 | -0.23753500 |
| C | 2.98185900 | -1.10684000 | -0.33696000 |
| N | 2.15383300 | -0.00139700 | -0.42653400 |
| C | 2.56817900 | -2.42221000 | -0.30554800 |
| C | 1.22848100 | -2.85375400 | -0.34683300 |
| C | 0.81276600 | -4.21720700 | -0.32681600 |
| C | -0.56613700 | -4.21321200 | -0.41529600 |
| C | -0.97006100 | -2.84977700 | -0.47876900 |
| N | 0.13292700 | -2.01588500 | -0.43311100 |
| C | -2.31030400 | -2.42539400 | -0.63245900 |
| H | -4.88358600 | -1.34263900 | -1.25258600 |
| H | -4.88182500 | 1.34991400 | -1.25149500 |
| H | -1.23581000 | 5.06038600 | -0.43287900 |
| H | 1.48937700 | 5.06453400 | -0.27025800 |
| H | 5.20895000 | 1.35200700 | -0.16556700 |
| H | 5.20707600 | -1.35901200 | -0.16552500 |
| H | 1.48233900 | -5.06635600 | -0.27038500 |
| H | -1.24279900 | -5.05844400 | -0.43372100 |
| H | -3.05129800 | 3.20647200 | -0.77656800 |
| H | 3.33838800 | 3.18401000 | -0.23207600 |
| H | 3.33394400 | -3.18844200 | -0.23199600 |
| H | -3.05560800 | -3.20201100 | -0.77813300 |
| Co | 0.16138900 | 0.00001700 | -0.38824600 |
| H | -1.87166700 | 0.00055100 | 0.88946500 |
| O | -1.94188200 | -0.00039100 | 2.11632300 |
| C | -0.75993900 | -0.00011500 | 2.62450400 |
| O | -0.77550100 | -0.00035400 | 3.98409800 |
| O | 0.31803800 | 0.00027900 | 2.00149100 |
| H | 0.14742800 | -0.00024100 | 4.28701400 |

**[1]TS3**

| | | | |
|---|---|---|---|
| C | 1.24027300 | 2.84339700 | -0.35591800 |
| C | 2.45752600 | 3.60025300 | -0.19334600 |
| C | 3.46945400 | 2.69551700 | -0.09025500 |
| C | 2.86764700 | 1.38803300 | -0.18664800 |
| N | 1.50017200 | 1.48671700 | -0.35633500 |
| C | 3.57932200 | 0.20111200 | -0.10711900 |
| C | 3.01096000 | -1.06119700 | -0.17830400 |
| C | 3.76100100 | -2.28894600 | -0.07321200 |
| C | 2.86088400 | -3.30600100 | -0.16577900 |
| C | 1.56349900 | -2.69714400 | -0.33029400 |
| N | 1.66411800 | -1.31955800 | -0.34426600 |
| C | 0.38306200 | -3.41283600 | -0.45726700 |
| C | -0.86711900 | -2.83972500 | -0.62976500 |
| C | -2.08708600 | -3.59656100 | -0.77199100 |
| C | -3.09246600 | -2.69161500 | -0.92429800 |
| C | -2.48491100 | -1.38428000 | -0.87100400 |
| N | -1.11858000 | -1.48280800 | -0.69605700 |
| C | -3.19406900 | -0.19812700 | -0.97545600 |
| C | -2.62639500 | 1.06379600 | -0.89697500 |
| C | -3.38046500 | 2.29139800 | -0.97141300 |
| C | -2.48712800 | 3.30838600 | -0.82760800 |
| C | -1.18864400 | 2.69961000 | -0.67002900 |
| N | -1.28129100 | 1.32219000 | -0.72020200 |
| C | -0.01448200 | 3.41569600 | -0.49673400 |
| H | 2.50657800 | 4.68129600 | -0.16682600 |
| H | 4.52938600 | 2.87248000 | 0.04024500 |
| H | 4.83447600 | -2.34047200 | 0.05613900 |
| H | 3.03512000 | -4.37378100 | -0.13020700 |
| H | -2.14146000 | -4.67756000 | -0.75652700 |
| H | -4.15174900 | -2.86823300 | -1.06046000 |
| H | -4.45237100 | 2.34281300 | -1.11327300 |
| H | -2.66608000 | 4.37598100 | -0.82660300 |
| H | 4.65430600 | 0.26447000 | 0.02862400 |
| H | 0.44251100 | -4.49608300 | -0.42451200 |
| H | -4.26889300 | -0.26169300 | -1.11198700 |
| H | -0.08137300 | 4.49880200 | -0.47548500 |
| Co | 0.18363700 | 0.00202800 | -0.48621600 |
| H | 0.02161000 | -0.00325700 | 1.04006200 |
| O | 0.04866300 | -0.01452300 | 2.40513100 |
| C | -1.14093200 | -0.00862800 | 2.90155200 |
| O | -1.11320500 | -0.02167200 | 4.27952700 |
| O | -2.22463300 | 0.00705600 | 2.30282600 |
| H | -2.03738100 | -0.01503600 | 4.57797100 |

**$^{2}$TS4**

| | | | |
|---|---|---|---|
| C | 2.46484700 | -1.72942700 | -0.38082200 |
| C | 3.91554400 | -1.70624600 | -0.38015300 |
| C | 4.29295700 | -0.40367500 | -0.29863500 |
| C | 3.07798000 | 0.38842800 | -0.24609800 |
| N | 1.96150100 | -0.43582100 | -0.30797600 |
| C | 3.05503700 | 1.76109400 | -0.13206800 |
| C | 1.88733600 | 2.54910900 | -0.08270900 |
| C | 1.87219300 | 3.96897600 | 0.02241700 |
| C | 0.54179400 | 4.35413400 | 0.02609600 |
| C | -0.22940400 | 3.16271400 | -0.08082800 |
| N | 0.59427900 | 2.04351200 | -0.14050400 |
| C | -1.63622700 | 3.12045900 | -0.14050800 |
| C | -2.38849200 | 1.97429100 | -0.26594600 |
| C | -3.83883300 | 1.95060100 | -0.28448500 |
| C | -4.21420800 | 0.64800900 | -0.37440900 |
| C | -2.99851700 | -0.14362800 | -0.41290400 |
| N | -1.88255400 | 0.68254000 | -0.36594800 |
| C | -2.97767100 | -1.52047500 | -0.46372300 |
| C | -1.81390200 | -2.31501200 | -0.46159300 |
| C | -1.79969700 | -3.73821400 | -0.50442300 |
| C | -0.46905700 | -4.12431100 | -0.49807400 |
| C | 0.30401100 | -2.93016300 | -0.44928000 |
| N | -0.51996800 | -1.81056900 | -0.42617100 |
| C | 1.71184300 | -2.88149200 | -0.43217900 |
| H | 4.54169400 | -2.58890100 | -0.43607600 |
| H | 5.29438700 | 0.00947500 | -0.27206500 |
| H | 2.75508400 | 4.59393600 | 0.08742800 |
| H | 0.12969500 | 5.35473800 | 0.08347700 |
| H | -4.46601500 | 2.83230800 | -0.22575400 |
| H | -5.21512400 | 0.23392100 | -0.40601000 |
| H | -2.68281600 | -4.36491700 | -0.54451000 |
| H | -0.05826000 | -5.12678800 | -0.52234500 |
| H | 4.00846400 | 2.28047500 | -0.08522400 |
| H | -2.16571600 | 4.06726200 | -0.07459200 |
| H | -3.93270300 | -2.03825100 | -0.49329400 |
| H | 2.24241800 | -3.82915700 | -0.47143400 |
| Co | 0.03542000 | 0.11245800 | -0.27169100 |
| C | -0.30614000 | -0.95704400 | 2.34687100 |
| O | -1.45581900 | -0.73592800 | 2.51500800 |
| O | 0.80724000 | -1.31242800 | 2.52761200 |

**$^{3}$TS5**

| | | | |
|---|---|---|---|
| C | -2.64936400 | -1.71815800 | -0.29761100 |
| C | -3.99382000 | -1.56599200 | -0.70904800 |
| C | -4.32993800 | -0.22020000 | -0.63956100 |
| C | -3.20121400 | 0.50060400 | -0.18149200 |
| N | -2.16579300 | -0.43518700 | 0.04459400 |
| C | -3.02189600 | 1.87743000 | -0.05583600 |
| C | -1.80761300 | 2.57498000 | 0.00773500 |
| C | -1.72187400 | 4.02003000 | 0.06742100 |
| C | -0.39972200 | 4.34493500 | 0.01355200 |
| C | 0.34180600 | 3.10587400 | -0.09202600 |
| N | -0.53765000 | 2.03799300 | -0.07667500 |
| C | 1.72932600 | 3.03021200 | -0.23041200 |
| C | 2.50673900 | 1.87243800 | -0.38950600 |
| C | 3.94833700 | 1.80086200 | -0.43423400 |
| C | 4.27917700 | 0.47243500 | -0.51661500 |
| C | 3.03851500 | -0.26623800 | -0.51849000 |
| N | 1.98701500 | 0.60782400 | -0.46972100 |
| C | 2.89357300 | -1.66322200 | -0.51500800 |
| C | 1.70470900 | -2.38411500 | -0.41897100 |
| C | 1.62171700 | -3.83275200 | -0.43223700 |
| C | 0.30272200 | -4.15956200 | -0.37046100 |
| C | -0.44623100 | -2.91810700 | -0.30354700 |
| N | 0.43218600 | -1.84848000 | -0.31730400 |
| C | -1.84246500 | -2.85752900 | -0.31876600 |
| H | -4.61302100 | -2.37155000 | -1.08151000 |
| H | -5.26236200 | 0.23445500 | -0.94671700 |
| H | -2.57517800 | 4.68245400 | 0.13469600 |
| H | 0.04915200 | 5.33008200 | 0.02134300 |
| H | 4.61443400 | 2.65334800 | -0.39284000 |
| H | 5.26663200 | 0.03048500 | -0.55315600 |
| H | 2.47546000 | -4.49449300 | -0.50194200 |
| H | -0.14643400 | -5.14446100 | -0.37334000 |
| H | -3.92020500 | 2.48459000 | -0.11276800 |
| H | 2.26530000 | 3.97453200 | -0.20127800 |
| H | 3.80693100 | -2.24808000 | -0.57124300 |
| H | -2.35934200 | -3.80381900 | -0.44861000 |
| Co | 0.05316500 | 0.11925500 | -0.17900600 |
| H | -1.78394200 | -0.39957300 | 0.99873100 |
| C | 0.40197800 | -0.80714500 | 2.19178600 |
| O | -0.74820100 | -0.93357600 | 2.52305000 |
| O | 1.57445800 | -0.80687300 | 2.40888800 |

**[1]TS6**

| | | | |
|---|---|---|---|
| C | 1.59066600 | 1.85852900 | -1.59145300 |
| C | 1.93375400 | 3.25241700 | -1.71649900 |
| C | 0.86265000 | 3.96111700 | -1.26493700 |
| C | -0.13064800 | 2.99870000 | -0.85988200 |
| N | 0.31708200 | 1.71288900 | -1.08107200 |
| C | -1.35309900 | 3.33962700 | -0.30493200 |
| C | -2.30607300 | 2.42762900 | 0.11803400 |
| C | -3.56652400 | 2.80066600 | 0.70844600 |
| C | -4.21775200 | 1.64305300 | 1.00776000 |
| C | -3.35536100 | 0.56460400 | 0.59706600 |
| N | -2.19053500 | 1.05491900 | 0.04232600 |
| C | -3.67155700 | -0.77652400 | 0.73651700 |
| C | -2.85347000 | -1.81454400 | 0.32268100 |
| C | -3.19590700 | -3.20824300 | 0.44761500 |
| C | -2.14960200 | -3.91585500 | -0.06058200 |
| C | -1.16748600 | -2.95383800 | -0.49111700 |
| N | -1.61340600 | -1.66725800 | -0.26630200 |
| C | 0.05859200 | -3.29601300 | -1.03684700 |
| C | 1.02221400 | -2.38446400 | -1.43470300 |
| C | 2.30758400 | -2.75694500 | -1.96873900 |
| C | 2.98045500 | -1.59920600 | -2.21453700 |
| C | 2.10307100 | -0.52131800 | -1.83547000 |
| N | 0.90038300 | -1.01170000 | -1.36866400 |
| C | 2.43592900 | 0.81873700 | -1.94050100 |
| H | 2.87623100 | 3.61606900 | -2.10453500 |
| H | 0.73389600 | 5.03376000 | -1.20130200 |
| H | -3.88977500 | 3.82222400 | 0.86047500 |
| H | -5.19166600 | 1.50674800 | 1.45948200 |
| H | -4.12374700 | -3.57277800 | 0.86868700 |
| H | -2.03092500 | -4.98808600 | -0.14680100 |
| H | 2.62960300 | -3.77804600 | -2.12633200 |
| H | 3.97536700 | -1.46220400 | -2.61762900 |
| H | -1.57606500 | 4.39452500 | -0.18395200 |
| H | -4.62353500 | -1.02976300 | 1.19094700 |
| H | 0.28466100 | -4.35121700 | -1.14897600 |
| H | 3.41965900 | 1.06938600 | -2.32276800 |
| Co | -0.58841700 | 0.01816800 | -0.53307400 |
| C | 0.19575000 | 0.01581100 | 1.19038400 |
| O | 0.65658900 | -1.14057100 | 1.58775500 |
| O | 0.27976800 | 1.05892600 | 1.83779100 |

**$^{4}$TS7**

| | | | |
|---|---|---|---|
| C | 2.88356600 | 1.43485200 | -0.30617100 |
| C | 4.26239600 | 1.10183400 | -0.31888500 |
| C | 4.40701400 | -0.25819800 | -0.32582000 |
| C | 3.12885700 | -0.86412000 | -0.31633800 |
| N | 2.19213100 | 0.20249500 | -0.32919800 |
| C | 2.77642400 | -2.18487000 | -0.28427700 |
| C | 1.46417600 | -2.75165800 | -0.26817000 |
| C | 1.19700100 | -4.14568600 | -0.25155000 |
| C | -0.18144100 | -4.29523200 | -0.24577100 |
| C | -0.73645400 | -2.98599400 | -0.25943000 |
| N | 0.27578200 | -2.04849100 | -0.27902200 |
| C | -2.12572200 | -2.69464500 | -0.26716200 |
| C | -2.71151800 | -1.44683900 | -0.30591800 |
| C | -4.14453500 | -1.16573600 | -0.31877700 |
| C | -4.28781300 | 0.18423100 | -0.32598400 |
| C | -2.94585000 | 0.76019800 | -0.31634400 |
| N | -2.02071800 | -0.25815600 | -0.32880400 |
| C | -2.63711800 | 2.10505000 | -0.28430800 |
| C | -1.34394900 | 2.69119400 | -0.26769500 |
| C | -1.07903300 | 4.08855500 | -0.25158200 |
| C | 0.30033600 | 4.23646600 | -0.24580500 |
| C | 0.85794600 | 2.93121600 | -0.25898600 |
| N | -0.15339800 | 1.99159300 | -0.27808900 |
| C | 2.26148200 | 2.65176100 | -0.26709600 |
| H | 5.02752300 | 1.83324600 | -0.32133000 |
| H | 5.30980700 | -0.82016500 | -0.33605600 |
| H | 1.94953500 | -4.92416800 | -0.25343000 |
| H | -0.75208600 | -5.21626600 | -0.24048900 |
| H | -4.92245400 | -1.92043200 | -0.32114900 |
| H | -5.20654800 | 0.75892100 | -0.33645500 |
| H | -1.83162300 | 4.86696500 | -0.25383400 |
| H | 0.87053900 | 5.15811800 | -0.24087400 |
| H | 3.59228800 | -2.90091000 | -0.27438700 |
| H | -2.79201000 | -3.55400500 | -0.24994800 |
| H | -3.47113500 | 2.80264300 | -0.27486600 |
| H | 2.90876400 | 3.52003600 | -0.25006300 |
| Co | -0.05637400 | -0.03852200 | -0.11509000 |
| H | 1.78291100 | 0.17622300 | 1.82817900 |
| C | -0.51167900 | 0.23844000 | 2.38180300 |
| O | 0.65949300 | 0.16541300 | 2.38237900 |
| O | -1.66005800 | 0.36744700 | 2.48969000 |

**[2]TS8**

| | | | |
|---|---|---|---|
| C | -2.97925900 | -1.13858200 | -0.39318400 |
| C | -4.25672300 | -0.69020100 | -0.84960700 |
| C | -4.25667900 | 0.69010000 | -0.84942300 |
| C | -2.97918100 | 1.13832100 | -0.39290300 |
| N | -2.23755600 | -0.00019000 | -0.09997200 |
| C | -2.45691200 | 2.42895800 | -0.37052800 |
| C | -1.10357300 | 2.81251500 | -0.32553600 |
| C | -0.68048600 | 4.19656500 | -0.38459000 |
| C | 0.68294700 | 4.19606600 | -0.40914900 |
| C | 1.10467100 | 2.81147600 | -0.36704900 |
| N | -0.00139600 | 1.99001100 | -0.31561800 |
| C | 2.45040800 | 2.41176700 | -0.40856800 |
| C | 2.94876100 | 1.10439500 | -0.40027000 |
| C | 4.32345300 | 0.68356600 | -0.60056500 |
| C | 4.32347900 | -0.68355900 | -0.60037700 |
| C | 2.94879800 | -1.10437900 | -0.39998900 |
| N | 2.15893500 | 0.00001200 | -0.25703700 |
| C | 2.45045900 | -2.41177300 | -0.40805000 |
| C | 1.10472600 | -2.81152500 | -0.36665800 |
| C | 0.68308400 | -4.19614300 | -0.40878600 |
| C | -0.68036000 | -4.19671700 | -0.38451600 |
| C | -1.10354500 | -2.81269200 | -0.32557500 |
| N | -0.00139800 | -1.99012900 | -0.31541700 |
| C | -2.45694100 | -2.42923100 | -0.37081300 |
| H | -5.04116500 | -1.34443100 | -1.20620700 |
| H | -5.04106700 | 1.34447100 | -1.20588700 |
| H | -1.35306800 | 5.04422200 | -0.41645600 |
| H | 1.35459300 | 5.04341400 | -0.46157700 |
| H | 5.16291000 | 1.35154500 | -0.74496300 |
| H | 5.16295900 | -1.35154800 | -0.74459600 |
| H | 1.35479300 | -5.04345000 | -0.46105400 |
| H | -1.35289300 | -5.04441000 | -0.41648400 |
| H | -3.17314800 | 3.23447500 | -0.50158500 |
| H | 3.18003500 | 3.21172200 | -0.49476500 |
| H | 3.18010800 | -3.21173000 | -0.49404300 |
| H | -3.17309500 | -3.23479800 | -0.50199700 |
| Co | 0.22915500 | -0.00000700 | 0.27644400 |
| H | -1.67421900 | -0.00045300 | 0.76956200 |
| C | -0.17324900 | 0.00039200 | 2.30067100 |
| O | -0.22265600 | -1.12414200 | 2.91424300 |
| O | -0.22325700 | 1.12505700 | 2.91390400 |

**²TS9**

| | | | |
|---|---|---|---|
| C | 1.27012400 | 2.99755000 | -0.30862200 |
| C | 0.63867600 | 4.28307600 | -0.48968700 |
| C | -0.63778600 | 4.04003200 | -0.89939700 |
| C | -0.78734700 | 2.60572100 | -0.96470300 |
| N | 0.38799600 | 1.98186400 | -0.60413800 |
| C | -1.96215800 | 1.95482000 | -1.32328300 |
| C | -2.12298400 | 0.57556500 | -1.37155700 |
| C | -3.35927600 | -0.08326700 | -1.72287800 |
| C | -3.13634700 | -1.42200400 | -1.60833500 |
| C | -1.75988500 | -1.58045400 | -1.20027200 |
| N | -1.15311900 | -0.35168900 | -1.06531300 |
| C | -1.14154300 | -2.80763000 | -0.99945000 |
| C | 0.19707800 | -2.97996400 | -0.66773000 |
| C | 0.83702100 | -4.26547600 | -0.51637100 |
| C | 2.14019200 | -4.02017200 | -0.20365800 |
| C | 2.29314700 | -2.58510500 | -0.15691600 |
| N | 1.10059900 | -1.96454700 | -0.45258000 |
| C | 3.47914600 | -1.93560600 | 0.16272300 |
| C | 3.63645800 | -0.55716400 | 0.23669200 |
| C | 4.87222000 | 0.10177200 | 0.58906300 |
| C | 4.61856600 | 1.44022200 | 0.58134700 |
| C | 3.22978300 | 1.59774700 | 0.21763500 |
| N | 2.64554900 | 0.36988400 | 0.00618000 |
| C | 2.58978300 | 2.82481100 | 0.09022400 |
| H | 1.12860300 | 5.23431600 | -0.32587000 |
| H | -1.41951400 | 4.74855100 | -1.14113000 |
| H | -4.26928900 | 0.43210900 | -2.00062800 |
| H | -3.82253800 | -2.24085400 | -1.78084500 |
| H | 0.33742900 | -5.21715600 | -0.64447300 |
| H | 2.93809800 | -4.72765700 | -0.01802200 |
| H | 5.79909100 | -0.41209600 | 0.80953500 |
| H | 5.29334900 | 2.25995900 | 0.79198600 |
| H | -2.82079900 | 2.56912500 | -1.57329300 |
| H | -1.74296700 | -3.70048800 | -1.13724400 |
| H | 4.34669500 | -2.55166300 | 0.37670100 |
| H | 3.17259300 | 3.71749900 | 0.29374400 |
| Co | 0.70288700 | 0.00397300 | -0.37050600 |
| C | 0.15561800 | -0.13852600 | 1.67081500 |
| O | 0.35679000 | -0.83559800 | 2.60899100 |
| O | -1.15249900 | 0.95666700 | 2.08851900 |
| H | -1.21539300 | 1.48614800 | 1.27836600 |
| C | -4.09564300 | -0.01864800 | 1.41649400 |
| O | -3.02131800 | -0.60053700 | 1.87121200 |

| | | | |
|---|---|---|---|
| O | -5.08697100 | -0.92861300 | 1.21769500 |
| O | -4.25099200 | 1.18168300 | 1.19184400 |
| H | -2.20353800 | 0.13768000 | 1.99030900 |
| H | -5.85609200 | -0.44392700 | 0.87659300 |

**$^{4}$TS10**

| | | | |
|---|---|---|---|
| C | -2.16437600 | -1.22230900 | -1.17244700 |
| C | -3.24668200 | -0.83167900 | -2.01825000 |
| C | -3.28420600 | 0.54367200 | -2.05183200 |
| C | -2.22979600 | 1.03329200 | -1.22303100 |
| N | -1.58509400 | -0.06421200 | -0.61502500 |
| C | -1.81968500 | 2.35351100 | -1.08730900 |
| C | -0.56541500 | 2.81352900 | -0.64559700 |
| C | -0.18171700 | 4.20941200 | -0.62075700 |
| C | 1.13504300 | 4.25204500 | -0.26239400 |
| C | 1.56443700 | 2.88226900 | -0.07680900 |
| N | 0.50461800 | 2.03096900 | -0.29712100 |
| C | 2.87879200 | 2.49924100 | 0.21409900 |
| C | 3.38224800 | 1.19440200 | 0.29942000 |
| C | 4.76359000 | 0.82888500 | 0.55729400 |
| C | 4.81536600 | -0.53586800 | 0.55430200 |
| C | 3.46556100 | -1.00220600 | 0.29493800 |
| N | 2.62936300 | 0.06643800 | 0.14199100 |
| C | 3.05514200 | -2.33888700 | 0.21437800 |
| C | 1.76397100 | -2.80689200 | -0.05178200 |
| C | 1.41742700 | -4.20238600 | -0.21282500 |
| C | 0.09250800 | -4.24564900 | -0.53843000 |
| C | -0.37910400 | -2.87667200 | -0.56809400 |
| N | 0.64746600 | -2.02462000 | -0.25401900 |
| C | -1.66831600 | -2.50819700 | -0.99663100 |
| H | -3.86425700 | -1.52070100 | -2.57985900 |
| H | -3.93856500 | 1.17004700 | -2.64421500 |
| H | -0.83879400 | 5.03447200 | -0.86439100 |
| H | 1.77401700 | 5.11946800 | -0.15590900 |
| H | 5.57357300 | 1.53071200 | 0.71065900 |
| H | 5.67574400 | -1.17548500 | 0.70487500 |
| H | 2.11210100 | -5.02666200 | -0.11308900 |
| H | -0.51774900 | -5.11267800 | -0.75707000 |
| H | -2.49351700 | 3.11022100 | -1.47890600 |
| H | 3.59249800 | 3.30571400 | 0.35648400 |
| H | 3.82301900 | -3.09518000 | 0.34950100 |
| H | -2.29646600 | -3.31792800 | -1.35702400 |
| Co | 0.51479500 | 0.00225500 | 0.20506000 |
| H | 0.49491300 | 0.07090700 | 1.93163100 |
| H | -1.88237400 | -0.05327800 | 0.52127600 |
| O | -2.33879000 | -0.06773200 | 1.81133300 |
| C | -3.61563600 | -0.01305300 | 1.92138000 |
| O | -4.01767700 | -0.00859400 | 3.24293900 |
| O | -4.46549700 | 0.03203200 | 1.01975100 |

| | | | |
|---|---|---|---|
| H | -4.98727800 | 0.03505100 | 3.23872600 |

**[4]TS11**

| | | | |
|---|---|---|---|
| C | -3.56040600 | -0.47415200 | 0.50995800 |
| C | -4.81069200 | 0.22033300 | 0.76612200 |
| C | -4.54012200 | 1.55587500 | 0.69169000 |
| C | -3.12424300 | 1.67933400 | 0.38976600 |
| N | -2.56284900 | 0.43677400 | 0.30945900 |
| C | -2.44268500 | 2.87954600 | 0.16409400 |
| C | -1.11145900 | 3.02959100 | -0.25323300 |
| C | -0.47111700 | 4.30342000 | -0.52524000 |
| C | 0.80055600 | 4.02441900 | -0.93444200 |
| C | 0.93825800 | 2.58045800 | -0.91129500 |
| N | -0.23328700 | 2.01138400 | -0.49349500 |
| C | 2.08654900 | 1.88288400 | -1.29959200 |
| C | 2.24910400 | 0.49508700 | -1.35618600 |
| C | 3.39950700 | -0.19247200 | -1.91309300 |
| C | 3.13045100 | -1.52858400 | -1.84300100 |
| C | 1.81445200 | -1.65672500 | -1.24057400 |
| N | 1.32390900 | -0.41646600 | -0.92898400 |
| C | 1.12679800 | -2.85902100 | -1.03770800 |
| C | -0.19438300 | -3.01071700 | -0.60021100 |
| C | -0.88303300 | -4.28186900 | -0.47891600 |
| C | -2.15370100 | -4.00095200 | -0.06749200 |
| C | -2.24191300 | -2.55791300 | 0.05966900 |
| N | -1.04183800 | -1.99128900 | -0.26481400 |
| C | -3.40228900 | -1.86112400 | 0.42870600 |
| H | -5.76330500 | -0.25997600 | 0.95654200 |
| H | -5.22737500 | 2.38552800 | 0.80927800 |
| H | -0.94444300 | 5.27274500 | -0.42171400 |
| H | 1.57767200 | 4.71871600 | -1.23090000 |
| H | 4.28463100 | 0.29380400 | -2.30399300 |
| H | 3.74818800 | -2.35491300 | -2.17414200 |
| H | -0.44473600 | -5.25037900 | -0.68882300 |
| H | -2.96475400 | -4.69368400 | 0.12392700 |
| H | -3.01630100 | 3.79542100 | 0.28623100 |
| H | 2.93292000 | 2.48282700 | -1.62316200 |
| H | 1.65920800 | -3.77197400 | -1.29424100 |
| H | -4.28343200 | -2.46469300 | 0.63368700 |
| Co | -0.51177400 | 0.02676700 | 0.20191200 |
| H | 0.22338400 | -0.04281400 | 1.67061100 |
| H | 2.35611800 | -0.76740700 | 0.96419300 |
| O | 2.81901000 | -0.92514600 | 1.81355300 |
| C | 3.75711800 | -0.01433500 | 1.98802500 |
| O | 4.33388900 | -0.20851300 | 3.20625000 |
| O | 4.10781500 | 0.86808000 | 1.22936400 |

| | | | |
|---|---|---|---|
| H | 4.99234800 | 0.49739800 | 3.27011800 |

**$^{2}$TS12**

| | | | |
|---|---|---|---|
| C | -2.77149600 | -1.14605300 | -0.75491200 |
| C | -4.07401800 | -0.68285700 | -1.12549200 |
| C | -4.07392300 | 0.68349400 | -1.12542300 |
| C | -2.77133100 | 1.14647000 | -0.75480700 |
| N | -1.94233400 | 0.00013600 | -0.45720900 |
| C | -2.31814000 | 2.42925100 | -0.69326900 |
| C | -0.97388500 | 2.83492800 | -0.51586100 |
| C | -0.57247100 | 4.19838500 | -0.43202500 |
| C | 0.80385400 | 4.20493200 | -0.34152800 |
| C | 1.22187700 | 2.84088300 | -0.37934700 |
| N | 0.13072200 | 1.99594900 | -0.48002400 |
| C | 2.56174200 | 2.41738800 | -0.34164500 |
| C | 2.98018000 | 1.10374900 | -0.37819100 |
| C | 4.36238500 | 0.67905100 | -0.28173400 |
| C | 4.36228000 | -0.67969000 | -0.28168400 |
| C | 2.98001100 | -1.10418300 | -0.37811900 |
| N | 2.14822300 | -0.00015700 | -0.46836900 |
| C | 2.56137100 | -2.41775600 | -0.34153800 |
| C | 1.22144300 | -2.84104700 | -0.37930800 |
| C | 0.80322200 | -4.20503500 | -0.34151900 |
| C | -0.57309500 | -4.19829200 | -0.43211600 |
| C | -0.97430800 | -2.83477800 | -0.51597500 |
| N | 0.13041500 | -1.99595300 | -0.48006300 |
| C | -2.31849700 | -2.42890500 | -0.69343600 |
| H | -4.88940500 | -1.34047000 | -1.39903600 |
| H | -4.88921700 | 1.34124800 | -1.39890200 |
| H | -1.25162100 | 5.04175300 | -0.44352800 |
| H | 1.47201500 | 5.05394500 | -0.27008100 |
| H | 5.20512800 | 1.35545700 | -0.21183900 |
| H | 5.20492100 | -1.35621800 | -0.21174000 |
| H | 1.47125900 | -5.05414200 | -0.27002800 |
| H | -1.25236300 | -5.04156400 | -0.44366900 |
| H | -3.04862200 | 3.21962900 | -0.84145900 |
| H | 3.32396600 | 3.18681900 | -0.26407100 |
| H | 3.32347200 | -3.18730200 | -0.26390700 |
| H | -3.04908600 | -3.21917000 | -0.84169300 |
| Co | 0.18733400 | -0.00000600 | -0.48703400 |
| H | -1.79718500 | 0.00007500 | 0.58144900 |
| H | 0.29187400 | 0.00002500 | 1.07841200 |
| O | 0.41596800 | 0.00013700 | 2.38209600 |
| C | -0.75003900 | 0.00002500 | 2.92991500 |
| O | -0.68114200 | -0.00007900 | 4.29195100 |
| O | -1.85639900 | 0.00001800 | 2.35954700 |

| | | | |
|---|---|---|---|
| H | -1.59273200 | -1.14605300 | -0.75491200 |

**[4]TS13**

| | | | |
|---|---|---|---|
| C | 0.07622800 | 2.97068700 | -1.12134800 |
| C | 0.36410000 | 4.30578200 | -0.68898400 |
| C | 0.36410000 | 4.30578200 | 0.68898400 |
| C | 0.07622800 | 2.97068700 | 1.12134800 |
| N | -0.08684700 | 2.18904100 | 0.00000000 |
| C | -0.00675300 | 2.44737700 | 2.40864600 |
| C | -0.08164400 | 1.09794100 | 2.77360500 |
| C | -0.07570300 | 0.68725300 | 4.16289300 |
| C | -0.04585900 | -0.67094300 | 4.18062200 |
| C | -0.03386300 | -1.10661500 | 2.80223000 |
| N | -0.07375000 | -0.02124900 | 1.95936100 |
| C | 0.05899700 | -2.44173200 | 2.41097800 |
| C | 0.12994200 | -2.90062000 | 1.09911100 |
| C | 0.24512800 | -4.28151700 | 0.68163600 |
| C | 0.24512800 | -4.28151700 | -0.68163600 |
| C | 0.12994200 | -2.90062000 | -1.09911100 |
| N | 0.07103100 | -2.08741400 | 0.00000000 |
| C | 0.05899700 | -2.44173200 | -2.41097800 |
| C | -0.03386300 | -1.10661500 | -2.80223000 |
| C | -0.04585900 | -0.67094300 | -4.18062200 |
| C | -0.07570300 | 0.68725300 | -4.16289300 |
| C | -0.08164400 | 1.09794100 | -2.77360500 |
| N | -0.07375000 | -0.02124900 | -1.95936100 |
| C | -0.00675300 | 2.44737700 | -2.40864600 |
| H | 0.57272400 | 5.13982200 | -1.34618600 |
| H | 0.57272400 | 5.13982200 | 1.34618600 |
| H | -0.07494900 | 1.37098600 | 5.00168600 |
| H | -0.01565100 | -1.33350500 | 5.03556200 |
| H | 0.31413700 | -5.12786200 | 1.35250400 |
| H | 0.31413700 | -5.12786200 | -1.35250400 |
| H | -0.01565100 | -1.33350500 | -5.03556200 |
| H | -0.07494900 | 1.37098600 | -5.00168600 |
| H | 0.05127700 | 3.15691600 | 3.22841100 |
| H | 0.09591200 | -3.18389400 | 3.20242600 |
| H | 0.09591200 | -3.18389400 | -3.20242600 |
| H | 0.05127700 | 3.15691600 | -3.22841100 |
| Co | -0.20778200 | -0.13503400 | 0.00000000 |
| H | -1.70450000 | 0.48363200 | 0.00000000 |
| H | -0.99596200 | 1.41209500 | 0.00000000 |

**[1]TS14**

| | | | |
|---|---|---|---|
| C | 2.88302000 | 1.11714700 | -0.02580700 |
| C | 4.25553900 | 0.68363800 | -0.05829300 |
| C | 4.25551300 | -0.68379600 | -0.05829200 |
| C | 2.88297800 | -1.11725300 | -0.02580600 |
| N | 2.06225700 | -0.00003700 | -0.02141300 |
| C | 2.42857500 | -2.43261100 | -0.01494600 |
| C | 1.09865100 | -2.86357200 | -0.00755800 |
| C | 0.67741100 | -4.24584200 | -0.00152700 |
| C | -0.68729800 | -4.24276300 | -0.00511500 |
| C | -1.10533100 | -2.85901800 | -0.01448100 |
| N | -0.00289100 | -2.04180000 | -0.01995200 |
| C | -2.43417500 | -2.42325500 | -0.01716100 |
| C | -2.87744400 | -1.10122400 | -0.01609400 |
| C | -4.26435900 | -0.68099800 | -0.04806900 |
| C | -4.26433400 | 0.68115900 | -0.04806900 |
| C | -2.87740300 | 1.10133300 | -0.01609300 |
| N | -2.05841400 | 0.00003900 | 0.00680300 |
| C | -2.43408300 | 2.42334700 | -0.01716000 |
| C | -1.10522300 | 2.85905900 | -0.01448000 |
| C | -0.68713900 | 4.24278800 | -0.00511400 |
| C | 0.67757000 | 4.24581600 | -0.00152700 |
| C | 1.09875900 | 2.86353100 | -0.00755800 |
| N | -0.00281400 | 2.04180000 | -0.01995200 |
| C | 2.42866700 | 2.43252200 | -0.01494800 |
| H | 5.10887900 | 1.34841300 | -0.08980800 |
| H | 5.10882800 | -1.34860300 | -0.08980700 |
| H | 1.34483700 | -5.09785300 | 0.00317500 |
| H | -1.35784800 | -5.09239400 | -0.00371900 |
| H | -5.11444000 | -1.35056700 | -0.07161200 |
| H | -5.11438900 | 1.35076000 | -0.07161100 |
| H | -1.35765700 | 5.09244400 | -0.00371800 |
| H | 1.34502700 | 5.09780300 | 0.00317400 |
| H | 3.19043800 | -3.20661200 | -0.01284000 |
| H | -3.19825800 | -3.19519200 | -0.02540600 |
| H | -3.19813700 | 3.19531200 | -0.02540500 |
| H | 3.19055800 | 3.20649500 | -0.01284200 |
| Co | -0.02633300 | -0.00000100 | 0.08006900 |
| H | 1.07681700 | -0.00002600 | 1.12874800 |

**[4]1pt**

| | | | |
|---|---|---|---|
| C | 2.89485100 | 1.15264300 | -0.02024600 |
| C | 4.18264200 | 0.69361200 | -0.34743800 |
| C | 4.18336800 | -0.68937500 | -0.34738900 |
| C | 2.89603300 | -1.14971700 | -0.02024900 |
| N | 2.04512700 | 0.00102600 | 0.25378500 |
| C | 2.42831700 | -2.44141200 | 0.03012800 |
| C | 1.08828700 | -2.81983500 | 0.10716900 |
| C | 0.67294500 | -4.19931100 | 0.15772900 |
| C | -0.68558400 | -4.20153500 | 0.12765100 |
| C | -1.10200000 | -2.82336800 | 0.05644600 |
| N | -0.00891100 | -1.98181500 | 0.05778200 |
| C | -2.42380100 | -2.41811100 | -0.03166400 |
| C | -2.84197400 | -1.09710200 | -0.09615900 |
| C | -4.21995900 | -0.68222900 | -0.17847100 |
| C | -4.22066100 | 0.67793700 | -0.17842800 |
| C | -2.84310600 | 1.09422600 | -0.09613500 |
| N | -2.01044000 | -0.00101300 | -0.05656800 |
| C | -2.42627800 | 2.41565800 | -0.03164300 |
| C | -1.10489100 | 2.82226200 | 0.05643700 |
| C | -0.68986600 | 4.20084300 | 0.12766100 |
| C | 0.66866600 | 4.19998700 | 0.15769600 |
| C | 1.08539800 | 2.82092600 | 0.10718100 |
| N | -0.01095000 | 1.98180800 | 0.05779200 |
| C | 2.42581400 | 2.44385400 | 0.03013300 |
| H | 5.00945500 | 1.34365400 | -0.60085100 |
| H | 5.01084100 | -1.33857000 | -0.60082400 |
| H | 1.34925900 | -5.04200900 | 0.20308500 |
| H | -1.36005400 | -5.04681200 | 0.14300100 |
| H | -5.06286800 | -1.35826000 | -0.22348100 |
| H | -5.06426700 | 1.35310100 | -0.22344200 |
| H | -1.36518800 | 5.04543900 | 0.14305900 |
| H | 1.34412900 | 5.04336800 | 0.20298600 |
| H | 3.16270800 | -3.23530300 | -0.05697300 |
| H | -3.18651100 | -3.18902500 | -0.04808400 |
| H | -3.18977500 | 3.18579500 | -0.04804300 |
| H | 3.15939500 | 3.23849200 | -0.05699600 |
| Co | -0.05946900 | -0.00002100 | 0.00162600 |
| H | 1.88555300 | 0.00095400 | 1.27064600 |

**¹2-$CO_2$-$H_2O$**

| | | | |
|---|---|---|---|
| C | -1.38213400 | 2.69155900 | -0.12299900 |
| C | -2.69855200 | 3.27751400 | -0.08273100 |
| C | -3.58562400 | 2.24520700 | -0.02478300 |
| C | -2.81067000 | 1.02964200 | -0.03276700 |
| N | -1.46137700 | 1.31364600 | -0.09962200 |
| C | -3.36139400 | -0.24214200 | 0.00372600 |
| C | -2.63364400 | -1.42189000 | -0.03398600 |
| C | -3.22953900 | -2.73509800 | -0.03132000 |
| C | -2.20559700 | -3.63147200 | -0.08762900 |
| C | -0.98481900 | -2.86495800 | -0.12239200 |
| N | -1.25777000 | -1.51271600 | -0.09392500 |
| C | 0.28361400 | -3.42198800 | -0.17331000 |
| C | 1.45925000 | -2.68774700 | -0.21265400 |
| C | 2.77591200 | -3.27613600 | -0.25693300 |
| C | 3.66428800 | -2.24453800 | -0.26918500 |
| C | 2.88837600 | -1.02862600 | -0.23560400 |
| N | 1.54005400 | -1.31137100 | -0.21010400 |
| C | 3.44203400 | 0.24247400 | -0.23241600 |
| C | 2.71279800 | 1.42167700 | -0.22161900 |
| C | 3.30855900 | 2.73533300 | -0.25252900 |
| C | 2.28293700 | 3.63052500 | -0.24273400 |
| C | 1.06277500 | 2.86178200 | -0.20094300 |
| N | 1.33771500 | 1.51055400 | -0.19514900 |
| C | -0.20498900 | 3.42231500 | -0.16918300 |
| H | -2.89442100 | 4.34181500 | -0.09962200 |
| H | -4.66672600 | 2.28029600 | 0.01326400 |
| H | -4.29508600 | -2.92213600 | 0.00182000 |
| H | -2.24914200 | -4.71271600 | -0.10802500 |
| H | 2.96907100 | -4.34091100 | -0.27619700 |
| H | 4.74558700 | -2.27891800 | -0.30275200 |
| H | 4.37413000 | 2.92236900 | -0.28532800 |
| H | 2.32341500 | 4.71189500 | -0.26337300 |
| H | -4.44266700 | -0.32040900 | 0.05129600 |
| H | 0.36161900 | -4.50417800 | -0.18326800 |
| H | 4.52425800 | 0.32035200 | -0.25218700 |
| H | -0.28087700 | 4.50464000 | -0.17952800 |
| C | 0.11879000 | 0.00702700 | 1.96183900 |
| O | 1.26597400 | 0.04219000 | 2.45194900 |
| O | -0.99291400 | -0.02430000 | 2.52872100 |
| Co | 0.05021300 | -0.00001200 | 0.00423300 |
| O | -1.02898200 | -0.00971800 | -2.88449400 |
| H | -1.08695000 | -0.81115500 | -2.34062200 |
| H | -1.21515500 | 0.68046500 | -2.22667300 |

**[2]3-$CO_2$-$H_2O$**

| | | | |
|---|---|---|---|
| C | -1.66299700 | 2.55918300 | -0.11613200 |
| C | -3.03888900 | 3.01629300 | -0.12346700 |
| C | -3.82504400 | 1.90970800 | -0.13719300 |
| C | -2.94151900 | 0.76015000 | -0.13636400 |
| N | -1.61742400 | 1.17301200 | -0.14150300 |
| C | -3.38161100 | -0.54241700 | -0.12371000 |
| C | -2.54729600 | -1.67638200 | -0.11355500 |
| C | -3.01551900 | -3.01945300 | -0.10634100 |
| C | -1.89434500 | -3.83152500 | -0.09434000 |
| C | -0.76497400 | -2.96711800 | -0.09491300 |
| N | -1.16327700 | -1.64037500 | -0.11315800 |
| C | 0.57456500 | -3.39914700 | -0.08869100 |
| C | 1.66301100 | -2.55936000 | -0.11599300 |
| C | 3.03890500 | -3.01646700 | -0.12322500 |
| C | 3.82505700 | -1.90988100 | -0.13697000 |
| C | 2.94152900 | -0.76032400 | -0.13630300 |
| N | 1.61743600 | -1.17319400 | -0.14149800 |
| C | 3.38162100 | 0.54224500 | -0.12370100 |
| C | 2.54730900 | 1.67621700 | -0.11363000 |
| C | 3.01553300 | 3.01929000 | -0.10655800 |
| C | 1.89435700 | 3.83136300 | -0.09469900 |
| C | 0.76498700 | 2.96695200 | -0.09507500 |
| N | 1.16329300 | 1.64021200 | -0.11315300 |
| C | -0.57455300 | 3.39897700 | -0.08890200 |
| H | -3.33498200 | 4.05816100 | -0.12024300 |
| H | -4.90637300 | 1.84688000 | -0.14850400 |
| H | -4.05913500 | -3.30918300 | -0.11654000 |
| H | -1.84324300 | -4.91343900 | -0.09124600 |
| H | 3.33500000 | -4.05833400 | -0.11989300 |
| H | 4.90638600 | -1.84705000 | -0.14820200 |
| H | 4.05914900 | 3.30901900 | -0.11681300 |
| H | 1.84325400 | 4.91327800 | -0.09178700 |
| H | -4.45403300 | -0.71353600 | -0.12090000 |
| H | 0.75712100 | -4.46934800 | -0.07097100 |
| H | 4.45404400 | 0.71336100 | -0.12087100 |
| H | -0.75711300 | 4.46918000 | -0.07132400 |
| C | -0.00001600 | -0.00012500 | 1.97894400 |
| O | 1.11800700 | 0.14610000 | 2.53081200 |
| O | -1.11804700 | -0.14637600 | 2.53078500 |
| Co | 0.00000700 | -0.00009500 | 0.04600800 |
| O | -0.00008600 | 0.00157000 | -3.39003800 |
| H | -0.01390400 | -0.75942100 | -3.98585800 |
| H | 0.01380400 | 0.76596100 | -3.98145900 |

**[2]3pt-$CO_2$-$H_2O$**

| | | | |
|---|---|---|---|
| C | 2.93277600 | 1.07092400 | -0.14191200 |
| C | 4.24126400 | 0.58606400 | -0.47250600 |
| C | 4.21453500 | -0.77940500 | -0.46768500 |
| C | 2.88723300 | -1.21050200 | -0.13832400 |
| N | 2.08392000 | -0.05294800 | 0.12858900 |
| C | 2.40273900 | -2.48429400 | -0.09615000 |
| C | 1.03927000 | -2.85685100 | -0.01729000 |
| C | 0.60083500 | -4.21232100 | 0.01981300 |
| C | -0.77622000 | -4.19142000 | -0.03644100 |
| C | -1.16053300 | -2.81907700 | -0.11664800 |
| N | -0.04664600 | -2.00324600 | -0.08848600 |
| C | -2.48298700 | -2.36919600 | -0.26086900 |
| C | -2.86445300 | -1.04858100 | -0.37581100 |
| C | -4.24129600 | -0.59646100 | -0.41914500 |
| C | -4.21389300 | 0.76142900 | -0.39687900 |
| C | -2.81985600 | 1.15592300 | -0.34098400 |
| N | -2.01023800 | 0.03736500 | -0.38949800 |
| C | -2.38516200 | 2.45607400 | -0.18813100 |
| C | -1.04441700 | 2.84788900 | -0.04282100 |
| C | -0.60142300 | 4.20189700 | 0.04493600 |
| C | 0.77629500 | 4.16457300 | 0.07478200 |
| C | 1.15652600 | 2.79248600 | 0.01798100 |
| N | 0.03421400 | 1.98580500 | -0.03683400 |
| C | 2.50161500 | 2.36350700 | -0.08987700 |
| H | 5.07543600 | 1.22861100 | -0.72395700 |
| H | 5.02282000 | -1.45564200 | -0.71530800 |
| H | 1.26065000 | -5.06935200 | 0.06913800 |
| H | -1.46498400 | -5.02648100 | -0.04767400 |
| H | -5.09940800 | -1.25633600 | -0.44163700 |
| H | -5.04468500 | 1.45576700 | -0.39747900 |
| H | -1.25442500 | 5.06523200 | 0.05305400 |
| H | 1.47226300 | 4.99277500 | 0.11989100 |
| H | 3.12001500 | -3.29281300 | -0.20289200 |
| H | -3.26549700 | -3.12156500 | -0.26445500 |
| H | -3.13618700 | 3.23961900 | -0.16928700 |
| H | 3.25009100 | 3.14225800 | -0.20312100 |
| H | 1.80124500 | -0.04705200 | 1.16686000 |
| C | -0.20936100 | -0.03725400 | 1.87465300 |
| O | 0.93432400 | -0.04341700 | 2.45416400 |
| O | -1.31752400 | -0.04854600 | 2.44058100 |
| Co | -0.06515700 | -0.00878000 | -0.06070800 |
| O | 0.31188100 | 0.14172200 | -2.34137200 |
| H | 0.96363200 | 0.85963900 | -2.34289600 |

| | | | |
|---|---|---|---|
| H | -0.52083100 | 0.58305300 | -2.56864700 |

**[1]4-$H_2O$**

| | | | |
|---|---|---|---|
| C | -2.49274900 | 1.73718000 | -0.11179500 |
| C | -3.92697000 | 1.65590600 | -0.19847000 |
| C | -4.24498600 | 0.33398700 | -0.30291600 |
| C | -3.00689100 | -0.39875400 | -0.26482100 |
| N | -1.93844100 | 0.47308600 | -0.15498800 |
| C | -2.90825800 | -1.78210200 | -0.26581000 |
| C | -1.71753900 | -2.48710200 | -0.16554500 |
| C | -1.63824600 | -3.92491400 | -0.10296600 |
| C | -0.31770500 | -4.24451200 | -0.00723200 |
| C | 0.41400900 | -3.00329400 | -0.02834700 |
| N | -0.45421200 | -1.93610400 | -0.11492200 |
| C | 1.79788100 | -2.91561700 | -0.03279500 |
| C | 2.50774200 | -1.73049500 | -0.15491100 |
| C | 3.94771200 | -1.64984500 | -0.20078300 |
| C | 4.26315600 | -0.32949700 | -0.30354100 |
| C | 3.01600400 | 0.39665800 | -0.30680800 |
| N | 1.95461100 | -0.47489400 | -0.24600900 |
| C | 2.92452000 | 1.78046300 | -0.30256200 |
| C | 1.73748500 | 2.49080700 | -0.20517200 |
| C | 1.65963900 | 3.92903100 | -0.13940100 |
| C | 0.33990800 | 4.24806100 | -0.03480900 |
| C | -0.39193600 | 3.00569800 | -0.04837400 |
| N | 0.47633400 | 1.94080200 | -0.15274200 |
| C | -1.77629600 | 2.92099500 | -0.02343200 |
| H | -4.58758000 | 2.51290100 | -0.18720400 |
| H | -5.22158200 | -0.12401700 | -0.38835700 |
| H | -2.49647200 | -4.58339100 | -0.13237800 |
| H | 0.14210300 | -5.22211400 | 0.05396200 |
| H | 4.60804900 | -2.50615800 | -0.15954500 |
| H | 5.23910400 | 0.13433200 | -0.36128600 |
| H | 2.51760600 | 4.58763100 | -0.17309800 |
| H | -0.12000900 | 5.22522200 | 0.03253700 |
| H | -3.82854400 | -2.35325200 | -0.32277400 |
| H | 2.36271400 | -3.83907800 | 0.03594300 |
| H | 3.84840100 | 2.34740200 | -0.34333100 |
| H | -2.33752300 | 3.84758400 | 0.03134500 |
| C | 0.12763800 | 0.02800600 | 1.79972500 |
| O | 1.14923500 | 0.12286100 | 2.45499700 |
| O | -1.05118400 | -0.07517000 | 2.48166900 |
| H | -1.77227600 | -0.07517500 | 1.82784700 |
| Co | 0.02373300 | 0.00280100 | -0.10006000 |
| O | -0.09144200 | -0.02913300 | -2.32499300 |
| H | -0.94186300 | 0.40473600 | -2.49951400 |

| | | | |
|---|---|---|---|
| H | -0.25853700 | -0.96491300 | -2.52069500 |

**$^{1}$2pt'-$H_2O$**

| | | | |
|---|---|---|---|
| C | 1.51811900 | -2.64080900 | -0.08989300 |
| C | 2.85358300 | -3.18501300 | -0.09116200 |
| C | 3.70877200 | -2.12545000 | -0.09661600 |
| C | 2.89507900 | -0.93487900 | -0.09905800 |
| N | 1.55747600 | -1.26389700 | -0.08791800 |
| C | 3.40693800 | 0.35396800 | -0.10734000 |
| C | 2.63442100 | 1.50554900 | -0.11036900 |
| C | 3.17689700 | 2.84128900 | -0.11156400 |
| C | 2.11648000 | 3.69579400 | -0.11488200 |
| C | 0.92644400 | 2.88195300 | -0.11625200 |
| N | 1.25675100 | 1.54395200 | -0.10933100 |
| C | -0.36271000 | 3.39292100 | -0.12186600 |
| C | -1.51458200 | 2.62073400 | -0.12076800 |
| C | -2.85000800 | 3.16423800 | -0.12147400 |
| C | -3.70532700 | 2.10448800 | -0.11806800 |
| C | -2.89223300 | 0.91393300 | -0.11507500 |
| N | -1.55402000 | 1.24330200 | -0.11386600 |
| C | -3.40319400 | -0.37516800 | -0.10906700 |
| C | -2.62985100 | -1.52624700 | -0.09829600 |
| C | -3.17298200 | -2.86211700 | -0.09353000 |
| C | -2.11272200 | -3.71643800 | -0.08782300 |
| C | -0.92278900 | -2.90187000 | -0.08864600 |
| N | -1.25287000 | -1.56443100 | -0.08790100 |
| C | 0.36631200 | -3.41315000 | -0.08830400 |
| H | 3.08108500 | -4.24303300 | -0.08689500 |
| H | 4.79101500 | -2.12455100 | -0.09801000 |
| H | 4.23466500 | 3.06995600 | -0.10833100 |
| H | 2.11514000 | 4.77803800 | -0.11536000 |
| H | -3.07787900 | 4.22218800 | -0.12261500 |
| H | -4.78756600 | 2.10391000 | -0.11554700 |
| H | -4.23083200 | -3.09044200 | -0.09387600 |
| H | -2.11096500 | -4.79866500 | -0.08222900 |
| H | 4.48558100 | 0.46955700 | -0.11135700 |
| H | -0.47834000 | 4.47156300 | -0.12710100 |
| H | -4.48177800 | -0.49129200 | -0.11263300 |
| H | 0.48188800 | -4.49181700 | -0.08856000 |
| Co | 0.00181500 | -0.01081300 | -0.11911500 |
| H | 0.00546700 | -0.02694100 | -1.54662700 |
| O | -0.01715000 | 0.08469300 | 2.10491200 |
| H | 0.65038600 | 0.76396400 | 2.29063900 |
| H | -0.86090500 | 0.52710700 | 2.28873800 |

**$^{2}$3pt'-H$_2$O**

| | | | |
|---|---|---|---|
| C | 1.21283800 | 2.81424800 | -0.10831100 |
| C | 0.86153600 | 4.19167400 | -0.09675300 |
| C | -0.52087500 | 4.25061200 | -0.06742900 |
| C | -0.98807400 | 2.90800000 | -0.06448100 |
| N | 0.07681200 | 2.02320300 | -0.08097900 |
| C | -2.34522500 | 2.53418800 | -0.05130100 |
| C | -2.80093600 | 1.23723000 | -0.06515400 |
| C | -4.20321300 | 0.87101900 | -0.06899800 |
| C | -4.26205600 | -0.48463800 | -0.09898600 |
| C | -2.89656700 | -0.97082600 | -0.10968300 |
| N | -2.01254200 | 0.09676300 | -0.08038400 |
| C | -2.55585100 | -2.30272900 | -0.13419500 |
| C | -1.23617500 | -2.79289100 | -0.12842200 |
| C | -0.88411000 | -4.16955000 | -0.12480500 |
| C | 0.49913200 | -4.22834700 | -0.10859700 |
| C | 0.96688000 | -2.88659700 | -0.10610200 |
| N | -0.09921800 | -2.00140800 | -0.11337100 |
| C | 2.32406000 | -2.51178900 | -0.10072700 |
| C | 2.77992900 | -1.21500500 | -0.10751200 |
| C | 4.18173100 | -0.84793400 | -0.11451600 |
| C | 4.24077700 | 0.50819400 | -0.13093800 |
| C | 2.87620800 | 0.99539000 | -0.12924200 |
| N | 1.99118900 | -0.07329500 | -0.11088000 |
| C | 2.53330800 | 2.32644100 | -0.13317000 |
| H | 1.57247800 | 5.00850700 | -0.10569600 |
| H | -1.15974200 | 5.12482600 | -0.04988200 |
| H | -5.01784500 | 1.58442700 | -0.05097200 |
| H | -5.13528300 | -1.12512200 | -0.10975000 |
| H | -1.59454700 | -4.98684600 | -0.12828600 |
| H | 1.13772200 | -5.10289300 | -0.09926400 |
| H | 4.99669200 | -1.56116200 | -0.10768200 |
| H | 5.11444700 | 1.14811300 | -0.13875900 |
| H | -3.08250900 | 3.33087700 | -0.03780500 |
| H | -3.35988300 | -3.03189400 | -0.15010400 |
| H | 3.06154900 | -3.30837400 | -0.09673100 |
| H | 3.33605700 | 3.05705100 | -0.14841200 |
| Co | -0.01255900 | 0.01276700 | -0.13311700 |
| H | -0.03013700 | 0.03267500 | -1.56496800 |
| O | 0.12811100 | -0.13273200 | 2.12814700 |
| H | -0.01183300 | -1.09174400 | 2.18308700 |
| H | 1.09344500 | -0.03827000 | 2.16945600 |
